# Biomass Demineralization: A Critical Need for Future Biorefineries

**DOI:** 10.1021/acs.chemrev.5c00784

**Published:** 2026-04-08

**Authors:** Leoncio Santiago-Martínez, Styliani Avraamidou, Alejandro Ayala-Cortés, Ruth Azike, Santanu Bakshi, Chumki Banik, Ezra Bar-Ziv, Emmanuel Canales, Torren Carlson, James A. Dumesic, Jordan Lee Klinger, Jung Min Lee, Fei Long, Armando G. McDonald, Sonali Mohapatra, Rebecca G. Ong, Luisiana Fabiola Palomo Gonzalez, Xuejun Pan, Nepu Saha, Marco Antonio Sanchez Castillo, Sandip K. Singh, Charles M. Sorensen, Bala Subramaniam, Meng-Lin Tsai, Reid C. Van Lehn, Wenjia Wang, Victor M. Zavala, George W. Huber

**Affiliations:** † Department of Chemical and Biological Engineering, 5228 University of Wisconsin−Madison, Madison, Wisconsin 53706, United States; ‡ Department of Forest, Rangeland, and Fire Sciences, 5640 University of Idaho, Moscow, Idaho 83844, United States; § Bioeconomy Institute, Iowa State University, Ames, Iowa 50011, United States; ∥ Department of Mechanical and Aerospace Engineering, 3968 Michigan Technological University, Houghton, Michigan 49931, United States; ⊥ Anellotech Inc., Pearl River, New York 10965, United States; # Energy and Environment Science & Technology Directorate, 17212 Idaho National Laboratory, Idaho Falls, Idaho 83415, United States; ⊗ Department of Biological Systems Engineering, University of Wisconsin−Madison, Madison, Wisconsin 53706, United States; ◆ Department of Chemical Engineering, Michigan Technological University, Houghton, Michigan 49931, United States; ◇ Facultad de Ciencias Químicas, Universidad Autónoma de San Luis Potosí, San Luis Potosí, San Luis Potosí 78210, México; ● Center for Environmentally Beneficial Catalysis, University of Kansas, Lawrence, Kansas 66047, United States; ○ ChemTero Consultants, Fort Myers, Florida 33908, United States; ¶ Department of Chemical and Petroleum Engineering, University of Kansas, Lawrence, Kansas 66045, United States

## Abstract

Biomass contains up to 14 essential elements that serve as nutrients for plant growth and development, including photosynthesis and enzyme functionalities. These elements in different chemical forms (e.g., minerals) constitute the inorganic fraction of biomass and can cause operational issues in thermal and biochemical biomass conversion technologies. In biomass gasification and pyrolysis processes, for instance, inorganics can cause fouling, tar formation, and corrosion. In catalytic and biochemical processes, inorganics can poison catalysts, alter biochemical pathways, and modify product yields and selectivity. This review provides an overview of the inorganic content in biomass feedstocks, the critical role inorganics play in plant biochemistry, the effect that inorganics have in various thermochemical and biochemical biomass conversion technologies, and different approaches to remove them from biomass. We provide recommendations for future research, focusing on developing technologies to effectively remove inorganics from biomass, using the inorganics to improve soil quality and for alternative applications, and designing biorefineries to convert demineralized biomass obtained from diverse sources.

## Graphical abstract

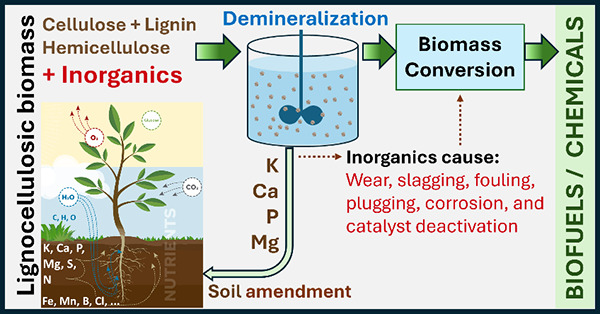

## 1 Introduction

Biomass-derived fuels and chemicals are critical to help society transition to a more sustainable and circular economy. Agriculture is the only major industry that uses carbon dioxide and water as feedstocks via photosynthesis^1,2^ to produce fructose/glucose.^3^ From glucose, other sugars and polysaccharides, lipids, polyphenolics (lignin and tannin), and amino acids/proteins are formed into biomass. These components are stored in the plant mostly as simple carbohydrates, pectin, hemicellulose, cellulose, starches, lignin, and to a smaller extent in fat and proteins.^1,3,4^ In oil seeds, the carbohydrates are further converted and stored as triglycerides.^4,5^

The main elements contained in lignocellulosic biomass (Figure 1) are carbon (37–53 wt %), oxygen (32–49 wt %), and hydrogen (4–8 wt %), which result from the reaction of carbon dioxide and water by photosynthesis. All other components of biomass are absorbed from soil and soil additives (e.g., fertilizers) through the root system (Figure 2). Nitrogen (0.05–1.5 wt %) is primarily present in biomass as amino acids and proteins, which play a vital role in the biochemistry that occurs during plant growth. Inorganic elements such as K, Si, Ca, Cl, Al, Na, Mg, Fe, S, P, Mn, and Zn, are also present in biomass and fill diverse roles in plant growth and development. As shown in Figure 1, agricultural residues, including corn straw, corn stover, rice husk, rice straw, and wheat straw, have a higher overall concentration of inorganic elements than woody biomass. The concentrations of the various inorganics within the biomass are nonuniform and partition among different fractions.^6–9^ For instance, Werkelin et al.^8,9^ reported the concentrations of ash-forming elements (Si, Al, Fe, Ca, Mg, Mn, K, Na, P, S, and Cl) in different parts of spruce, pine, birch, and aspen trees (see Table 3). Leaves, needles, shoots, twigs, and barks tend to have higher concentrations of inorganics compared to wood. For instance, potassium (K) concentrations in wood, bark, twigs, needles, and shoots of pine trees are 407, 3,180, 3,040, 4,770, and 8,790 ppm, respectively, while for aspen trees the K concentrations are 370, 4,730, 5,870, and 24,000 ppm for wood, bark, twigs, and leaves, respectively.^9^ Norway spruce bark, stem wood, and stump have 2.43, 0.31, and 0.43 wt % of ash, respectively.^6^ Corn anatomical fractions have variable ash content as follows: leaves (10.4 wt %), sheath (6.9 wt %), nodes (4.0 wt %), husk (3.7 wt %), internodes (3.5 wt %), and cobs (1.5 wt %).^7^

**Figure 1:**
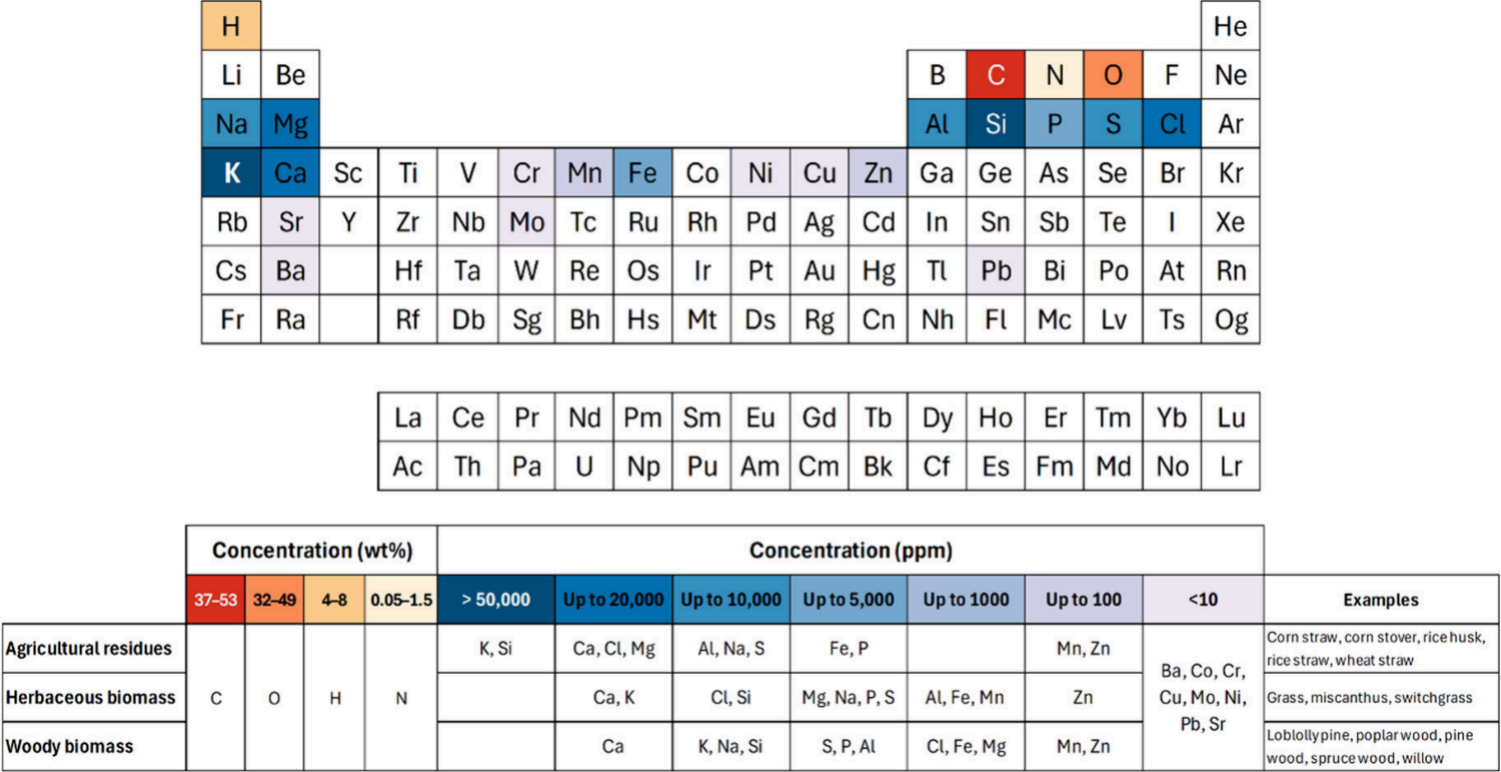
Elemental composition of agricultural, herbaceous, and woody biomass. For a detailed elemental composition of various biomasses, see Tables 3–5 in Section 3.

**Figure 2:**
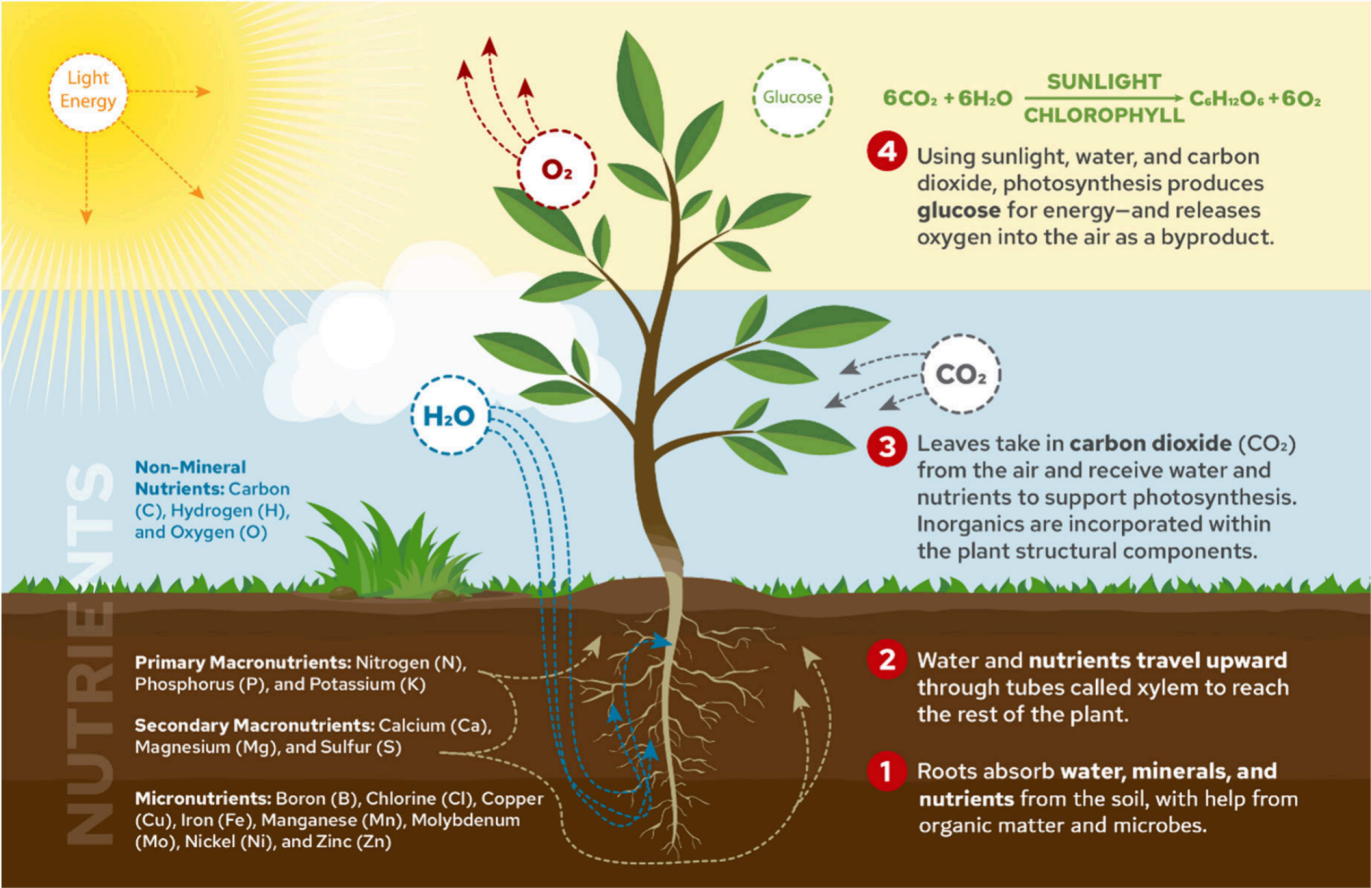
Representation of how nutrients are absorbed by plants through their roots and stored in leaves and stems.

Vassilev et al.^10–13^ have extensively reported the organic and inorganic matter that constitutes various biomasses and clarified related terminology. As detailed in Section 3.1.2, inorganic matter, also referred to as the inorganic fraction of biomass or simply inorganics, includes naturally occurring and introduced matter.^10,12^ Inorganic matter can include (i) crystalline species (minerals such as phosphates, carbonates, silicates, chlorides, sulfates, oxyhydroxides, nitrates, and oxalates), (ii) semicrystalline species (poorly crystallized mineraloids of some silicates, phosphates, and hydroxides), (iii) amorphous matter (glasses and silicates), and (iv) inorganic elements atomically dispersed or bound in the biomass structure via ionic, covalent, or complex interactions.^10,12,14^ In this review, we use these terms as follows:

i) **Inorganic(s)** refer to all inorganic elements and species in biomass, including crystalline, semicrystalline, amorphous, structurally bounded species, and atomically dispersed ions.
ii) **Mineral(s)** refer more specifically to crystalline inorganic and organic (e.g., oxalates) mineral species. However, in Sections 2, 3, and 5, it is common to refer to inorganic elements as mineral elements as well, even though they are in solution, since this is a common practice in agricultural and plant sciences.^15–18^

The inorganic fraction of biomass is mostly nonvolatile. The ash weight percentage (wt %) is typically used to measure the amount of inorganics in biomass (by combusting the biomass in air at 575 °C for 4 h).^19^ This “ashing” process is an inexpensive approach to characterize the amount of inorganics, as oxides, within the biomass structure. Most research on biomass conversion primarily reports on the chemical conversion of the organic fraction of biomass, while often ignoring the role of the inorganic fraction. This simplification creates an important knowledge gap, because the inorganic fraction of biomass causes substantial operational challenges in several conversion technologies, such as thermochemical processes and biochemical and catalytic conversion (Figure 3).^20–27^ For example, minerals can cause wear, slagging, fouling, plugging, and corrosion during biomass thermochemical conversion (i.e., pyrolysis, gasification, and combustion), which renders gasification reactors unable to operate at design targets.^28–32^ The inorganics (e.g., K, Mg, S, Cl, P) can also poison zeolites and other catalysts during biomass-derived intermediates processing,^33,34^ and reduce yields in biochemical conversion.^35^ Minerals (e.g., SiO_2_) have been reported to corrode milling and processing equipment while processing corn stover and rice husks.^36^ Inorganics can be beneficial for certain processes such as microbial fermentation where biomass minerals can provide all of the nutrients required by the microorganisms for growth and fuel production.^37^ During fermentation, micronutrients have a complex interactive effect, with some, such as Mg, serving as essential nutrients and promoting cell growth, while others, such as Cu and Ca, inhibiting growth at high concentrations.^38,39^

**Figure 3:**
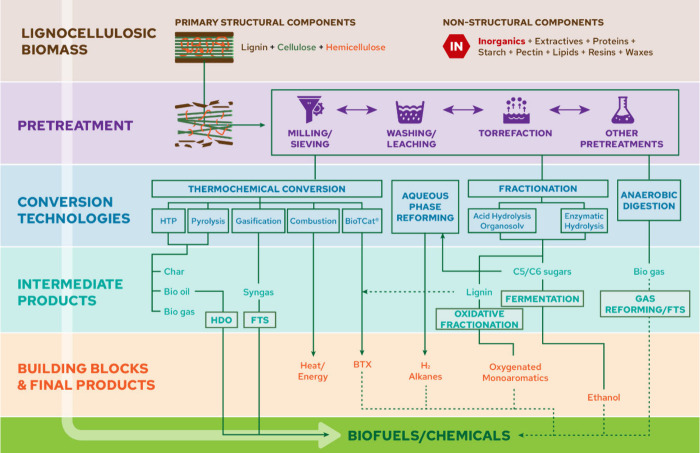
Schematic representation of biomass conversion technologies and their products, that can be affected by the inorganics in biomass.^40–45^ HTP: hydrothermal processing, HDO: hydrodeoxygenation, FTS: Fischer–Trøpsch synthesis, BTX: benzene/toluene/xylenes.

Industrial-scale biorefineries can process between 75 to 10,000 tons of biomass per day.^46^ Hence, the presence of just 100 ppm of an inorganic component in the biomass will result in the processing of 7.5 to 1,000 kg per day of such material. Because of their detrimental effects on biorefinery processes, appropriate technologies are needed to remove inorganics from biorefinery feed streams. The ideal biorefinery would extract these inorganics and return them to soil so they can be reabsorbed by plants.

This review has been motivated by the fact that, despite the challenges posed by biomass inorganics in the operation of biorefining technologies, the technical challenge of how to remove such components from biomass has not been adequately addressed. We have organized this paper as follows. We first discuss the minerals in soil and the critical role they play in plant biochemistry in Section 2. We then analyze the inorganic content of various biomass feedstocks in Section 3. In Sections 4, 5 and 6, we discuss the role of inorganics in biomass thermal conversion, biochemical pathways and catalyst poisoning, respectively. In Section 7, we discuss inorganic removal approaches such as mechanical separation, torrefaction, and acid washing. We additionally discuss the MinFree technology which uses a dilute acid to solubilize inorganics in the aqueous phase while preserving as much of the biomass structure as possible.^34,47^ In Section 8, we summarize the main takeaways from this review, identifying knowledge gaps and technical challenges. We conclude by providing recommendations for future research aimed at designing inorganics removal technologies that facilitate resource-efficient and sustainable biomass conversion technologies for making renewable fuels and chemicals.

## 2 Soil Composition and Minerals Absorption by Plants

### Section Overview

In this section, we describe the role that soil components play in minerals management. Organic and inorganic matter are essential for supplying macronutrients (N, S, P, K, Ca, and Mg) and micronutrients (Fe, Zn, Mn, Cu, Mo, B, Ni, and Cl), while water transfers and delivers the stored nutrients to the plants. We also discuss soil and environmental factors that affect nutrient uptake by plants, such as temperature and soil acidity. These factors influence nutrient absorption because they determine minerals solubility in water and availability. We further discuss some macronutrient ionic species that are more readily available for plant uptake, such as NH_4_^+^, NO_3_^–^, H_2_PO_4_^–^ and HPO_4_^2–^, as well as solubilized K, Ca, and Mg species, and the mechanism by which they are transported from the soil to plant roots.

#### 2.1 Soil Composition

Soil contains about 50% voids or pores by volume, with the remainder being solids (see Figure 4). The four major components of soil are (i) organic matter (5 vol %), which forms during the decay of plant residues, tissues of animal remains, and microbial tissue; (ii) mineral matter (45 vol %) resulting from the disintegration and decomposition of igneous and sedimentary rocks during soil formation; (iii) water (25 vol %) that accumulates from atmospheric deposition and soil physical, chemical, and biological transformations; and (iv) air or gaseous substance (25 vol %) obtained by the atmospheric deposition and different chemical, biological, and microbial reactions within the soil.^48–50^ While the contribution of solids (components 1 and 2 above) is relatively constant for a particular soil over time, water and air (or gas) percentages are highly variable. Soil composition plays a vital role for nutrient management. Organic matter and mineral matter in soil hold and store nutrients, while the water component transfers and delivers the stored nutrients to the plants (Figure 2). Air or gaseous substances in the soil also play a vital role in plant nutrient delivery, as microorganisms use the air to control their biological processes to release nutrients.

**Figure 4:**
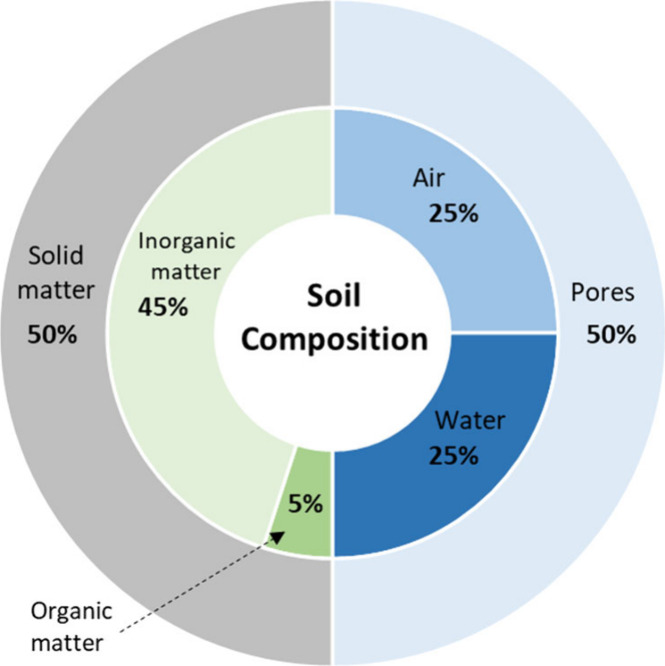
Typical soil composition (vol %).^49,51^ Adapted with permission from Virginia Cooperative Extension^49^ Copyright 2023, Virginia Cooperative Extension, Virginia Polytechnic Institute and State University under CC BY-NC-SA 4.0 license (https://creativecommons.org/licenses/by-nc-sa/4.0/).

Although organic matter contributes only ∼ 5% to soil volume, it is essential for supplying soil minerals or nutrients. In general, as shown in Table 1, soil organic matter consists of three components: (i) plant and animal residues, and living microbial biomass (10 vol %); (ii) detritus or active soil organic matter (10 vol %); and (iii) humus or very stable dark-colored soil organic matter (80 vol %). The living microbes break down the first two components of the soil organic matter, which releases macronutrients such as nitrogen, phosphorus, and potassium. The decomposed product from the first two components forms a very stable C-rich zone called humus.^52^

**Table 1:** Components of Organic and Inorganic Matter in Soil.^52^

Component	vol %
**Soil Organic matter (5 vol % overall)**	
Plant and animal residues and living microbial biomass	10%
Detritus or active soil organic matter	10%
Humus or very stable dark-colored soil organic matter	80%
**Soil mineral matter (45 vol % overall)**	
Igneous rocks	90%
Sedimentary rocks	10%

Soil mineral matter, commonly referred to as the inorganic portion of solid soil, is composed of different minerals of which the primary source is igneous rocks (90–92 vol %) and the secondary source is sedimentary rocks (8–10 vol %). This inorganic portion is important in soil fertility or the ability of soil to supply nutrients (minerals) to plants and serves as a potential site for nutrient storage. Due to the size difference between different minerals, soil mineral matter can be divided into at least three classes: (i) sand (particle size: 0.05–2 mm), which has a low surface area, a gritty texture and permits facile water drainage; (ii) silt (particle size: 0.002–0.05 mm), which has a smooth texture, increased surface area and enhances the soil water holding capacity; and (iii) clay (particle size: <0.002 mm), which has a charged surface to hold nutrients, a sticky texture, and high surface area. The compositions of soil organic matter and biomass mineral matter are related, as the plant and animal residues and active organic matter also exist in the sand and silt fractions; in contrast, humus occurs only in the clay fraction.^50^

The relative proportions of sand, silt, and clay in soil determine the overall soil texture, which regulates the water supply and, thereby, nutrient delivery to the plants. The water supply to plants is usually greater in moderately fine-textured soils than in coarse-textured soils. Physical and chemical weathering also cause the disintegration of rock materials and increase nutrient supply to the plants.^50^

#### 2.2 Factors That Affect Mineral Absorption by Plants

Soil provides two types of nutrients (minerals) that are essential for plant growth: (i) macronutrients (N, S, P, K, Ca, and Mg) that are building blocks of protein and nucleic acids, and (ii) micronutrients (Fe, Zn, Mn, Cu, Mo, B, Ni, and Cl) that are essential for enzyme activity.^53–56^ Si and Na are also considered essential elements for plant structural resistance, as they form part of cell walls and regulate various functions; hence, they are classified as macronutrients for some plants.^54^ Mineral nutrients are transmitted through the plant roots by absorption, which depends on soil, plant, and environmental factors. Soil factors include pH, oxygen concentration, and ion interactions; plant factors include plant growth, variety, aging, and root architecture; and environmental factors include temperature, water availability, seasonality, and CO_2_ levels.^57^

##### 2.2.1 pH Effect

Plant roots absorb mineral nutrients from soil and the chemistry of soils has a direct influence on mineral adsorption. The H^+^ or OH^–^ ions influence the exchange of anions (such as NO_3_^–^, PO_4_^3–^, and SO_4_^2–^) or cations (such as Ca^2+^, Mg^2+^, K^+^, and NH_4_^+^), respectively, from soil solution through plant roots. The solubility and availability of soil macro- and micronutrients are affected by changes in soil pH. At low soil pH (pH <5), the dissolution of Al-, Fe-, and Mn-bearing minerals releases abundant cations, replacing exchangeable base cations such as Ca^2+^, Mg^2+^, and K^+^ and creating further acidity upon dissolution.^58,59^ Moreover, P, another plant macronutrient, tends to bind with Al or Fe to form an insoluble complex, which is not accessible to plant roots. Consequently, metal toxicity and nutrient imbalance can occur, inhibiting mineral absorption and plant growth. At high pH (pH >7.5), the availability of soil macro- and micronutrients such as Ca, Mg, Fe, Mn, Zn, and P decrease,^60^ increasing the root apoplastic (network including cell walls, intercellular spaces, and xylem) pH. This change impairs the pH gradient across the plasma membrane, which decreases mineral absorption and uptake by plant roots.^61^ At higher pH, P tends to bind with Ca and Mg to form insoluble minerals, which plants cannot absorb. The clay portion of soils, which is associated with the soil humus fraction, holds considerable positively charged cations in its negatively charged surface through electrostatic forces.^62^ Depending on the soil pH, these cations can be exchanged with other cations to become plant-available.^62^ The leaching of these cations and the decomposition of soil organic materials are also responsible for the change in soil pH, which causes significant changes in plant mineral absorption. Moreover, the decomposition rates of microorganisms depend on soil pH, which indirectly affects mineral availability or plant absorption. The chemical forms of minerals depend on the soil pH and the plant absorption. Changing the pH changes the mineral content of the soil, but converts the minerals into forms that plants cannot absorb or uptake for growth.^63^ Strategies for maintaining soil pH are thus an important management practice, in which application of lime (to raise the pH of acidic soil), S (to lower the pH of alkaline soil), incorporating soil organic matter (to buffer the pH change), and crop rotation (to balance soil pH over time) are common.^64^

##### 2.2.2 Temperature Effect

Since temperature affects the electrochemical gradient and driving forces, an increase or decrease in temperature, especially with respect to the root zone temperature (RZT), changes the electrical potential difference in electrogenic pumping.^65,66^ Several studies have shown plant growth inhibition at low RZT, as the highly negative stem water potential inhibits the uptake of various nutrients by roots and above-ground transport of nutrients.^67,68^ Moreover, low soil temperature (<10 °C) interacts with plant amino acid concentrations, indirectly affecting plant growth.^69^ In contrast, high soil temperature (>25 °C) causes protein denaturation and reduced microbial activity, which reduces plant mineral absorption.^70^

#### 2.3 Acid Soils and Alkaline Soils

The acidity or alkalinity of soil depends on the charge of soil organic or clay matter. Soil acidity is a condition in which the soil pH is lower than neutral (pH 7.0) (Figure 5), while soil alkalinity is a condition in which the soil pH is higher than neutral. Soil acidity or alkalinity happens when a considerable amount of exchangeable acidic cations (such as H^+^, Al^3+^, and Fe^3+^ ions) or alkali cations (such as Ca^2+^, Mg^2+^, K^+^, Na^+^, and Zn^2+^) respectively, accumulate and protonate the OH groups attached to the Al, Si, and Fe at the edges of clay minerals and the −NH_2_, −OH, and −COOH functional groups in the soil organic matter.^48^ The known causes of soil acidity are rainfall and leaching of exchangeable basic cations, acidic parent material (such as granite bedrock is more acidic than limestone bedrock), organic matter decay (CO_2_ produced from decomposition forms carbonic acid with water), and nitrification (conversion of NH_4_^+^ to NO_3_^–^ involves the formation of proton). Moreover, the production of high-yield crops removes micronutrients and exchangeable basic cations from the soil, which also contributes to soil acidity. The known causes of soil alkalinity are low rainfall or reduced leaching of exchangeable basic cations, alkaline limestone parent material, and application of hard water with high mineral content during irrigation.

**Figure 5:**
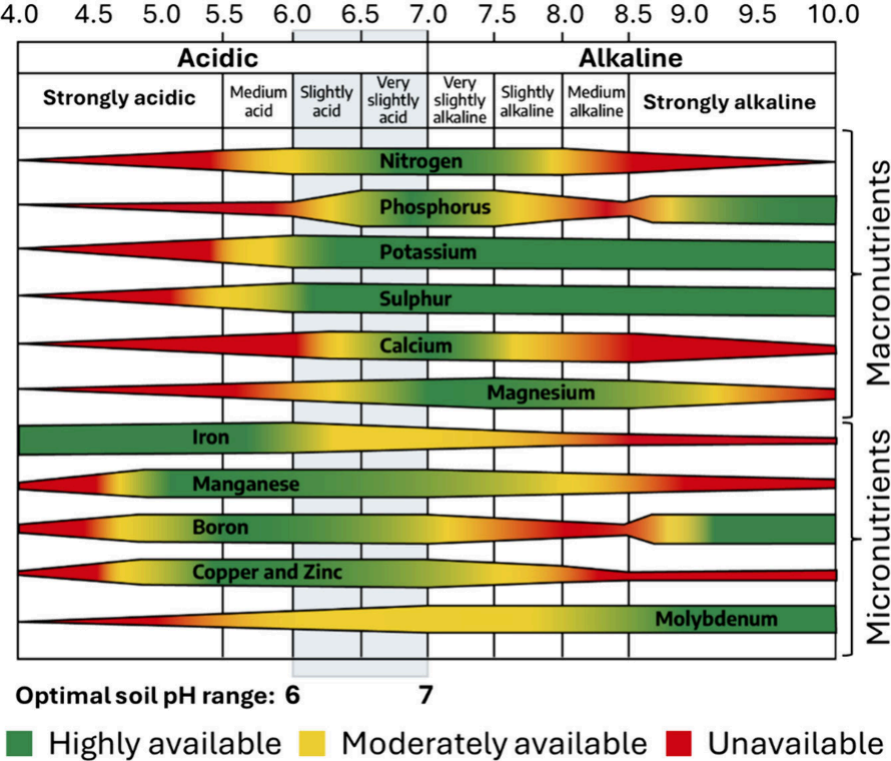
Changes in the availability of different minerals with soil pH.^49,50,71^ Wide bands represent the most-available minerals, whereas narrow bands represent limited mineral availability. Adapted with permission from Virginia Cooperative Extension^49^ Copyright 2023, Virginia Cooperative Extension, Virginia Polytechnic Institute and State University under CC BY-NC-SA 4.0 license (https://creativecommons.org/licenses/by-nc-sa/4.0/).

##### 2.3.1 Mineral Speciation, Complexation, and Availability

Mineral speciation, complexation, and availability largely depend on soil pH. Figure 5 illustrates a qualitative representation of the changes in mineral availability with respect to soil pH.^50^ In general, soil cation exchange capacity (CEC) decreases with soil pH and increases as the pH increases due to the availability of more base-forming cations. At a low pH, the solubility of Al and Mn in soil water decreases the exchangeable Ca and Mg levels, which reduces the Ca level in the plant available form. Meanwhile, the levels of Ca and Mg increase with an increase in soil pH. At pH >6, cations retained on soil clay minerals get exchanged with the soil solution, and plants remove these nutrients from the soil solution. This phenomenon is responsible for high nutrient losses through leaching from sandy soils with low CEC, whereas these nutrients are much less susceptible to losses from clay soils.

##### 2.3.1.1 Nitrogen

Nitrogen (N) is an essential macronutrient for plants; however, it is typically the most limiting component because of the low N content of soil. The total N content of most soils ranges between 200 to 4,000 ppm.^72^ A large portion of the total N is constituted in organic forms (90 wt %), whereas only about 1–4% is mineralized as plant-available N.^73^ It has been reported that 10–78% of added N is typically lost.^74,75^ Nitrogen is present in oxidation states ranging from +5 (as nitrate, NO_3_^–^) to −3 (as ammonium ion, NH_4_^+^). Nitrogen in the soil exists as different ionic species, and it is in equilibrium with atmospheric N_2_ and O_2_.^72^ Soil pH plays an important role affecting N availability to plants. The two plant-available N forms (NH_4_^+^ and NO_3_^–^) are pH-dependent; at high pH, NH_4_^+^ becomes ammonia and can be volatilized from the soil matrix. At low pH, additional H^+^ ions can help maintain the NH_4_^+^ form and can be absorbed by the plants. At high pH, NO_3_^–^ converts to N_2_ and reduces the NO_3_^–^ uptake by the plants.^76^

##### 2.3.1.2 Phosphorus

Phosphorus (P) is another important plant macronutrient after N. The total P content in soils ranges from 200 to 5,000 ppm, averaging 600 ppm.^77^ Phosphorus exists in soil mainly as H_2_PO_4_^–^ and HPO_4_^2–^, with p*K*_a_’s of 2.2 and 7.2, respectively. The availability of P depends on two competing factors: (i) reactions involving cations more abundant than P that may control P solubility, and (ii) reactions involving cations less abundant than P that may not control P solubility.^77^ The optimal P availability occurs between pH 6–7.^78^ Below pH 6, P forms insoluble minerals with Al/Fe/Mn, whereas above pH 7, P becomes an insoluble mineral with Ca. This phenomenon is related to the issue of lower P availability from the Ca–P fertilizers (triple superphosphate) in alkaline soils but not in acid soils.^79^

##### 2.3.1.3 Potassium

Potassium (K) is the most abundant nutrient mineral in soils, with the total K content ranging from 1,500 to 50,000 ppm with an average of 8,300 ppm.^80^ Of the total K content, 98% of K is present in the mineral form, whereas 2% is in the soil solution and exchangeable phases.^80,81^ Unlike N and P, whose chemical forms change with soil pH and other environmental factors, K in soils mostly exists in equilibria with the exchangeable, fixed, and mineral states. Although K is available in soil over a wide range of pH, as depicted in Figure 5, soil K exists in four forms: solution, exchangeable, fixed, and structural or mineral (in clay). The movement and solubility of K in soils depend on several factors, such as soil pH, CEC, and the rate of K application.^82,83^ Soils with higher pH and CEC can retain more K in the soil solution for plant uptake, whereas lower pH and CEC cause K leaching loss from the soil.^84^ Moreover, soil acidity increases the amount of Al and Mn in soil solution, which also inversely affects K uptake by plants.^85^

##### 2.3.1.4 Calcium and Magnesium

Calcium (Ca) and magnesium (Mg) contents are highly variable in soils, which is partly related to the soil parent material and rainfall. The total Ca content in soil ranges from 7,000–500,000 ppm, with an average value of 13,700 ppm.^86^ The total Mg content in soil ranges from 600–6,000 ppm, with an average value of 5,000 ppm.^87^ As depicted in Figure 5, the solubilities of Ca and Mg in soil water increase beyond pH 5.5. In soils, the concentrations of Ca and Mg are positively correlated with soil pH and CEC.^88^

##### 2.3.1.5 Silica

Silicon (Si) is the second most abundant element in soils, and shares structural similarities with carbon, but it is not considered an essential plant nutrient because plants can complete their life cycle without it.^89^ Silicon content in soil ranges from 1 to 45% by weight, with an average of 25–33% by weight. Unlike most nutrients, Si availability depends on interactions among soil pools and is strongly influenced by soil pH.^90^ Higher pH increases Si availability (Figure 6), whereas in acidic soils, Si precipitates with aluminum and iron sesquioxides, reducing plant availability. The solubility of silica (SiO_2_) expressed as neutral H_4_SiO_4_^0^, ranges from about 10^–2.74^ M for amorphous SiO_2_ to 10^–3.10^ M for quartz, with other SiO_2_ minerals showing intermediate solubilities. The polymerization, dissociation, and relative solubilities of other SiO_2_ species in soils are shown in Table 2.^91^ When soil pH increases from 1 to 10, the activities of two dominant species of silicon, H_3_SiO_4_^–^ and H_2_SiO_4_^2–^, increase, whereas the H_4_SiO_4_^0^ remains at equilibrium at approximately 10^–3.10^ M. H_3_SiO_4_^–^ increases from 10^–12.81^ M at pH 1 to 10^–2.81^ M at pH 10, while H_2_SiO_4_^2–^, increases from 10^–24.08^ M at pH 1 to 10^–6.08^ M at pH 10. It is apparent that in alkaline soils at pH above 9, H_3_SiO_4_^–^ and H_2_SiO_4_^2–^ ionic silicates significantly contribute to the total dissolved silicon. Nevertheless, Si accumulation in plants, particularly in the form of phytoliths, plays an important role in alleviating environmental stress. Silicon contributes to cell wall rigidity,^92^ facilitates efficient nutrient transport within the plant, and enhances resistance to pathogens and diseases.^93^ Silicon transport in plants is therefore an endogenous process that can improve nutrient-use efficiency and maximize crop yield, especially in Si-accumulating species such as rice, wheat, corn, soybean, and many other grains, fruits, and vegetables.

**Table 2:** Polymerization, Dissociation, and Relative Solubilities of SiO_2_ Species in Soils.^91^

Dissociation/polymerization reaction	Equilibrium constant	Equation for the activity of Si species
1 ${\mathrm{SiO}}_{2}\left(\mathrm{soil}\right)+H_{4}{{\mathrm{SiO}}_{4}}^{0}↔2H_{2}O$	log K^0^ = −3.10	$\log\left(a_{H_{4}SiO_{4}^{0}}\right)=-3.10$ (1)
2 ${\mathrm{SiO}}_{2}\left(\mathrm{soil}\right)+2H_{2}O↔H_{3}{{\mathrm{SiO}}_{4}}^{-}+H^{+}$	log K^0^ = −12.81	$\log\left(a_{H_{3}SiO_{4}^{-1}}\right)=pH-12.81$ (2)
3 ${\mathrm{SiO}}_{2}\left(\mathrm{soil}\right)+2H_{2}O↔H_{2}{{{\mathrm{SiO}}_{4}}^{2}}^{-}+2H^{+}$	log K^0^ = −26.08	$\log\left(a_{H_{2}SiO_{4}^{-2}}\right)=2pH-26.08$ (3)

**Figure 6:**
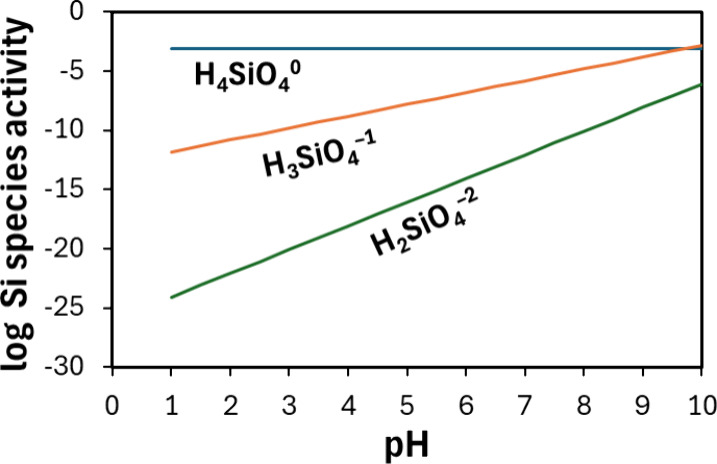
Silicon species activity as a function of pH. This figure shows that, with increasing pH, the activities of two dominant species of silicon, H_3_SiO_4_^–^ and H_2_SiO_4_^2–^, increase, whereas the H_4_SiO_4_^0^ remains at equilibrium at approximately 10^–3.10^ M (eq 1). It is apparent that for pH >9, H_3_SiO_4_^–^ and H_2_SiO_4_^2–^ ionic silicates significantly contribute to the total dissolved silicon. Values in Table 2 were adopted from Lindsay W.,^91^ and the figure was generated using eqs 1–3.

#### 2.4 Mechanisms of Mineral Absorption by Plants

Plant roots absorb different macro- and micronutrients in ionic form for developing and maintaining health.^53–56^ A plant needs 14 essential macro and micronutrients for its development and several beneficial nutrients to maintain its health.^94^ Plant roots absorb these nutrients/ions from soil throughout their life. When the nutrient is in a sufficient range in the soil to meet the plant demands, normal plant growth with maximum yield is expected. In contrast, lower concentration causes nutrient deficiency, whereas nutrient availability above sufficiency can cause nutrient toxicity.^95,96^

##### 2.4.1 Nutrient Transport from Soil to Root

There are three mechanisms through which soil nutrients reach plant roots: (i) water transport, (ii) ion diffusion, and (iii) root interception with a possible cation exchange.^94,97,98^ The movement of nutrients from soil solution to plant roots is mainly driven by transpiration-based water transport. The nutrient concentration in soil water varies with several soil factors. Thus, nutrient movement within plants significantly depends on water flow to the root. For corn, most nutrients are transported by this mechanism, specifically N, Ca, Mg, and S.^99^ The diffusion process depends on the concentration gradient of the ion present in the soil solution; by this process, the nutrients move slowly but steadily to the root zone. In the diffusion process, ions move from a high-concentration soil solution to the low-concentration root area, and a constant uptake of the nutrients by plant roots maintains a low concentration of ions at the root surface.^100,101^ In soil solution, nutrients like P and K are available at low concentrations and preferentially transported to roots through diffusion. The root interception process is not a significant mechanism for nutrient transport because only 1–2% of the soil surface is occupied by roots.^97^ The movement of nutrients by this mechanism depends on the growth of new roots in the soil, increasing the contact exchange of the nutrients. This process is also enhanced by mycorrhizal association because a higher root mass increases the nutrient acquisition from the soil by plant roots.^94^ These transport mechanisms are selective to plants because soil solution may contain high concentrations of mineral ions; however, plants are not required to absorb all these ions available in soil solution.

### Section Summary

In this section, we have discussed the role that soil components, such as organic matter, inorganic matter, and water, play in minerals management. While organic matter and inorganic matter are essential for supplying macronutrients and micronutrients, water plays a crucial role in transferring and delivering the stored nutrients to the plants. We also discussed soil and environmental factors that affect mineral uptake by plants. Temperature affects the electrochemical gradient and driving forces, therefore affecting mineral uptake and plant growth. Soil acidity is another factor that strongly influences mineral absorption, since it determines their solubility in water and availability. The presence of H^+^ or OH^–^ ions influences the exchange of anions (such as NO_3_^–^, PO_4_^3–^, and SO_4_^2–^) or cations (such as Ca^2+^, Mg^2+^, K^+^, and NH_4_^+^), respectively. For example, pH >7.5 decreases the availability of Ca, Mg, Fe, Mn, Zn, and P. We also discussed some macronutrient ionic species that are more readily available for plant uptake, such as NH_4_^+^ and NO_3_^–^, H_2_PO_4_^–^ and HPO_4_^2–^, as well as solubilized K, Ca, and Mg, and how they are transported from soil to plant roots.

## 3 Inorganic Composition of Biomass

### Section Overview

We now summarize the C, H, N, O, S, and Cl compositions of diverse biomass sources, as well as the main inorganic elements and ash content of lignocellulosic biomasses of interest for chemical and biofuel production. We focus on terrestrial biomasses such as woody biomass, agricultural and processing residues, and herbaceous biomass, with some aquatic biomass compositions for comparison. For example, the average ash content follows an increasing order of woody biomass (2.1 wt %), herbaceous biomass (4.5 wt %), agricultural residues (8.7 wt %), and aquatic biomasses (21 wt %). Elements such as N, S, Cl, P, and inorganics (i.e., K, Ca, Mg, Na, and SiO_2_) pose significant challenges during biomass conversion and catalytic upgrading in biorefineries. Herein, we present the typical range of concentration of each inorganic element in the different biomasses, with Si, K, and Ca being the dominant ones among the metals, and N, Cl, and S among nonmetal elements. Additionally, we discuss how minerals in biomass are classified into naturally occurring minerals and introduced minerals, and where these minerals are located within the plant anatomy.

#### 3.1 Biomass Feedstocks for Renewable Fuels and Chemicals

A summary of the main inorganic elements of some selected plants is given in Tables 3, 4, and 5.

**Table 3:** Inorganic Composition of Plant Biomass: Woody Biomass. Adapted from Giudiciani et al.^102^

Biomass	Ash	C	H	O	N	S	Cl	P	K	Ca	Mg	Na	Al	Fe	Si	Zn	Mn	Ref.
	**wt %**				**ppm**													
Ash wood	2.3	47	5.6	44.4	5,681	1,713	–	1,566	7,026	6,801	184	3,928	1232	285	–	–	–	^103^
Aspen bark	4.1	–	–	–	–	520	40	663	4,730	11,700	1,370	12	20	27	94	–	256	^8^
Aspen leaves	7.5	–	–	–	–	2,560	511	5,140	24,000	9,800	2,940	9	20	56	133	–	667	^8^
Aspen twigs	3.9	–	–	–	–	479	87	708	5,870	10,400	644	19	35	35	188	–	183	^8,9^
Aspen wood	0.4	48	6.2	45.6	2,000	112	35	191–320	1,390	998–4,200	286–490	16	8	5–30	63	–	49	^103,104^
Beech wood	0.4	–	–	–	–	–	–	–	796	584	222	77	–	–	–	–	–	^105^
Birch bark	2.5–2.7	–	–	–	–	329	149	428	710–1,710	6,960–7,860	323–635	14–115	19–118	24–238	114–204	155	534	^8,9,106^
Birch leaves	5.2	–	–	–	–	1,690	181	3,140	9,420	9,120	2,030	32	40	83	318	–	1,600	^8,9^
Birch twigs	2	–	–	–	–	493	120	820	3,020	4,730	448	43	23	40	69	–	354	^8,9^
Birch wood	0.3	–	–	–	–	82	40	49	315	636	92	4	2	6	77	–	102	^8,9^
Black locust wood	1.8	48.1	4.74	45.4	–	–	–	–	1,200	850	170	–	–	–	–	–		^107^
Black poplar	1.9–2.6	47.6–48.8	5.5–6	43.1–44.1	2,300	79–1,156	119	38–1150	1,597–3,523	2,554–8,642	276–852	9–2400	3–1,040	302	526	22	7	^103,108,109^
Douglas fir wood	0.3	–	–	–	–	–	–	–	464	572	80	–	10	23	–	4	21	^110^
Eucalyptus	0.4–2.7	46.8	5.4	45	–	956	–	1,720	250–5,709	2,000–14,250	212–250	11140	1,142	280	–	–	–	^103,111^
Loblolly pine bark	2.3	52.5	5.9	40.5	3,500	352	–	197	1,166	1,835	575	24	869	1,169	1,268	–	78	^112^
Moso bamboo	3.4	–	–	–	–	370	–	197	1,000	346	188	15	–	60	–	–	–	^113^
Olive tree pruning	12	40.7	5.7	41	5,900	700	300	650	3,000	11,000	850	72	1,000	1,300	36,000	15	26	^114^
Pine bark	1.1–2.4	–	–	–	–	311	147	1,260	663–3,180	1,118–6,350	169–874	22–130	241–908	52–3,015	60–80	20	343	^8,9^
Pine needles	2.5	–	–	–	–	845	407	1,270	4,770	4,140	804	28	374	40	549	–	839	^8,9^
Pine shoots	3	–	–	–	–	1,250	538	2,590	8,790	2,370	1,020	36	331	113	747	–	193	^8,9^
Pine twigs	2.1	–	–	–	–	587	200	848	3,040	5,300	715	40	332	73	312	–	244	^8,9^
Pine wood	0.2–1	49.8–50.4	6–6.7	43.1–43.7	950	63–702	85	41–147	137–590	384–1,800	92–400	5–60	4–71	7.5–82	123–150	63	28–101	^104,115–119^
Poplar wood	2–4.3	46.7	6.2	47	–	200	–	434	1,200–2,225	2,750–3,250	449–500	36–1000	–	24	42	20	6	^120,121^
Softwood sawdust	0.2–3.5	50.7	6.1	39.4–43	1,000–3,000	–	–	–	242–1,520	349–2,170	52–429	690	5–2,080	8–986	9,310	–	42	^122,123^
Spruce bark	2.4–3.1	–	–	–	–	367	260	452	1,120–2,030	7,693–8,350	657–865	26–64	92–98	39–61	169–3,602	61	714	^6,8,9,106^
Spruce needles	5.2	–	–	–	–	704	504	1,540	4,270	8,030	1,050	48	83	45	6,640	–	1,390	^8,9^
Spruce shoots	4	–	–	–	–	1,320	1,090	3,830	14,600	1,670	907	13	27	43	300	–	245	^8,9^
Spruce stump	0.4	–	–	–	–	–	–	–	245	1,235	–	36	–	–	253	–		^6^
Spruce twigs	2.3	–	–	–	–	776	317	1,080	3,560	4,320	909	97	221	167	982	–	496	^8,9^
Spruce wood	0.3	–	–	–	–	50	51	4	215–272	720–1,030	95	6–22	2.2	3.8	59–82	–	98–100	^104,124^
Willow	1.3–5.1	46.7–49.6	5.3–6	42–46	3,000–5,269	301–1,341	–	467–1870	1,610–8,057	3,133–12,820	321–385	58–3,698	60–6,646	31–987	725	50	17–32	^103,125–127^
**Average**	2.3	48.4	5.9	43.6	3,025	652	249	1,014	2,822	4,111	542	494	462	252	1,983	46	284	
***Min–Max***	*0.2–12*	*40.7–52.5*	*4.7–6.7*	*39.4–47*	*900–5,900*	*50–2,560*	*35–1,090*	*4–5,140*	*137–24,000*	*346–14,250*	*52–2,940*	*4–11,140*	*2–6,646*	*4–3,015*	*42–36,000*	*4–155*	*6–1,600*	

**Table 4:** Inorganic Composition of Plant Biomass: Agricultural Residues. Adapted from Giudiciani et al.^102^

Biomass	Ash	C	H	O	N	S	Cl	P	K	Ca	Mg	Na	Al	Fe	Si	Zn	Mn	Ref.
	**wt %**				**ppm**													
Almond shell	0.7	–	–	–	–	288	–	383	6,714	1,570	500	165	–	1,641	–	10	30	^128^
Barley straw	–	–	–	–	–	492	3,700–4,100	753	8,600–10,757	3,441–4,300	1,205	661	64	54	2,900–4,600	9	16	^109^
Coconut coir	0.9	47.6	5.7	45.6	2,000	64	–	<1	22	4	5	16	1	2	27	–	–	^129^
Coconut shell	0.7	50.2	5.7	43.4	–	35	–	<1	14	11	3	9	<1	1	2	–	–	^129^
Coffee husk	3.6	46.4	6.3	44.5	2,660	900	–	1,000	12,500	5,000	1,300	–	–	176	–	11	24	^130^
Coir pith	7.1	44	4.7	43.4	7,000	467	–	84	1,866	222	575	750	117	59	927	–	–	^129^
Corn cob	2.8	45.8–47.6	5–6.2	44.6	2,000	15	–	12	262–7,958	5–3,827	47–1,023	4–180	–	1	276	–	–	^131^
Corn stalks	6.8–8.2	41.9	5.3	46	–	564	–	145	2–10,000	319–4,000	403–2,500	439	130	35–85	911	10	–	^129^
Corn stover	10.5	44.5	5.4	39.5	8,000	2,567	3,000	2,761	23,097	11,670	5,175	6,434	7,333	3,808	36,239	–	–	^132^
Corn straw	8.8	43	4.7	42.1	8,200	6,000	13,636	–	28,435	3,718	2,130	6,970	–	–	–	–	–	^133^
Cotton stalks	5.5	45.2	6.3	42.4	6,700	–	–	–	9,300	8,700	2,700	800	900	1,000	–	–	–	^134,135^
Millet husk	18.1	42.7	6	33	1,000	317	–	229	699	1,132	2,016	258		185	27,302	–	–	^129^
Nut shell	5.9	48.3	5.7	39.4	8,000	299	–	16	1,044	765	209	28	215	64	647	–	–	^129^
Palm empty fruit bunch	6.9	–	–	–	–	374	–	666	22,600	1,900	1,400	280	467	1,800	5,100	–	54	^136^
Palm kernel shell	3.3	–	–	–	–	92	–	218	2,400	2,600	1,900	104	233	1,300	4,000	–	31	^136^
Palm mesocarp fiber	6.3	–	–	–	–	534	–	937	9,600	2,800	1,800	133	393	4,200	9,300	–	113	^136^
Pine nut shell	1.3	–	–	–	–	652	–	716	2,201	728	905	193	–	1,318	–	28	59	^128^
Potato plant stems	9.6–11.7	–	–	–	–	–	–	–	50,320–82,930	5,768–15,590	324–3,999	175–339	–	–	–	–	–	^105^
Rape straw	6.0	46.3	5.46	42.2			–	–	18,600	5,030	1,580	720	–	–	300	–	–	^6^
Rice husk	16.5–23.5	37.8–39.4	4.7–5.1	32–38.6	3,000–6,000	163–600	480–500	79–291	2,129–3,000	421–895	87–379	15–62	15	17–125	21,375–51,862	–	–	^129,137,138^
Rice straw	10.6–19.8	36.9–41.2	4.9–7	34.2–37.9	4,000–12,300	221–2,700	6,487–9,990	149–1,455	1,070–27,148	945–6,410	1,244–5,467	334–3,866	–	41–461	34,553	–	–	^129,139–141^
Rye straw	–	–	–	–	–	–	2,400	–	9,700	2,700	–	–	–	–	3,500	–	–	^29^
Soybean stalks	3.4	–	–	–	–	–	–	–	5,500	7,000	1,500	–	–	25	–	5	–	^142^
Sugar cane Bagasse	1–6.9	43.8–49.7	5.8–6.1	43.8–48.8	2,000–7,300	60–8,450	270	8–1,075	78–4,976	44–950	182–1,040	3–666	119–279	4–327	503–8,600	12	52	^116,129,143–146^
Sugar cane trash	5.3–7.6	40.1–49.5	5.3–6.1	44–48.8	3,100–5,000	800	1,760	–	2,300–5,250	3,680	492–1,850	57–430	216	245	13,400	–	–	^143,144^
Walnut shell	0.7	–	–	–	–	790	–	691	5,202	9,081	1,283	554	–	3,095	–	18	111	^128^
Wheat straw	4.1–11.2	44.9–49.4	4.23–6.2	35.8–48.8	1,000–6,000	409–1,000	1,000	20–1,700	451–16,900	103–5,332	31–1,093	7–2,365	6–1,100	8–1,100	349–12,400	6–100	27–100	^109,116,127,129,137,147^
**Average**	8.4	44.1	5.7	42	5,115	1,106	4,655	522	13,991	3,639	1,376	808	628	678	10,190	21	56	
***Min***–***Max***	*0.7–23.5*	*36.9–50.2*	*4.2–7*	*32–48.8*	*1,000–12,300*	*15–8,450*	*270–13,636*	*1–2,761*	*2–82,930*	*4–15,590*	*3–5,467*	*3–6,970*	*1–7,333*	*1–4,200*	*2–51,862*	*5–100*	*16–113*	

**Table 5:** Inorganic Composition of Plant Biomass: Herbaceous Biomass and Aquatic Biomass. Adapted from Giudiciani et al.^102^

Biomass	Ash	C	H	O	N	S	Cl	P	K	Ca	Mg	Na	Al	Fe	Si	Zn	Mn	Ref.
	**wt %**				**ppm**													
**Herbaceous biomass**																		
Giant reed (*Arundo donax*)	3.9–5.1	43–45.5	5.8–6.1	44.4–45.8	3,500	900	–	663–900	10,610	444	245–500	108	–	–	–	–	–	^148^
Hay	6.0	47.6	6.2	45.1	11,000	–	–	–	28,826	1,795	1,361	737	–	–	–	–	–	^116^
Miscanthus	–	47.6	6.3	–	–	1,584	1,232–1,232	–	4,300–11,551	1,200–4,400	–	40	–	–	–	–	90	^47^
Reed grass	2.4–6.8	44.6–49.5	5.4–6.1	40.4–48.5	3,400–13,900	1,150–2,000	300	652–1,510	1,456–10,695	1,014–2,795	125–627	74–87	106–396	114–276	4,062	60	240	^125^
Switchgrass	1.5–4.3	44.8–46.8	5.6–5.8	45.8–49.1	2,000–4,000	296–615	–	494–1,117	717–4,398	1,360–6,173	542–1,644	24–158	23–102	39–113	2,903–5,123	20	41–60	^127^
Thistle	6.3	40.7	5.7	46.2	11,900	1,100	10,000	1,100	9,600	14,000	1,900	4,400	930	640	7,700	11	30	^114^
**Average**	4.5	45.4	5.9	45.9	6,200	1,026	3,844	927	7,408	3,430	820	546	265	205	4,947	30	92	
***Min***–***Max***	*1.5–6.8*	*40.7–49.5*	*5.4–6.3*	*40.4–49.1*	*2,000–13,900*	*296–2,000*	*300–10,000*	*494–1510*	*717–28,826*	*400–14,000*	*125–1,900*	*24–4,400*	*23–930*	*39–640*	*2,903–7,700*	*11–60*	*30–240*	
**Aquatic biomass**																		
Brown macroalgae Sargassum	27.5	–	–	–	–	11,600	–	400	8,000	–	21,870	1800	–	414	1,700	73	53	^149^
Freshwater microalgae	16.8	39.9	5.8	31.4	61,000	0	–	–	9,230	67,450	5,590	5560	480	780	–	–	560	^150^
Marine microalgae	29.9	34.8	5.3	24.6	54,000	0	–	–	10,266	27,688	2,633	50,321	789	477	–	–	128	^150^
Azolla	8.7					3,240	–	6,500	5,180	9,520	4,300	16,000	638	1,470	4,100	37	206	^151^
Other aquatic plants^a^	7.6–64.4	25.2–56.4	2.7–8.7	28.5–39.2	22,600–76,000	1,200–69,000	3,320–6,140	–	–	–	–	–	–	–	–	–	–	^152,153^
**Average**	21.2	40.2	5.3	32.4	43,475	14,015	4,445	3,450	8,169	34,886	8,598	18,420	636	785	2,900	55	237	
***Min***–***Max***	*7.6–64.4*	*25.2–56.4*	*2.7–8.7*	*24.6–39.2*	*22,600–76,000*	*0–69,000*	*3,320–6,140*	*400–6,500*	*5,180–10,266*	*9,520–67,450*	*2,633–21,870*	*1,800–50,321*	*480–789*	*414–1,470*	*1,700–4,100*	*37–73*	*53–560*	

a Other aquatic plants include Eichornia crassipes (water hyacinth), Hydrilla verticillata, Lemna minor, Spyrogyra sp., Lyngbya sp., Cladophora sp., P. stratiotes, N. Lotus, sea lettuce, chlorella, spirulina, Tetraselmis sp., Derbesia tenuissima, and Oedogonium sp.

As summarized in Tables 3–5, biomass has C (35–53 wt %), O (25–49 wt %), and H (4–7 wt %) as its major constituent elements, with a high dependence on the biomass type and the ash content. This elemental composition agrees with data reported by Vassilev et al.^10^ As discussed in Section 2.2, plants need other essential elements classified as macronutrients (N, S, P, K, Ca, and Mg), micronutrients (Fe, Zn, Mn, Cu, Mo, B, Ni, and Cl), and other essential elements (e.g., Na and Si). As shown in Figure 7, most of these elements in lignocellulosic biomass have concentrations below 15,000 ppm (1.5 wt %). Ca can have concentrations up to 15,000 ppm in the three types of biomasses (woody, herbaceous, and agricultural residues). K can be up to 15,000 ppm in woody and herbaceous biomass, with higher concentrations in agricultural residues, up to 28,400 ppm, with an exceptional case (potato plant stems) up to 83,000 ppm. In woody biomass, Si concentrations are typically below 2,000 ppm, with some cases up to 9,300 ppm (softwood sawdust), and an exceptional case (olive tree pruning) up to 36,000 ppm. In herbaceous biomass, Si concentrations are between 2,900 and 7,700 ppm. In agricultural residues, the Si concentration is highly variable, typically below 2,700 ppm, with some exceptional cases up to 51,800 ppm (rice husk). Na is less than 4,000 ppm in woody biomass, less than 700 ppm in herbaceous biomass, and less than 7,000 ppm in agricultural residues. Mg content in woody biomass and herbaceous biomass is typically below 2,000 ppm and can be up to 5,000 ppm in agricultural residues. In the three types of biomasses, Al could be present up to 1,000 ppm in average, Fe up to 5,000 ppm, and Mn and Zn mostly below 100 ppm, with some woody biomasses having up to 1,600 ppm of Mn.

**Figure 7:**
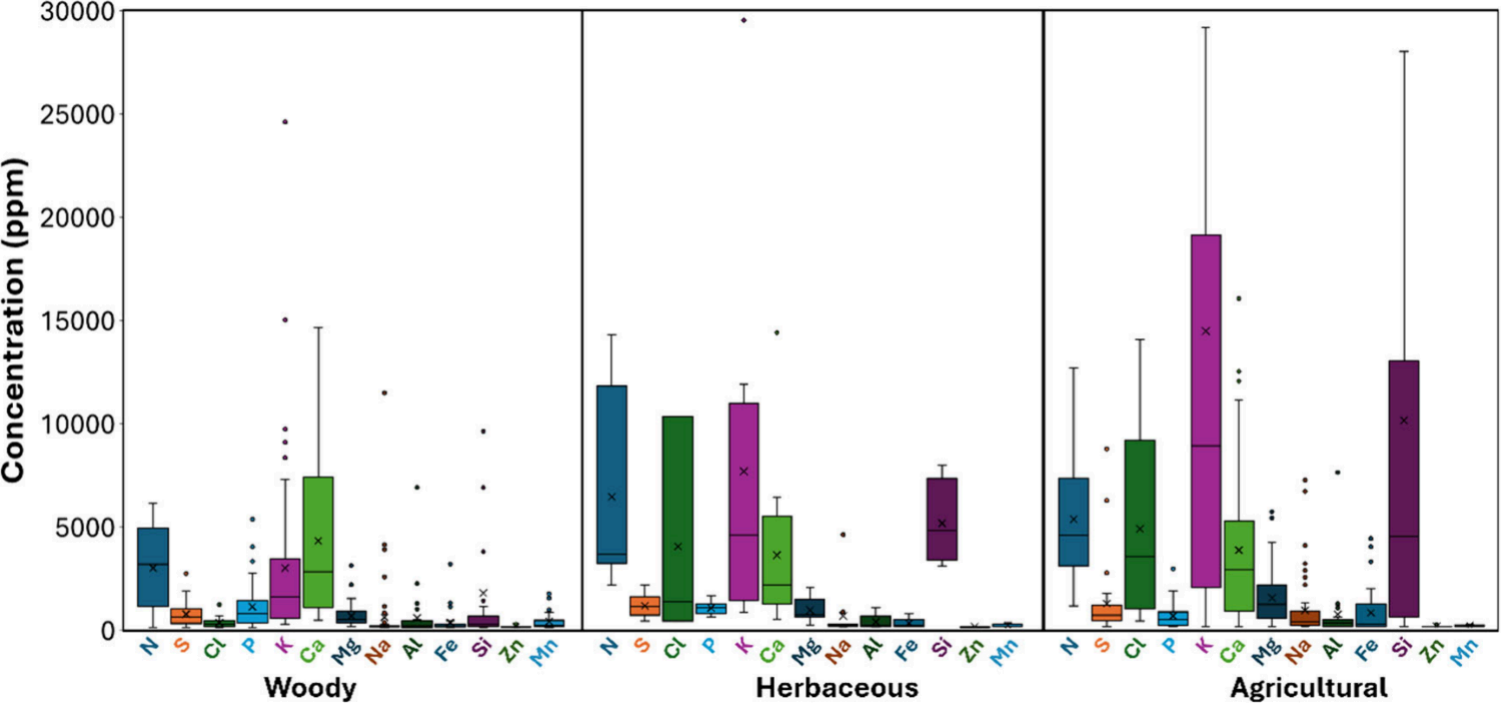
Mean (**x**) concentration of most relevant nonmetal and metallic elements in woody biomass, herbaceous biomass, and agricultural residues. Each bar displays the minimum and maximum (whiskers), the median (middle horizontal line), and the range (box) corresponding to 50% of data points for each biomass. Dots indicate outlier data points. The analysis included 122 biomass samples for a total of 1,007 data points from the references summarized in Tables 3–5

Nonmetal elements such as N can be present in woody biomass up to 6,000 ppm, up to 14,000 ppm in herbaceous biomass, and up to 12,300 ppm in agricultural residues. While Cl content in woody biomass is below 1,000 ppm, it is up to 10,000 ppm in herbaceous biomass and 13,600 ppm in agricultural residues. S and P concentrations are commonly below 2,000 ppm, with some exceptions such as up to 8,000 ppm of S in agricultural residues and up to 5,000 ppm of P in woody biomass. Figure 7 compares the average content of inorganic elements in each type of biomass, with the dominant ones being Si, K, and Ca among the metals and N, Cl, and S among nonmetal elements.

Figure 8 summarizes the average ash content in the four different types of biomasses detailed in Tables 3–5. Woody biomass has the lowest ash content, ranging from 0.2 to 5.1 wt %, with an average of 2.1 wt % (except for olive tree pruning, which has 12 wt % of ashes). Herbaceous biomass has an average ash content of 4.5 wt %, ranging from 1.5 to 6.8 wt %. Agricultural residues (straw, husk, stalks, shells, and others) have the highest ash content from terrestrial lignocellulosic biomasses, with an average of 8.7 wt %, ranging from 0.7 to 23.5 wt %. Rice straw and husks are the residues with the highest ash content, ranging from 10 to 23.5 wt %, followed by potato plant stems (9.6–11.7 wt %), corn residues (7–11 wt %), and wheat residues (4–11 wt %). Aquatic biomasses have higher ash content compared to terrestrial lignocellulosic biomasses, ranging from 7.6 to 42 wt %, with high contributions of Ca, Na, Mg, K, N, and S.

**Figure 8:**
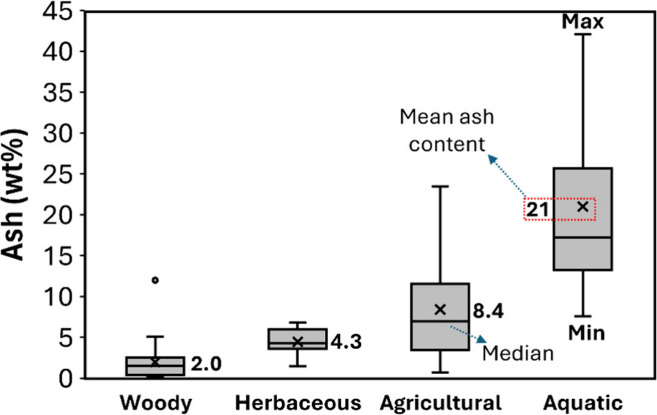
Mean (**x**) ash content in woody, herbaceous, agricultural residues, and aquatic biomass. Each chart displays the minimum and maximum (whiskers), the median (middle horizontal line), and the range (box) corresponding to 50% of data points for each biomass. Dots in charts indicate outlier data points. The analysis included 132 data points from the references summarized in Tables 3–5 and additional references 151–153 for aquatic biomasses.

##### 3.1.1 Inorganic Content in Woody Biomass, Herbaceous Biomass, and Agricultural Residues

Woody biomass includes materials derived from coniferous (softwoods) and deciduous (hardwoods) trees, such as stems, branches, bark, sawdust, wood chips, pellets, and briquettes. The high lignin and cellulose content in wood makes it a preferred choice for bioenergy production and bioproducts because of its low inorganic content.^154^ Woody biomass, particularly forestry residues, contains a variety of inorganics that influence its combustion properties and environmental impact. The inorganic content varies depending on the wood species and the fraction of the tree.^155^ According to Table 3, the chemical composition of woody biomass ranges from 40.7–52.5 wt % C, 39.4–47 wt % O, and 4.7–6.7 wt % H. It also contains 900–5,900 ppm of N, up to 2,560 ppm of S, up to 5,140 ppm of P, and up to 1,090 ppm of Cl. K, Ca, and Si are the inorganics with the highest concentrations, ranging from 137–24,000 ppm, 346–14,250 ppm, and 42–36,000 ppm, respectively. Na and Mg have concentrations up to 11,140 ppm and 2,940 ppm, respectively, while Al, Fe, Mn, and Zn have concentrations up to 6,646, 3,015, 1,600, and 155 ppm, respectively. The ash content in woody biomass ranges from 0.2 to 5.1 wt %. Bark comprises the external part of a tree that surrounds the vascular cambium and is exposed to ambient contaminants, such as soil, during plant growth and harvesting, and tends to have a higher inorganic content than wood.^13,106^ As shown in Table 3, the mean ash content of aspen, birch, and spruce bark is 4.1, 2.6, and 2.7 wt %, respectively. Aspen, birch, and spruce wood have only 0.4, 0.3, and 0.3 wt % of ash, respectively.

Herbaceous plants are a group of plants that have leaves and stems that die at the end of the growing season. These plants do not contain wood. Their structure is composed of more loosely bound fibers of hemicellulose, cellulose, and lignin. This means that the lignin content of herbaceous plants is lower than that of woody plants.^156^ The composition of herbaceous biomass (Table 5) ranges from 40.7–49.9 wt % C, 40.4–49.1 wt % O, and 5.4–6.3 wt % H as the main components. Herbaceous biomass also contains 2,000–13,900 ppm of N, 296–2,000 ppm of S, 494–1,510 ppm of P, and 494–10,000 ppm of Cl. K, Ca, and Si are the main inorganic components with concentrations ranging from 717 to 28,826 ppm, 400 to 14,000 ppm, and 2,903 to 7,700 ppm, respectively, followed by Na and Mg with concentrations of 24–4,400 ppm and 125–1,900 ppm, respectively. Al, Fe, Mn, and Zn have lower concentrations, up to 930, 640, 240, and 60 ppm, respectively. The ash content in herbaceous biomass is between 1.5 and 6.8 wt %.

Processing residues are generated from various agricultural and industrial activities and often contain significant inorganic content, as summarized in Table 4.^102^ The typical chemical composition of agricultural residues ranges from 36.9–50.2 wt % C, 32–42.8 wt % O, and 4.2–7 wt % H. They also include 1,000–12,300 ppm of N, up to 8,450 ppm of S, up to 2,761 ppm of P, and up to 13,636 ppm of Cl. Typical dominant inorganics in agricultural crop residues include K, Ca, and Si with concentrations up to 82,930 ppm, 15,590 ppm and 51,862 ppm, respectively. Na, Al, Mg, and Fe, can be present up to 6,970, 7,333, 5,467, and 4,200 ppm, respectively, while Mn and Zn have concentrations below 113 ppm.

Other elements such as Ni^109,118,132,145^ and Ti^114,125,137^ can be found in biomass in concentrations up to 450 and 50 ppm, respectively, while some others (Cu,^109,118,126,128,130,142,145,147^ Mo,^126^ Ba,^110,120,145^ Co,^118,145^ Cr,^118,145^ Pb,^145^ Sr,^145^ Cd,^118^ Li,^118^ and V^118^) can be present in concentrations below 20 ppm, and are not commonly analyzed or reported. Algae biomass can have higher concentrations of some of these elements; for example, algae can contain Cu and Cr at levels of up to 100 and 150 ppm, respectively.^149,150^

Compared to terrestrial lignocellulosic biomasses, aquatic biomasses have lower C (25.2–56.4 wt %) and O (24.6–39.2 wt %) concentrations, comparable H (2.7–8.7 wt %) concentrations and higher N and S concentrations, ranging from 22,600 to 76,000 ppm and up to 69,000 ppm, respectively (see Table 5). Among the inorganic content in aquatic biomasses, Na is highest with concentrations up to 50,000 ppm, followed by Ca (up to 67,000 ppm), K (up to 10,266 ppm), and Mg (up to 21,870 ppm). Si content can be up to 4,000 ppm, and other inorganic elements such as Al, Fe, Zn, and Mn have concentrations below 1,500 ppm. The ash content is highly variable, ranging from 7.6 to 42 wt % with an average of 21 wt % (Figure 8).

#### 3.2 Minerals Classification and Locations in Plants

Plant minerals can be differentiated into two groups: a) naturally occurring minerals (physiological or inherent or biogenic minerals or intrinsic) that are absorbed during plant growth and b) introduced minerals (anthropogenic or technogenic or extrinsic or external minerals) that adhere to soil particles and the plant surface during harvesting, handling, transport, and processing, especially in crops like corn stover, wheat straw, and sugar cane bagasse.^157^ Intrinsic minerals can further be differentiated into structural minerals (plant-cell wall-associated minerals) and vascular minerals (plant-cell extract-associated minerals).^158^ Intrinsic minerals are distributed in different anatomical parts of the plants—integrated into the cell wall as part of the physical support structure, inside cell walls, and contained in water transport elements. The origins of transition metals/post-transition metals in the plants are primarily technogenic, such as during biomass handling and harvest, but they can also be naturally taken up by the roots.^158^

A significant amount of inorganic metals in biomass comprises essential nutrients for plant growth, such as K, Mg, and Ca, and will remain in the harvested plants. Alkali and alkaline earth metals are present in plants in large amounts, especially in fast-growing plants, and can constitute more than 80% of inorganic material in biomass. The mineral content of herbaceous biomass, such as grass, is higher than that of wood due to its rapid growth, which results in the uptake of more nutrients during its development.^159^

Within plant structures, metal ion complexation mechanisms vary depending on both the plants and the individual metal ions. Plant minerals in biomass are classified into four different groups: (i) dissolved salts and other inorganic components such as K^+^, Na^+^, Cl^–^, and SO_4_^2–^ that precipitate when biomass loses water during the postharvest naturally drying process, (ii) organically bound minerals such as K^+^, Na^+^, Mg^2+^, and Fe^2+^, found in organic components of the biomass, (iii) minerals found within the biomass matrix, such as SiO_2_, CaC_2_O_4_, Fe_2_(SO_4_)_3_, and FePO_4_, precipitated via natural processes in the biomass, and (iv) external minerals that are attached to the biomass from soils and other foreign particles, such as sand, clay minerals, and metallic aluminum.^9,157,160^

Inorganic elements are essential for numerous physiological functions in the plant. N, P, and S play a crucial role as constituents of organic compounds such as amino acids (cysteine, methionine), vitamins (thiamine, biotin), proteins, chlorophylls, nucleic acids, phospholipids, and phosphate esters of sugars. Fe, Ca, Mg, Mn, Mo, Zn, and Cu act as enzyme activators in cytochromes, peroxidases, catalases, the hydrolysis of ATP and phospholipids, carbohydrate metabolism, the synthesis of DNA and RNA, nitrite and nitrate reductase, etc. K^+^ and Na^+^ help to increase membrane permeability, while Ca^2+^ and Mg^2+^ reduce permeability. Ca can be present as calcium pectate in cell walls, and Mg as a component of chlorophyll molecules and pectate in the middle lamellae.^158^

The locations of inorganics in plants are tissue-type dependent (roots, leaves, stems, fruit, and seeds). As explained in Section 2.2, the root system is the primary site for mineral absorption with specialized structures that enhance nutrient uptake, including root hairs that increase surface area.^161^ The nutrients absorbed here are transported to other parts of the plant via the xylem. Leaves contain chlorophyll and other compounds that require inorganic elements for photosynthesis and metabolic functions. For instance, Mg is a central complexing metal of chlorophyll.^161^ Stems serve as conduits for transporting water and nutrients between roots and leaves. They also store some inorganics temporarily.^161^ Fruits accumulate inorganics (Ca, P, Mg, Zn, and P) that are essential for seed development and germination. For example, P and N are critical for energy transfer and biosynthesis during seed germination.^162^ The inorganic content of fruits and seeds can vary depending on the type of fruit or seed.

Silicon in biomass can be found as insoluble amorphous silica (SiO_2_), accounting for around 90 wt %, silicate ions (0.5–8 wt %), and colloidal acid (0–3.3 wt %). Amorphous silica can be found in epidermal cells of plants in dumbbell shaped cells called opal phytolith or silica phytolith.^163–166^ The shape and size of silica phytoliths can be highly variable depending on the biomass source and, therefore, on the specific cell and intercellular spaces characteristic of each plant species.^167,168^ Studies carried out using petrographic microscope, XRD, SEM, and TEM have shown that these silica phytoliths can be round with a diameter of 10–30 μm, oblong with 18–40 μm length and 12–18 μm width, or globular with a size of 12–16 μm. In some cases, the silica particles could be larger with sizes up to 200 μm.^164–166,169–171^ Additionally, Si can interact with plant cell wall components through covalent bonds (C–O–Si) and form organosilicon complex layers. Si can interact at different degrees with cellulose, callose (β-(1,3) linked glucan), structural proteins, hemicellulose, pectin, and lignin, mainly through hydroxyl groups and possibly through the phenolic acid of a ligno-carbohydrate complex.^172^

Silicon plays multiple roles in plant health and development. It improves plant resistance to lodging and drought, strengthens defenses against diseases, insects, and nematodes, and improves tolerance to toxic metals. Additionally, silicon enhances soil nutrient availability and nutrient balance within the plant, mitigating the harmful effects of stressful conditions on growth.^167,168,172–179^ For example, Si establishes a P buffer system, increasing levels of organic phosphoesters under P deficiency and limiting their availability under excess P, thereby reducing P toxicity. Si also helps to immobilize toxic metal ions (Al, As, Cd, Fe, Mn, and Zn) via (i) complexation and removing them from the rhizosphere as insoluble precipitates, (ii) enhancing Mn, Fe, Cd, and Zn deposition in the cell wall (as Si-wall matrix-metal cocomplex) and reducing their uptake in the cytoplasm, and (iii) forming hydroxyl-aluminosilicate nanostructures in the cell wall, intercellular spaces, and vessel elements, such as the hydrophilic surface of the cell membrane, cellulose polysaccharide hydroxyl system of the cell wall, and various hydrophilic groups of glycoprotein.^168,172,176–179^

### Section Summary

In this section, we have summarized the typical concentration ranges of the main organic and inorganic elements that compose lignocellulosic biomasses, including C, H, O, N, S, P, Cl, and inorganic elements such as K, Ca, Mg, Na, and Si. K, Ca, and Si are the dominant (>10,000 ppm) contributors to biomass ashes, followed by Mg, Na, and Al (<10,000 ppm), Fe (<5,000 ppm), and Mn and Zn (<100 ppm). Nonmetal elements such as N (<15,000 ppm), Cl (<10,000 ppm), S, and P (<5,000 ppm) are also important components in lignocellulosic biomasses. Agricultural residues have the highest (8.4 wt %) average ash content, followed by herbaceous biomass (4.3 wt %), and woody biomass (2 wt %). Additionally, we discussed the classification of inorganics as naturally occurring and externally introduced minerals, as well as their locations within the plant anatomy. Minerals can be classified into four different groups: (i) dissolved salts and other inorganic (K^+^, Na^+^, Cl^–^, and SO_4_^2–^) components that precipitate during biomass drying, (ii) organically bound minerals (K^+^, Na^+^, Mg^2+^, and Fe^2+^) found in organic components of the biomass, (iii) precipitated minerals such as SiO_2_, CaC_2_O_4_, Fe_2_(SO_4_)_3_, and FePO_4_ found within the biomass matrix, and (iv) external minerals that are attached to the biomass from soils and other foreign particles.

## 4 Influence of Inorganics in Thermal Biomass Reactions

### Section Overview

In this section, we describe different noncatalytic thermal approaches to convert biomass, including combustion, gasification, pyrolysis, and hydrothermal liquefaction (HTL). We then discuss the role of the inorganics in each of these thermochemical processes, and the properties and upgrading needs for pyrolysis- and HTL-derived bio-oil that typically needs to go through a catalytic deoxygenation process to improve its quality for end-use applications. Inorganics in biomass can cause wear, ash or char agglomeration, deposits, slagging, fouling, and corrosion of equipment in thermochemical processes, especially in combustion, pyrolysis, and gasification. We discuss the catalytic effect of inorganics during gasification, especially their influence on tar formation and elimination. We also analyze the challenges posed by other elements, such as S, N, and Cl, during gasification and the need to remove them from the biomass feedstock before conversion processes to avoid negative effects on equipment and downstream applications of the syngas product. Similarly, we discuss the catalytic effects of inorganics during catalytic fast pyrolysis and hydrothermal processing. Finally, we discuss how around 10% of the inorganics in biomass are transferred to bio-oil during pyrolysis, while in HTL and HTC (hydrothermal carbonization), almost 90% of the inorganics in biomass are transferred to the liquid phase product.

#### 4.1 Combustion and Ash-Related Issues

Biomass can be used for heat and power generation, contributing between 10%–15% of the global energy demand with woody biomass, agricultural residues, and municipal solid waste as feedstocks.^180–182^ Combustion involves heat production as a result of the oxidation of carbon- and hydrogen-rich biomass to CO_2_ and H_2_O; incomplete combustion generates other byproducts, such as CH_4_, CO, and particulate matter.^183^ Biomass combustion generates ash as the major residue, which is classified as fly ash and bottom ash. Ash can reduce combustion system efficiency, increase cleaning and maintenance costs and shutdown times.^180,184^ The fly ashes are mainly formed by fine ash particles that are separated from the stream of gases outside the combustion chamber by means of specially designed systems like cyclones, while bottom ashes are produced in the combustion chamber and formed by totally or partially burnt coarse particles, mainly silicon oxide mixed with other inorganic impurities contained in the biomass.^184^

Using biomass as a fuel in the combustion process can cause agglomeration, deposits, slagging, and fouling of the equipment, as shown in Figure 9. Typically, alkali metals such as K and Na interact with silica, aluminosilicates, S, Cl, and carbonates during combustion, forming alkali silicates, sulfates, chlorides, and carbonates that deposit on heat transfer and combustion chamber surfaces. Detailed discussions of ash-related issues in combustion and ash formation mechanisms are described by Niu et al.^185–188^ Deposits on combustion chambers can be comprised of alkali metals and alkaline earth metals in the form of chlorides, sulfates, carbonates, and complex silicates. For example, K can form KCl, K_2_SO_4_, K_2_CO_4_, and K_2_CO_3_.^185,188^ Slagging is a specific type of deposit formation when K, Na, and SiO_2_ react in the presence of Cl at temperatures higher than 800 °C. Fouling is described as the formation of undesired deposits on heat transfer surfaces, causing a reduction in heat transfer rates, higher resistance to heat exchanger fluid flow, and associated pressure drop that reflects a reduction in overall heat exchanger efficiency and useful life.^163^ Additionally, agglomeration is caused by the presence of alkali and alkaline metals that reduces the bottom ash fusion temperature, producing fused ashes that adhere to the combustor wall material. These ashes cause temperature gradients along the ash bed, reduced biomass fluidization, and reduced efficiency in power generation.^163^

**Figure 9:**
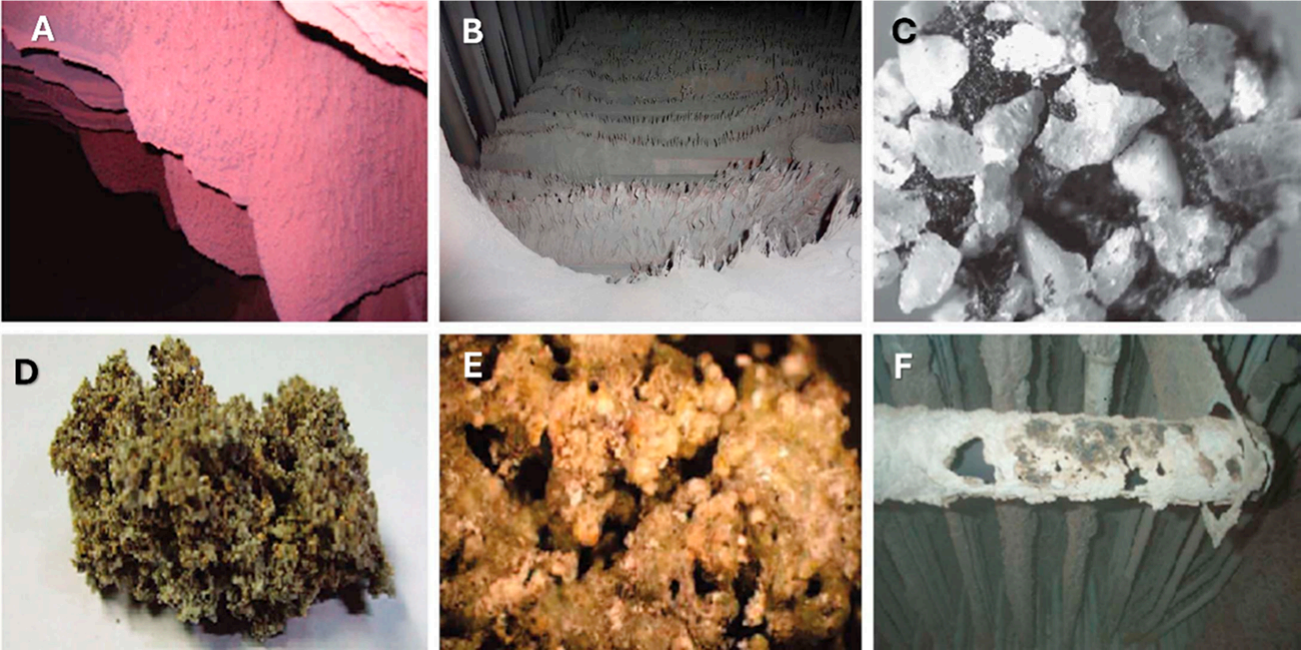
Examples of representative technical problems resulting from the presence of inorganics during biomass combustion. Alkali-induced slagging in A) a 12 megawatt (MW) furnace, and B) a commercial fluidized bed. C) Agglomeration in a lab-scale bubbling fluidized bed. Silicate melt-induced slagging (ash fusion) in a D) 12 MW furnace, and E) a lab-scale furnace. F) Corrosion in a commercial bubbling fluidized bed. Reprinted with permission from Niu et al.^185^ Copyright 2016, Elsevier.

#### 4.2 Gasification and the Role of Inorganics in Tar Formation and Nitrogen Mitigation

Biomass gasification involves the conversion of solid or liquid organic materials at high temperatures (700–1,200 °C) in the presence of an oxidant as coreactant (air, O_2_, steam, CO_2,_ or their mixtures) to produce mainly either synthesis gas (CO + H_2_) or producer gas (CO + H_2_ + N_2_). Byproducts include condensable hydrocarbon (mostly liquid) tar as well as solid products such as carbonaceous residue (char).^189–192^ The major product, syngas, is a mixture of carbon monoxide (CO), hydrogen (H_2_), methane (CH_4_), and carbon dioxide (CO_2_), along with small amounts of light hydrocarbons, such as ethane (C_2_H_6_) and propane (C_3_H_8_). In addition, undesirable gases such as nitrogen compounds [ammonia (NH_3_) and hydrogen cyanide (HCN)], S-containing inorganic compounds [hydrogen sulfide (H_2_S), carbonyl sulfide (COS), and carbon disulfide (CS_2_)], halogen compounds (HCl and Cl), and alkali (Na, K) and heavy metals are also produced. The composition depends on the biomass feedstock, the gasification technology, and the operating conditions employed.^31,193^ The syngas produced has a low heating value (LHV) range of 4–13 MJ·Nm^–3^, which varies based on feedstock quality, reactor type, and process conditions. Syngas is a versatile energy carrier that can be utilized for power generation, biofuel synthesis, or as a feedstock for chemical production.

One significant challenge in biomass gasification is the formation of high molecular weight condensable hydrocarbons, which are collectively referred to as tar. Tar formation poses technical and economic challenges to biomass gasification, as it necessitates additional cleaning and upgrading of the product gas to ensure its suitability for downstream applications.^193^ These challenges have hindered the industrial viability of biomass gasification and have led to the cancellation of several large gasification projects.^194,195^ To address these issues, this section examines the definition and classification of tar, its associated challenges in gasification processes, its quantification methods, and the critical role of biomass inorganics in the formation and elimination of tar. Understanding these interactions is essential for optimizing gasification performance and improving the feasibility of syngas as a clean energy source. The role of inorganics in biomass is particularly crucial because metals such as alkali and alkaline earth metals (AAEMs, like K, Na, Ca, and Mg), along with other elements (e.g., Si, Al) inherently present in the feedstock, can act as natural catalysts or inhibitors during gasification. These inorganics influence the thermal decomposition of biomass components, affecting the release of volatiles and the subsequent formation of tar through pathways like cracking, polymerization, and reforming reactions. For instance, AAEMs can promote the breakdown of heavier hydrocarbons into lighter gases, potentially reducing tar yields, while interactions with silica may form inactive silicates that hinder this catalytic effect. This interplay underscores the need to examine tar management strategies in the context of biomass composition.

Beyond tar-related challenges, contaminants such as S, N, and Cl pose additional technical concerns during gasification.^196^ Particularly, nitrogen-containing species, primarily derived from biomass proteins, can be converted mainly into nitrogen oxides (NO_*x*_), an environmental pollutant, which may further react to form nitric acid (HNO_3_), leading to corrosion of downstream equipment. This issue was notably observed in the recent shutdown of Fulcrum BioEnergy industrial-scale gasification facility in Nevada, USA.^197^ Effective nitrogen mitigation and management strategies are therefore essential to minimize NO_*x*_ formation, minimize equipment degradation, and ensure the long-term reliability of biomass gasification systems.

##### 4.2.1 Gasification Tar Management

##### 4.2.1.1 Definition, Classification, and Challenges of Biomass Gasification Tar

Tar refers to a complex heterogeneous mixture of condensable hydrocarbons. Owing to the wide range of institutions and researchers involved in biomass gasification studies, numerous definitions and sampling methodologies for tar have been proposed. The Gasification Taskforce of the IEA Bioenergy Agreement, the U.S. Department of Energy (DOE), and DGXVII of the European Commission reached a consensus defining tar as hydrocarbons with molecular weights higher than benzene.^198^ In essence, tar is a highly viscous, brown-to-black liquid composed of single-ring to polycyclic aromatic hydrocarbons (PAHs), oxygenated hydrocarbons, and other complex polycyclic compounds.^199,200^

For a better description of tar, the Energy Research Center of The Netherlands (ECN) established a clear classification methodology to divide tar into five distinct classes based on molecular weight and condensation behavior, as shown in Table 6. These classes provide a useful framework for understanding tar-related fouling phenomena and evaluating gas cleaning system performance. While the total quantity of tar in the syngas is important, the composition and properties of the tar compounds ultimately dictate their behavior, particularly in terms of condensation and deposition.

**Table 6:** List of Tar Classifications.^193,194,200,201^

Tar class	Class name	Property	Representative compounds
1	Gas chromatography (GC) undetectable	Very heavy tars, cannot be detected by GC	Determined by subtracting the GC-detectable tar fraction from the total gravimetric tar
2	Heterocyclic aromatics	Tars containing hetero atoms and are highly water-soluble	Pyridine, phenol, cresols, quinoline, dibenzophenol
3	Light aromatics	Single-ring water-soluble light hydrocarbons with low condensability	Toluene, ethylbenzene, xylenes, styrene
4	Light PAH compounds	2- and 3-ring compounds can easily condense at low temperatures	Indene, naphthalene, biphenyl, fluorene, phenanthrene, anthracene
5	Heavy PAH compounds	Larger than 3-ring compounds (4–7) that condense at high temperatures with low concentrations	Fluoranthene, pyrene, chrysene, perylene, coronene

Overcoming tar-related challenges is essential for the proper operation of biomass gasification technologies.^202–204^ Tar formation not only wastes 5–15% of the effective energy from biomass gasification but also reduces process efficiency and leads to reactor shutdown.^205^ The high molecular weight hydrocarbons that constitute tar (Table 6) are undesirable byproducts that pose significant technical and economic barriers in biomass gasification. Tar tends to polymerize into more complex and refractory structures when encountering low temperatures in processing units, leading to operational issues such as fouling, clogging, and corrosion in downstream equipment, including pipelines, heat exchangers, and filters. Consequently, these issues result in increased maintenance, higher emissions, reduced energy recovery, and escalating operational costs of gasification systems. Clearly, effective tar removal or cracking methods—whether catalytic, thermal, or physical—are required to ensure smooth operation and improved syngas quality.

Tar directly affects the suitability of the product gas for downstream applications. Given the diversity of end-use applications for syngas, ranging from power generation and biofuel synthesis to feedstock for methanol production, effective tar management is critical. Gas quality requirements vary widely across these applications, with limits on tar content dictated by the specific technology employed, as shown in Table 7. For example, in direct combustion systems, where the product gas is burned while still hot, tar condensation is minimal and there are generally no restrictions on tar levels. However, in systems such as internal combustion engines, gas turbines, and fuel cells, stricter tar limits apply due to the sensitivity of these technologies to contaminants. Internal combustion engines, for instance, can tolerate some impurities, but tar condensation within fuel injection systems can cause operational failures. Gas turbines are more sensitive, as tar and particulate matter can be deposited on turbine blades, reducing efficiency and causing damage under high-temperature operation. For syngas applications in biorefining, like methanol synthesis^212^ or Fischer–Trøpsch synthesis,^213^ tar can poison catalysts. Additionally, higher tar loads require more robust gas cleaning systems, which add to the capital and operating costs of gasification plants.

**Table 7:** Tar Concentration Threshold in Syngas for Various Applications.

Application	Tar concentration (mg·Nm^–3^)	Ref.
Direct combustion	No specified limits	
Compressors	50–500	^206^
Internal combustion engine	<100	^207^
Gas turbine	<5	^208^
Fuel cells	<1	^209^
Methanol synthesis	<0.1	^210^
Fischer–Trøpsch synthesis	<0.1	^211^

In summary, tar remains one of the major limitations hindering the widespread adoption of biomass gasification. Its propensity to condense, polymerize, and deposit on critical components reduces efficiency, increases maintenance costs, and restricts the applicability of syngas in advanced energy systems. Addressing these challenges through innovative tar cracking and avoidance technologies will be key to unlocking the full potential of biomass gasification as a sustainable energy solution.

##### 4.2.1.2 Tar Content Determination and Characterization in Biomass Gasification

Accurate tar measurement is essential for optimizing gas cleanup and ensuring effective biomass conversion. Given the variability in feedstocks, operating conditions, and reactor configurations, robust measurement methods are crucial for both research and industrial applications.^196,214,215^ Two main approaches are used for tar measurement in biomass gasification: off-line methods and online methods.^216^ Off-line techniques, such as the tar protocol, solid-phase adsorption (SPA), and solid-phase microextraction (SPME), involve sample extraction and laboratory analysis^195^ as summarized in Table 8. The tar protocol remains the standard for total tar quantification, while SPA and SPME offer faster sampling with lower solvent use. Despite their detailed compositional insights via gas chromatography (GC) and mass spectrometry (MS), off-line methods are time-intensive and unsuitable for real-time monitoring, limiting their industrial applicability.

**Table 8:** Features of off-Line Tar Measurement Methods.^195,217–219^

	Tar protocol	Solid phase adsorption (SPA)	Solid phase microextraction (SPME)
Principle	Cold trapping in liquid solvent (isopropanol)	Adsorption in a solid phase (e.g., amine-based, activated carbon)	Adsorption in a solid stationary phase (silica fiber with PDMS)
Sampling time	1–2 h	1–2 min	10–20 min
Desorption time	n.a.^a^	1 h	n.a.
Analysis time	1 h	1 h	1 h
Advantages	• Robust method	• Easy and fast sampling	• Easy and rather fast sampling
	• Measures the total tar	• Small amounts of solvent	• Solvent-free sampling
		• No loss of tar adsorbed by solvent evaporation and aerosol formation	• Suitable for measuring very low tar amounts
		• High accuracy and reproducibility	
Limitation	• Long sampling times	• Only measures GC-detectable compounds	• Under development
	• Bulky and complicated sampling	• Inadequate for heavy tar	• Application in raw product gas is uncertain
	• Large solvent volumes	• BTX must be analyzed within a few hours to avoid its desorption	• Only measures GC-detectable compounds
	• Loss of adsorbed tar by solvent evaporation and aerosol formation		• Nonaromatic C5+ hydrocarbons might compete with tar compounds for the adsorption sites
	• Inadequate for low tar concentration		No standard procedure is available
	• Low precision		
Detection limit	5 mg·Nm^–3^	2.5 mg·Nm^–3^	0.1 mg·Nm^–3^

a n.a.= not applicable.

Online tar measurement methods allow real-time monitoring of tar content directly in the syngas stream, providing immediate feedback for process control and optimization.^220^ Technologies such as flame ionization detectors (FID),^221^ photoionization detectors (PID),^222^ optical spectroscopy (e.g., laser-induced fluorescence and UV-LED fluorescence), and mass spectrometry (e.g., molecular beam MS and ion–molecule reaction MS) are among the most prominent online methods.^223^ Online techniques are faster and more practical for industrial applications, where rapid adjustments to operating conditions are critical for maintaining syngas quality and equipment reliability. However, these methods face high costs, equipment fouling, and limited sensitivity when measuring complex tar mixtures.^224–226^

##### 4.2.1.3 Roles of Inorganics in Tar Formation

The presence of inorganics,^11^ such as K, Na, Ca, Mg, Si, and Al in biomass, plays an important role in influencing the gasification process, particularly in the formation and reduction of tar. These inorganics can act as natural catalysts during biomass gasification,^227,228^ promoting the decomposition of heavier tar molecules, enhancing gas yields, and improving process efficiency.^199,229,230^ However, the influence of inorganics on tar formation and decomposition is complex as it depends on the type of biomass feedstock, the chemical form of the inorganics, and the specific operating conditions of the gasification process. While inorganics can play a catalytic role in promoting tar decomposition, under certain conditions they may also hinder the process.

The catalytic effects of inorganics on product distribution during biomass gasification have been widely reported, with some studies reporting the effect of inorganics on tar formation. For example, Guo et al.^133^ reported that the inorganics present in corn stover promoted the production of methane (CH_4_) and light hydrocarbons (C_*x*_H_*y*_) at moderate gasification temperatures (600–800 °C). At temperatures above 750 °C, the inorganics catalyzed gas-phase reactions, leading to an increase in H_2_ and CO yields while simultaneously reducing tar content. Yoo et al.^231^ investigated air gasification of empty fruit bunches (EFB) with and without inorganics removal. They observed that leaching inorganics with tap water and 0.1% nitric acid wash from EFB reduced the ash content from 5.9% to 1.7% and 2.0%. The demineralized EFB increased H_2_ and CO yields by 5% and 1–2%, respectively, while reducing CO_2_ formation by 34.8%. The removal of inorganics also mitigated agglomeration and sintering during gasification from 8.7 wt % to 1.4 wt % with the nitric acid wash, enhancing overall process performance. However, Skoulou et al.^232^ observed that very high temperatures (above 950 °C) caused volatilization of alkali metals, diminishing their catalytic activity and limiting their effectiveness in tar steam reforming.

The role of inorganics in tar formation is further demonstrated through comparative studies with demineralized biomass.^233,234^ Mitsuoka et al.^235^ studied the CO_2_ gasification of woody (Japanese cypress) biomass char and the effects of K and Ca. The presence of K and Ca compounds in char improved the reactivity of Japanese cypress char for CO_2_ gasification catalytically. They also confirmed that K and Ca compounds can be supported on char to exhibit an enhanced catalytic effect during CO_2_ gasification of K-char and Ca-char. The gasification rate of acid-washed char was very slow compared to the nonwashed and the impregnated char. Therefore, the catalytic roles of biomass-inherent minerals in tar formation and reduction during biomass gasification cannot be overlooked. As such, the complete removal of inorganics from biomass for gasification purposes may not be desirable and needs to be carefully analyzed depending on process conditions and reactor types.

According to Jiang et al.,^233^ inorganics play a role in the transformation of tar species and thereby their reduction. The presence of minerals, especially metallic species of AAEMs, catalyzes the thermal cracking of tar at the primary gasification stage of biomass decomposition and leads to the partial decomposition of Class 2 tar (heterocyclic aromatics) into lighter hydrocarbons (Class 3) or intermediate PAHs (Class 4). Simultaneously, AAEMs promote the catalytic cracking of larger PAHs (Class 5) into smaller aromatics or syngas components and the repolymerization of macromolecules to form char and reduce tar yield.^236^ Similar results were also reported by Zhang et al.,^237^ who demonstrated the role of potassium-rich char in promoting char formation and tar reduction during the gasification of woody and agricultural waste.

Despite the positive catalytic effects of AAEMs, their interactions with other inorganic species, particularly silicon, can limit their activity. In silica-rich biomass, such as rice husk, alkali metals react with silica to form alkali silicates, which are catalytically inactive under gasification conditions. Zhang et al.^238,239^ demonstrated that the formation of these silicates at lower temperatures decreased the catalytic activity of K and Na, reducing their effectiveness in tar reforming and gasification activity.

As outlined above, inorganics such as K, Na, Ca, Mg, Al, and Si play a critical role in the formation, decomposition, and transformation of tar during biomass gasification. The dual role of inorganics in promoting tar decomposition while influencing char formation and physicochemical aspects that affect char reactivity in gas-phase reactions have significant implications for biomass gasification. At moderate temperatures, inorganics can catalyze tar cracking, reduce tar yields, and enhance syngas quality. However, at higher temperatures, volatilization and mineral-silicon interactions reduce their effectiveness, posing challenges for process optimization. Future research must focus on understanding the individual contributions of these inorganics, optimizing their catalytic roles, and designing targeted pretreatment strategies. Such advancements are essential for improving the efficiency and cost-effectiveness of gasification systems in future biorefineries.

##### 4.2.2 Nitrogen and Challenges in Biomass Gasification

##### 4.2.2.1 Migration of Nitrogen during Biomass Gasification

Nitrogen in biomass is mainly contained in amino acids and proteins and is an important part of plant enzymes and structural molecules.^240^ During gasification, this fuel-bound nitrogen (fuel-N) is released and converted into various nitrogen-containing compounds depending on the process conditions. Fuel-N is typically divided into volatile nitrogen (volatile-N), which is released during volatilization, and char nitrogen (char-N), which remains in the solid and is released later during char conversion.^241–244^ The chemical structure of nitrogen-containing compounds in biomass, such as pyrrolic, pyridinic, or quaternary nitrogen, significantly dictates their release pathways and transformation products. The presence of minerals in biomass, such as AAEMs (K, Na, Ca, Mg) and iron, significantly influences these nitrogen transformations by catalyzing the decomposition of proteins and amino acids, promoting NH_3_ formation, and reducing HCN yields through hydrolysis reactions.^245,246^

Volatile-N includes ammonia (NH_3_), hydrogen cyanide (HCN), nitrogen oxides (NO, N_2_O), isocyanic acid (HNCO), nitrogen gas (N_2_), and tar-bound nitrogen (tar-N).^247^ The release and distribution of these species depend on temperature, heating rate, particle size, and the ash composition of the feedstock.^248^ In fluidized bed gasification, NH_3_ is typically the major gas-phase nitrogen compound, while HCN is formed in smaller amounts.^249^ Pyrolysis of biomass initially releases nitrogen as tar-N, which is later cracked into NH_3_ and HCN.^250–252^ The initial release of tar-N is particularly significant in high-protein biomass, where nitrogen is predominantly bound in complex heterocyclic structures, influencing the subsequent formation of gaseous nitrogen species.^253,254^

NH_3_ and HCN are widely recognized as the primary precursors of NO_*x*_ during biomass gasification and are released at different stages of the process.^255^ At the early stage, 30–80% of fuel-N is released as volatile-N through thermal decomposition.^256–258^ Recent simulations of glutamic acid pyrolysis and steam gasification, a model for protein-rich biomass, confirm that 62–70% of fuel-N converts to NH_3_ via deamination, with HCN yields being very low (<5%), particularly in high-volatile feedstocks.^246^ The amount of volatile-N released depends not only on the nitrogen content but also on the chemical structure of nitrogen compounds.^259^ For example, HCN is mainly released at temperatures above 500 °C due to the decomposition of nitrile-N and heterocyclic-N, while NH_3_ is produced from the ring-opening and hydrogenation of heterocyclic-N and the breakdown of amine-N.^260,261^ These transformations are highly sensitive to the nitrogen functional groups, which vary across feedstocks such as woody, herbaceous, or nitrogen-rich residues, including high-protein microalgae or sewage sludge.^262–264^

In the later gasification stage, steam injection promotes reforming of volatiles, leading to additional NH_3_ and HCN formation.^265^ While this stage is theoretically homogeneous, it often involves some contact with char, thus partly promoting heterogeneously catalyzed reactions.^245^ Char can influence these reactions by providing hydrogen radicals and catalytic minerals.^266^ As a result, char-N interactions, catalytic hydrolysis of volatile-N, and char-N gasification can occur simultaneously. However, the contribution of char-N to NH_3_ and HCN formation is generally small, particularly for feedstocks with high volatile and low fixed-carbon contents. For these feedstocks, char-N accounts for 10–30% of fuel-N but contributes only 1–15% of total NH_3_ and HCN yields, with HCN from char-N typically limited to 1–5% of fuel-N (e.g., 5–20 mg·Nm^–3^ or 4.5–18 ppm).^267,268^ Because HCN is highly toxic, these low yields are critical, as even small concentrations pose significant environmental and health risks, necessitating precise measurement and control strategies. Future research should focus on real-time monitoring of HCN emissions to better quantify its release under varying gasification conditions. To elucidate the complexity of nitrogen transformation, analyzing N-species behavior across gasification stages is essential; however, current studies remain limited, particularly for emerging nitrogen-rich biomass feedstocks.

##### 4.2.2.2 Effect of Inorganics and Pretreatment on NO_*x*_ Precursors

Minerals in biomass, especially AAEMs, play a key role in nitrogen transformation during gasification. These elements can catalyze reactions that control the release and conversion of nitrogen-containing species, affecting NO and NO_2_ formation and reduction. For instance, Ca and K can enhance NO reduction through heterogeneous reactions with char or CO, while also influencing the breakdown of NH_3_ and HCN.^269,270^ NH_3_ and HCN formed during devolatilization and tar cracking are influenced by inorganic content and gasification conditions. Studies show that CaO can reduce the yields of HCN, NH_3_ and HNCO, while iron minerals tend to suppress HCN and tar-N formation during devolatilization. These effects occur during the homogeneous stage, where minerals do not directly contact volatile species but still influence their reforming behavior.^245^ In the heterogeneous stage, minerals present in char or ash can directly catalyze reactions. For example, char-supported Fe shows high catalytic activity for the hydrolysis of HCN and the decomposition of NH_3_,^271^ while CaO in char can fix NH_3_ and convert it to N_2_.^272^ The catalytic activity of these minerals is highly dependent on their dispersion within the biomass matrix and their interaction with nitrogen functional groups, which warrants further investigation to optimize NO_*x*_ mitigation strategies.^273^

Pretreatment methods, such as torrefaction^247,274,275^ and water-leaching,^247,248^ which change the chemical composition of the biomass (discussed in Section 7), could affect NO_*x*_ emissions. This behavior results because the NO_*x*_ generated during gasification can react with the char surface, and these reactions are often catalyzed by ash minerals. However, only a limited number of studies^247,276^ have examined how pretreatment influences the release of fuel-N, and the mechanisms involved are still not well understood. Pretreatment of herbaceous biomass and the use of additives are commonly applied to reduce ash-related problems in fluidized bed gasification, including bed agglomeration, fouling, slagging, and high-temperature corrosion.^277^ Preliminary findings also suggest that pretreatments like water-leaching may enhance NH_3_ yields by reducing catalytic mineral content, but this could inadvertently increase NO_*x*_ precursor emissions, necessitating a balanced approach to biomass upgrading.^278^ These treatments can also influence the release of NO_*x*_ and its precursors, NH_3_, and HCN. Therefore, a clear understanding of nitrogen chemistry and its transformation pathways during gasification or combustion is essential for minimizing nitrogen-related environmental impacts.^279^

Ash-forming elements have a strong influence on nitrogen reactions, and several studies^280–283^ have shown that Ca and K can catalyze the reduction of NO by char. However, the interplay between ash composition, pretreatment, and nitrogen chemistry is complex, with potential trade-offs between reducing ash-related operational issues and managing NO_*x*_ emissions. Aznar et al.^284^ examined the catalytic roles of metal species (Ca, Na, K, and Fe) in municipal sludge on nitrogen migration during gasification. Their results indicated that individual metals, notably Ca and K, enhance N_2_ formation by catalyzing the reduction of NH_3_ and HCN. However, mixed ash compositions in sludge demonstrated only a marginal increase in N_2_ production due to intricate interactions among ash constituents.^284^ Despite these findings, current research on the interaction between ash composition, pretreatment, and nitrogen transformation remains limited. Further experimental and mechanistic studies are needed to fully understand these effects and develop effective strategies for NO_*x*_ and HCN mitigation in biomass gasification systems. Future research should explore how advanced pretreatment techniques, such as biochemical looping or additive-enhanced gasification, influence nitrogen speciation and NO_*x*_ precursor formation, particularly for nitrogen-rich biogenic residues.

#### 4.3 Fast Pyrolysis and Effect of Inorganics on Product Selectivities and Yields

The pyrolysis of lignocellulosic biomass is influenced by the presence of naturally occurring metals, particularly AAEMs. These metals alter the pyrolysis process by catalyzing different reactions (e.g., dehydration, dehydrogenation, decarbonylation, decarboxylation, deoxygenation, hydrolysis, and cracking)^285^ and changing the distribution and composition of pyrolytic products. The effect of these inorganics in biomass during the pyrolysis process depends on the specific chemical form, concentration, and interaction with the biomass structure, leading to variations in the yield and type of products generated during pyrolysis.

Calcium in the form of CaO can react with CO_2_ and acetic acid during pyrolysis, forming CaCO_3_, which increases the solid yield and decreases the oil yield.^285^ CaCl_2_, on the other hand, promotes the ring-opening reactions of pyran structures, leading to the generation of furan and linear aliphatics (e.g., acetic acid, 2-propanone, 3-penten-2-ona). Ca also promotes the formation of nitrogen-containing compounds during pyrolysis, reducing the activation energy and enhancing bio-oil quality.^286^

Like Ca, magnesium salts such as MgCl_2_ enhance the formation of furan and cycloketones by promoting the cyclization of fragmented radicals. Mg is more effective than Ca in promoting the generation of linear aliphatics and cyclopentanones. The presence of Ca and Mg can also weaken the bond connections between the structural components in biomass, facilitating the breakdown of hemicellulose and cellulose connections, which leads to increased sugar yields and decreased phenyl and CO_2_ yields.^285^

AAEMs catalyze different biomass degradation pathways, favoring carbohydrate degradation over lignin degradation due to their ability to cleave polysaccharide C–C bonds^287^ and influence the chemical composition of bio-oils. For example, Ca and K promote the formation of nitrogen-containing compounds and pyridines in nitrogen-rich pyrolysis of cellulose.^286^ Mg and Ca are particularly effective in catalyzing the breakdown of polysaccharides over lignin.^287,288^ K and Fe can also have similar catalytic effects. Potassium salts, for instance, catalyze ring scission reactions increasing carboxylic acid yields from 6.4% to 7.3% for untreated biomass and biomass with 2 wt % of KCl added.^287^ At higher temperatures (>380 °C), K promotes char formation and enhances the degradation of cellulose and hemicellulose.^289^ The catalytic action of iron-based compounds can reduce the average activation energy of lignocellulosic biomass waste from 189.9 to 141.8 kJ mol^–1^.^287,290^

The anions associated with metallic compounds, such as chlorides (CaCl_2_, MgCl_2_), and oxides (CaO, MgO), influence the pyrolysis process. Chlorides tend to create acidic conditions that favor C–C bond cleavage and carboxylation, while oxides have a more limited impact on the primary pyrolysis chemistry but can alter secondary reaction pathways.^285^

Lignocellulosic components decompose at different temperatures; for instance, cellulose decomposes between 291 and 395 °C, while hemicellulose decomposes between 173 and 230 °C, and lignin has a broader range from 170 to 835 °C.^291^ However, the presence of AAEMs can shift these temperature ranges, affecting the overall thermal behavior of biomass. Table 9 summarizes some important effects of AAEMs during biomass fast pyrolysis.

**Table 9:** Effects of AAEMs on Lignocellulosic Biomass Pyrolysis

	Alkali metals (e.g., K and Na)	Alkaline earth metals (e.g., Ca and Mg)
Catalytic effects	Lower the activation energy for biomass decomposition, promoting the formation of bio-oil and syngas.^292^	Promote the formation of linear aliphatic compounds and cyclopentanones, while inhibiting the formation of CO.^285^
Product distribution	Enhance the formation of low-molecular-weight compounds, such as CO and CO_2_, while suppressing the formation of phenols and other aromatic compounds.^293,294^	Enhance the formation of solid residues (biochar) at the expense of bio-oil yields.^285^
Catalyst deactivation	Contribute to catalyst deactivation by fouling or poisoning the catalyst surface.^294,295^	Contribute to catalyst deactivation by altering the structure and composition of the catalyst.^294^

Heavy metals, such as Zn, Pb, Cu, and Cd, also influence the quality and yield of bio-oil and biochar produced from the pyrolysis of lignocellulosic biomass. The presence of heavy metals in biomass poses environmental concerns, as these metals can be released into the environment during pyrolysis.^286,296^ Heavy metals are typically retained in the solid residue (biochar) and can lower the activation energy for biomass decomposition.^297^ Typically, more than 70% of the heavy metals accumulate in the biochar. Certain heavy metals can enhance the yield and quality of bio-oil. For instance, 1 wt % Cu impregnated over fir sawdust and pyrolyzed at 500 °C in a vertical drop fixed-bed reactor has been shown to improve the high heating value (HHV) of bio-oil from 11 to 14.8 MJ·kg^–1^. Cu also promoted the decomposition of lignin into small-molecular aromatic compounds, thereby increasing by 14.4% the fractions of C7–C10 compounds in the bio-oil, and increasing the bio-oil yield from 47.4% to 54.3% when the Cu content was 1 wt %.^298^

Removing the inorganics from the biomass prior to pyrolysis reduces the formation of undesirable byproducts, such as chars and low-value gases, and enhances the quality of bio-oil.^299^ High metal content in the pyrolysis oil can deactivate catalysts used in catalytic pyrolysis and foul process equipment.^295,300^ For instance, chemical pretreatments that remove inorganics have been shown to improve the calorific value of biomass and enhance the production of aromatic hydrocarbons, which are valuable components of bio-oil.^301^ Alkali metal salts, particularly carbonates, have been shown to facilitate the production of biochar and gases at the expense of bio-oil due to their catalytic effects, which promote the generation of gas products and increase the condensation degree of biochar.^302^ Pyrolysis experiments on cottonwood biomass also showed that tar yields increased markedly from 25% to 43% after inorganic removal through acid leaching, highlighting their role in suppressing tar formation.^303^

A study reported by Chandler et al.^304^ of the pyrolysis of unwashed and washed herbaceous biomass (*Arundo donax*) in a fluidized bed reactor showed that the biomass washed with water at 60 °C produced 9.7% more bio-oil than the unwashed biomass. The glycolaldehyde and levoglucosan yields increased by 0.8 and 1.2 wt %, respectively. Char was reduced by 4.3 wt %. Water washing of *Arundo donax* showed a 57% reduction in ash and an 86% reduction in K. Similarly, P, S, Mg, and Ca were reduced by 90%, 75%, 53%, and 25% in biomass, respectively.

Chen et al.^134^ reported that during the pyrolysis of cotton stalk, the inherent minerals contributed to a reduced bio-oil yield and a poor quality. By removing the minerals using the aqueous phase bio-oil to wash the cotton stalk, there was a decrease in water and acid content in the resulting bio-oil, along with an increase in phenol formation. The bio-oil obtained from the nonwashed cotton stalk contained 58.2 wt % water, while the bio-oil obtained from the washed biomass contained only 34.45 wt % water. The pretreatment of the raw biomass with an aqueous phase bio-oil reduced the metallic species and their catalytic effects on the dehydration reaction, thereby promoting the formation of pyrolysis intermediates into bio-oil. Also, the HHV of the bio-oil increased from 11.34 to 13.46 MJ·kg^–1^ for the raw and washed cotton stalk, respectively. The bio-oil yield remained almost unchanged, but a reduction of around 3–5% in char and noncondensable gases was observed when the biomass was washed to remove inorganics.^134^

Fahmi et al.^126,127^ investigated the effect of inorganic species in biomass (grasses) on pyrolysis oil yields, quality, and stability by using a pyrolyzer-GCMS, finding a strong catalytic effect of Na, K, Ca, and Mg, promoting an ionic route that favors ring-scission and hydroxyacetaldehyde formation. The washing treatment they applied reduced the metal content in the washed biomass to 60–80% of the inorganics in the raw biomass. They observed an increase in the intensities of levoglucosan, suggesting a ring scission mechanism of cellulose decomposition rather than the defragmentation of glucose monomers in cellulose, suggesting that inorganics in biomass promote a higher biomass degradation rate.^126^ They also reported a clear trend between the total liquid yield and the total ash/inorganics in the feedstock—the total liquid and organic yields increase as the total ash content decreases. For example, the total liquid yield was around 70 wt % for a 1 wt % ash content, and around 50 wt % of total liquid yield for an 8 wt % ash content in the biomass. They also reported a lower water content in the bio-oil from the washed biomasses, for example 24.7 and 17.2 wt % for unwashed and washed switch grass, respectively.^127^

Facas et al.^292^ reported that Ca can catalytically activate the intermonomer β-glycosidic bonds of cellulose, initiating reactions with volatile oxygenates. Millisecond reaction kinetics together with first-principles density functional theory calculations revealed a second-order rate dependence on Ca ion concentration in the Ca-catalyzed cellulose activation, and that it can proceed via a mechanism involving single- and cooperative-calcium-catalyzed glycosidic bond activation. In the single-Ca mechanism, Ca plays the primary role of stabilizing the charged transition states. In the cooperative mechanism, an additional Ca ion plays a secondary promotional role in disrupting the native H-bonding networks to enhance reactivity. Experimental activation energies were measured using cellulose surrogates α-cyclodextrin and doped (0.1–0.5 mmol Ca^2+^ *g*_*CD*_^‑1^) cyclodextrin. They reported that the Ca-catalyzed glycosidic bond activation was 48.7 ± 2.8 kcal/mol compared to 53.7 ± 1.1 kcal/mol for the noncatalyzed glycosidic bond activation energy at temperatures above 467 °C. At temperatures below 467 °C, the apparent activation energy is significantly lower (23.2 ± 1.9 kcal/mol) indicating a catalytic mechanism in the cellulose molten phase due to a hydroxyl-catalyzed mechanism. A similar study^305^ for the activation of cellulose via Mg ions indicated that Mg significantly catalyzes cellulose activation with a second-order rate dependence on the catalyst concentration, with an experimental activation energy of 45.6 ± 2.1 kcal/mol. Mg showed a dual catalyzed mechanism where both Mg ions interact with the oxygen atoms of hydroxyl groups to destabilize hydrogen bonds with hydroxymethyl groups. These studies suggest that Mg^2+^ and Ca^2+^ exhibit similar catalytic behavior in cellulose activation via transglycosylation at the intermonomer glycosidic bond.

#### 4.4 Catalytic Role of Inorganics in Hydrothermal Processing

Hydrothermal processing (HTP) is a thermochemical conversion process that uses water as the reaction media at elevated temperatures and pressures, with the advantage of handling wet biomass with a moisture content of up to 98 wt %.^306,307^ HTP operates under subcritical or supercritical water conditions at temperatures ranging from 200–800 °C and pressures from 50–400 bar, typically carried out in the absence of oxygen.^308–310^ HTP produces char, an aqueous organic phase, a nonaqueous phase (bio-oil), and gases. The gases are primarily H_2_, CO, and CH_4_, while the biocrude is an emulsion that is described in Section 4.2.5.

HTP occurs in different reaction zones depending on the desired product as shown in Figure 10. “Hydrothermal carbonization” (HTC) occurs at temperatures between 180 and 250 °C and pressures between 20 and 100 bar, where high char yields are obtained.^309,311,312^ At higher temperatures (200–374 °C) and pressures (50–220 bar) the process is known as HTL, in which higher biocrude yields are produced. Above the critical point of water (373 °C and 220 bar), the water properties change as it becomes a supercritical fluid, facilitating supercritical water gasification (SCWG) of biomass, with H_2_, CO, and CH_4_ as the main products.^312^ SCWG can also be divided according to the desired product; it can be low-temperature SCWG (374–550 °C) to produce a methane-rich gas product or high-temperature SCWG (550–700 °C) to produce a hydrogen-rich gas product.^313,314^

**Figure 10:**
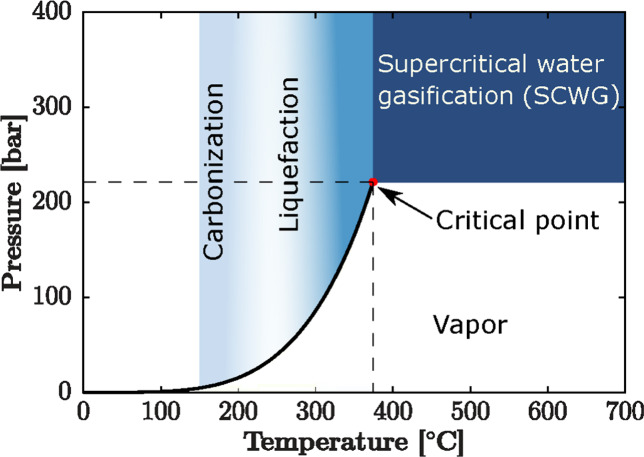
Various regimes in the HTP of biomass. Adapted with permission from Ayala-Cortés et al.^310^ Copyright 2021, Elsevier.

The physicochemical properties of water are a function of pressure and temperature. For instance, the density and viscosity of water decrease from 1,000 to 600 kg·m^–3^ and from 0.9 to 0.2 cP, respectively, from ambient conditions (25 °C and 1 bar) to near-critical water conditions (374 °C and 220 bar).^315^ As water approaches the critical point, its dissociation increases, which enhances the concentration of hydronium (H_3_O^+^) and hydroxide (OH^–^) ions.^316,317^ These ions create a highly reactive environment for acid- and base-catalyzed hydrolysis reactions^317,318^ with various types of biomass components. These characteristics make water in hydrothermal conditions a versatile substance, capable of acting as a solvent and a reactant.^319^

The impact of ash on HTP has been previously reported.^320–323^ Some inorganics catalyze HTP reactions and influence product distribution and fuel properties. In the HTC regime, water under subcritical conditions exhibits physicochemical characteristics that can enhance the removal of simple ionic salts from biomass. HTC can remove up to 57 wt % of the ash content from biomass by dissolving alkali salts in water.^324^

Minerals in biomass (e.g., algae) and reactor walls catalyze hydrothermal processes, enhancing H_2_ production through the water–gas shift reaction.^325^ Naturally occurring salts and metals also improve biomass conversion efficiency, thereby increasing biofuel yields. These findings underscore the critical role of inorganics in optimizing hydrothermal energy systems. Additional studies^323,326^ report the influence of ash in algae feedstock under HTP conditions and its effect on product distribution.

Often, inorganics are added to the HTL process to improve overall bio-oil yield. Studies commonly use low to moderate mineral loadings (≤10 wt %) to replicate naturally occurring levels in biomass or modest catalytic additions as summarized in Table 10. However, some research^323,327–329^ has explored higher concentrations (>10 wt %), primarily to study catalytic effects or to simulate ash-rich feedstocks. A comparison of the effects of representative minerals and elements is presented in Table 10.

**Table 10:** Influence of Minerals in the HTP of Biomass in Batch Reactors.

T (°C)	P (bar)	Τ (min)	HTP	Biomass	Main findings	Ref.
300	20	15	HTL	White birch barks	The addition of K in the form of K_2_CO_3_ to the deashed white birch bark demonstrated an increase in bio-oil yield and bark conversion from 58% and 86% to 67% and 92%, respectively. The addition of Ca(OH)_2_ to the deashed white spruce increased the conversion from 85% to 94% with a negligible effect on the bio-oil yield. Both K_2_CO_3_ and Ca(OH)_2_ enhanced the bio-oil quality in terms of O/C ratio and higher HHV.	^106^
280	–	15	HTL	Cherry and cypress	The addition of a 1 M K_2_CO_3_ solution improved the conversion of cherry and cypress wood from 69% and 56.3% to 99% and 94.9% respectively, while the total bio-oil increased from 15.7% to 43.2% for cherry wood and from 7.7% to 49.5% for cypress wood. The char yield decreased from 30.7% to 1.3% for cherry wood and from 43.7% to 5.1% for cypress wood, respectively. Additionally, the alkaline solution promoted the formation of acetic acid.	^330^
600	200	60	SCWG	Xylose	The addition of 10 wt % K_2_CO_3_ to the SCWG of xylose increased the gas yield from 69.4% to 86%, and the solid residue was reduced from 17% to 5.1%. The H_2_ and CO_2_ yields increased from 16% and 15.5% to 18.8% and 18.6%, respectively.	^331^
290	3.4	30	HTL	Bamboo	Adding 20 wt % K_2_CO_3_ to the HTL of waste bamboo chopsticks improved the bio-oil yield from 3.8 wt % to 21.2 wt % and slightly increased the HHV from 29.7 to 31.6 MJ·kg^–1^.	^332^
300	–	60	HTL	Lignin from corn stalks	The addition of alkali and alkaline earth metals individually in the form of chlorides (KCl, NaCl, CaCl_2_, and MgCl_2_) slightly improved the bio-oil yield compared to their mixtures. The addition of AAEMs increased the bio-oil yield for glucose and xylan from 13.9 and 11.9 wt % to 19.6 and 14.7 wt %, respectively. The addition of AAEMs to lignin resulted in a slight decrease in bio-oil yield, from 49 to 45 wt %.	^333^
320	–	15	HTL	Oak wood biomass	Different oxidation states of Fe (Fe^0^, Fe_3_O_4_, and Fe_2_O_3_) exhibit varying catalytic activities. Ten wt % of Fe^0^ catalyst with respect to the biomass increased the bio-oil yield from 26% to 40%. Fe_3_O_4_ showed an intermediate catalytic activity, increasing the bio-oil yield from 26% to 38%, while Fe_2_O_3_ showed no improvement in bio-oil yield.	^334^
300	20	30	HTL	Pinewood sawdust	Synergistic effects with different catalysts (Fe, Na_2_CO_3_, NaOH, FeSO_4_, MgO, Ru/C, and FeS), especially mixtures of 10% Fe and 10% of alkali catalysts (e.g., Na_2_CO_3_, NaOH) or FeS, were observed on the HTL of pinewood sawdust. The highest (48%) bio-oil yield was obtained with Fe + Na_2_CO_3_, compared to a 25% bio-oil yield without a catalyst. Bio-oil yields of around 45.7% and 46.2% were obtained using Fe + NaOH and Fe + FeS, respectively. Ru/C produced the bio-oil with the highest HHV (30.9 MJ·kg^–1^).	^335^
350	–	30	HTL	Kenaf	Different base catalysts were investigated: Ca(OH)_2_, Na_2_CO_3_, K_2_CO_3_, and KOH. The addition of 1 wt % Na- and K-based catalyst decreased the repolymerization reactions and the yields of both light and heavy oil fractions. The addition of any of these catalysts enhanced the HHV of the light oil from 28.38 in the noncatalytic process to 32.3–34.2 MJ·kg^–1^.	^336^
250	40	120	HTC	Rice straw	5 wt % K compounds (e.g., K_2_SO_4_, KCl, KOH, and K_2_CO_3_) were used in the HTC of rice straw. K_2_SO_4_ increased the HHV of the resulting hydrochar from 23.3 to 24.5 MJ·kg^–1^. No significant difference (<1%) in hydrochar yield was observed across the catalysts used.	^337^

##### 4.4.1 Alkali and Alkaline Earth Metals in HTL

Potassium in the form of K_2_CO_3_ is a highly effective catalyst in HTL, enhancing bio-oil yields (up to 68 wt %) and promoting the formation of acetic acid, 2-methoxyphenol, and formic acid.^338,339^ In SCWG, K_2_CO_3_ increases gas yields from 69.4% to 86% while maintaining gas composition (CH_4_, H_2_, CO_2_, CO, C_4_H_10_).^331^ Additionally, K-based additives improve activated carbon production by promoting the formation of porous carbon structures.^340,341^

Water-soluble chlorides (KCl, NaCl, CaCl_2_, MgCl_2_) exhibit distinct catalytic effects. Individual use of these salts in lignin HTL raises bio-oil yields from 48 wt % (noncatalytic) to 51–53 wt %, with K showing the highest efficacy.^342^ However, mixtures of alkali (K, Na) and alkaline earth metals (Ca, Mg) reduce yields to 44–46 wt %, indicating antagonistic interactions.^342^

Comparative studies rank alkali catalyst activities as K_2_CO_3_ > KOH > Na_2_CO_3_ > NaOH in sawdust HTL.^341^ Feng et al.^106^ demonstrated that K_2_CO_3_ and Ca­(OH)_2_ reduce solid residues by 30–80% in bark HTL, though bio-oil yields remain stable. Demineralization (acid washing) of biomass lowers ash content but reduces bio-oil yields (e.g., from 66% to 58% for birch), highlighting the catalytic role of native inorganics.

##### 4.4.2 Transition Metals (Fe) and Synergistic Effects

Iron-based catalysts (Fe_2_O_3_, Fe_3_O_4_, Fe^0^) enhance deoxygenation and light compound formation in bio-oil. Metallic Fe outperforms oxides, reducing char yields by >80% in oak wood HTL.^343^ At 300 °C, Fe_2_O_3_ increases palm empty fruit bunch bio-oil carbon content from 11% to 39%.^329^ Synergistic effects are observed with Fe–Na_2_CO_3_ mixtures, boosting pinewood bio-oil yields to 45 wt %.^344^

##### 4.4.3 Challenges in Catalyst Application

Despite their catalytic benefits, inorganic additives face three critical limitations in hydrothermal processing: (i) Recovery difficulties, as catalysts disperse across multiple product phases.^344^ Alkali metals (K, Na) dissolve in aqueous streams while Fe-based nanoparticles embed in biochar, requiring energy-intensive separation methods like magnetic recovery or acid washing. (ii) Progressive deactivation through leaching, sintering (surface area reduction in Fe catalysts after some cycles), or chemical poisoning (e.g., FeS formation from biomass S). (iii) Feedstock dependency, where variable ash composition (e.g., Al, Ca, Fe, K, Cl content) and biomass type (lignin vs cellulose) lead to inconsistent catalytic effects.^106^ For example, while CaCO_3_ in high-ash algae promotes ketonization, it simultaneously inhibits nitrogenous compound formation. In contrast, K_2_CO_3_ demonstrates minimal impact on cellulose-rich feedstocks despite its effectiveness with other biomass components.

Mineral mitigation strategies are under development. Magnetic Fe_3_O_4_ can be recovered from solid residues after processing using magnetic separation, while water-soluble K_2_CO_3_ and CaCO_3_ are extractable via aqueous washing. However, economic challenges persist due to feedstock-specific needs. For example, low-ash biomass may require catalyst addition, while high-ash feedstocks need pretreatment to remove deactivating elements. This underscores the necessity for developing catalysts that balance activity, stability, and recyclability for industrial use.

##### 4.4.4 Demineralization Trade-Offs

The acid washing of biomass creates a fundamental trade-off between yield and fuel quality. Whereas removing native inorganics reduces bio-oil yields by 11–19% (e.g., spruce HTL decreasing from 58% to 47%), it simultaneously improves fuel properties through oxygen removal, increasing HHV by 3–5 MJ·kg^–1^ (26–28 to 31 MJ·kg^–1^).^106^ This contrast reveals how biomass minerals serve dual roles, acting as natural conversion catalysts while simultaneously introducing fuel contaminants. Several strategic pathways exist: (i) selective removal of only detrimental inorganics while preserving beneficial catalytic components, if any, or (ii) post-treatment upgrading of inorganics-rich bio-oils. The optimal approach must be tailored to the specific inorganic composition of each feedstock. Notably, developing effective inorganic-removal methods could standardize biomass into a more consistent feedstock, approaching the uniformity of model compounds. Such standardization would enhance both process efficiency and biofuel quality by yielding products with more predictable physicochemical properties.

#### 4.5 Bio-Oils: Properties, Upgrading Strategies, and Challenges Related to Inorganic Elements

The bio-oil produced from HTP and pyrolysis contains a large variety of organic compounds, including carboxylic acids, carbohydrates, furans, ketones, aldehydes, saccharides, phenolic compounds, esters, ethers, and alcohols, forming a complex mixture with over 400 different oxygenated hydrocarbons.^345,346^ The high concentration of acidic compounds in bio-oils can lead to corrosion of storage containers. Other organic groups, such as aldehydes and phenols, are unstable, unsaturated, and prone to polymerize into larger compounds in the presence of acids, resulting in increased viscosity and poor fluidity, complicating transportation.^345^ The heterogeneity of bio-oils produced from pyrolysis and hydrothermal processing hinders their direct use as transportation fuels.^345,346^

The challenges with using bio-oils as fuels are ascribed to their characteristics, which include high oxygen content, high water content (15–50 wt %), high acidity, thermal and chemical instability, a tendency for high coke formation due to repolymerization, low HHV (16–19 MJ·kg^–1^), high volatility, and high inorganics content.^347^ Reducing the oxygen content of bio-oils improves their stability, volatility, and reduces molecular weight and viscosity.^346^

Upgrading treatments are necessary to improve the compatibility of bio-oils with existing petroleum engines.^348^ The primary goal is to reduce oxygen content and increase hydrocarbon content, making bio-oils more suitable as transportation fuels. Conventional upgrading treatments include hydrodeoxygenation (HDO), esterification, steam reforming, and fluid catalytic cracking^345,346^ which can all be influenced by the inorganic compounds present in the bio-oil. More discussion about HDO is provided in Section 6.1.3.

Most bio-oil upgrading technologies require the use of a catalyst,^349^ and contaminants from biomass, especially inorganics such as K, Mg, Ca, Na, S, P, Cl, and N, can potentially affect the catalyst performance and cause severe poisoning. The extent of their impact depends on the upgrading method, the catalyst type, and the reactor type and configuration.^350^ In *in situ* upgrading, in which the biomass and the catalyst are cofed during pyrolysis, the transfer of inorganics from the biomass to the catalyst and the ensuing deactivation are higher compared to the *ex situ* upgrading process, in which the upgrading process follows the pyrolysis process.^350^ Leijenhorst et al.^109^ reported that the inorganics transfer from biomass feedstocks to the bio-oil during pyrolysis depends on how they are attached to the biomass structure. Their results, shown in Figure 11, indicate that most of the inorganics (Ca, Mg, Fe, Cu, Ni, Cd, Cr, Co, Mn, Zn, Al, and Pb) remain on the char, implying that the incomplete separation of submicron char particles from the gaseous product prior to condensation is the main route for inorganics transfer to the pyrolysis bio-oil.^351^ Na and K are transferred additionally via secondary reactions with volatile compounds. S and P are transferred primarily due to reactions with organic volatiles. In general, around 95% of the inorganics in biomass feedstocks remain in the char, with 5% to 10% of inorganics typically transferred to the bio-oil.

**Figure 11:**
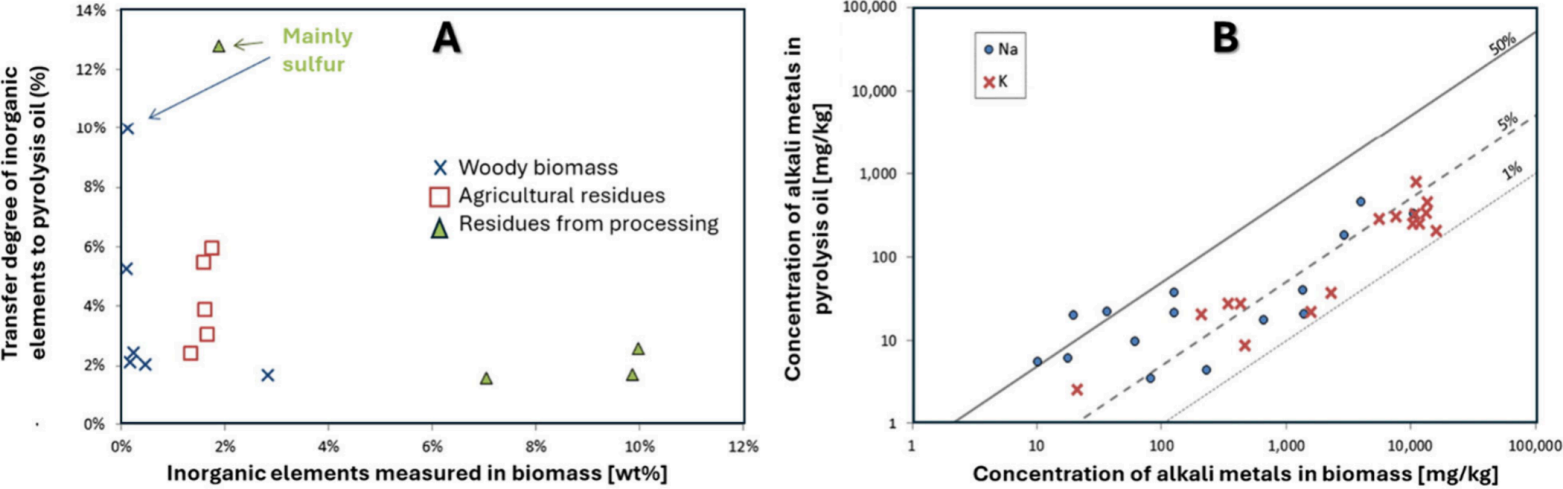
A) Transfer degree of inorganics from biomass to the pyrolysis bio-oil, and B) concentration of Na and K in pyrolysis oil as a function of initial feedstock concentrations. The % lines represent the ratio of the concentrations of the inorganics in the pyrolysis oil normalized with their concentrations in the biomass. Reproduced with permission from Leijenhorst et al.^109^ Copyright 2016, Elsevier.

Keown et al.^144^ investigated the effects of volatile-char interactions on the volatilization of AAEMs species during pyrolysis of two sugar cane industry wastes, bagasse and cane trash. They found that such interactions could lead to additional volatilization of AAEMs species, as manifested in additional char weight losses. They indicated that reactive species (e.g., CO_2_ and H_2_O) in the volatiles could lead to the gasification of char during the volatile-char interactions, with the exact extent of char weight loss depending on the composition of the volatiles and the structure of the char.

Leijenhorst et al.^109^ summarized some important aspects regarding the mechanisms of AAEMs release from biomass, including:

1. The chemical/physical form of the element in the solid material is important for its release to the gas phase.^109^ Pyrolysis experiments of pulverized biomass in a wire-mesh reactor and the analysis of the retained species in the char by ion chromatography demonstrated that Na, K, Ca, and Mg in free ion form are released to a larger extent than the hydroxide and carbonate forms, with chloride species being the least volatile among them.^109,352^

2. Below 600 °C, the transfer of inorganics from the biomass to the gas phase occurs when organic volatiles bind to the inorganic species in the char, rendering them more volatile.
3. The migration of inorganics to the gas phase can be affected by both intraparticle mass transfer limitations within biomass particles and interphase mass transfer limitations between the particle surface and the bulk gas phase, dictated by the gas phase space velocity past the biomass particle and the reactor pressure that affects pore diffusion rates.
4. Higher heating rates favor the transfer of inorganics to the gas phase because of a higher concentration of volatiles that can react with the inorganics.
5. The gas used in the thermochemical process can also affect the release of inorganics from biomass. For example, CO_2_ can interact with and intercept the organic volatiles, thereby competing with the inorganic elements and limiting their release to the gas phase. For example, Na is retained better in the char particles in a CO_2_ atmosphere compared to an inert argon atmosphere that does not interact with the organics.

Biomass pretreatment, such as water and acid washing, can reduce the inorganics, thereby reducing their transfer to the bio-oil during biomass pyrolysis. Other approaches to remove inorganics after biomass pyrolysis, such as bio-oil cold filtration, are reported to be less effective for inorganics removal from bio-oil to an acceptable level. In sharp contrast, hot gas filtration of pyrolytic vapors significantly reduced the alkali metals to levels below 10 ppm.^353^ The higher inorganics reduction on the filtered bio-oil by using hot gas filtration could be related to the removal of the submicron ash particles just after the pyrolysis reaction (with no solvent interference). In contrast, cold filtration was done once the bio-oil was collected using acetone as a solvent, meaning that some of the inorganics in the submicron ash particles could have been leached to the acetone solution. Although the authors reported no leaching of inorganics from the ash particles in the bio-oil over 30 days of storage, leaching could have occurred during the initial contact between the acetone and the ash particles. For example, fresh bio-oil from switchgrass pyrolysis contained 319, 95, and 52 ppm of K, Ca, and P, respectively. After filtration through a series of filters (60 to 0.7 μm pore size), the inorganic content was reduced to 175, 2.2, and 0.6 ppm for K, Ca, and P, respectively, after passing through the finest filter (0.7 μm pore size). When the filtration was done at a high temperature (390 °C) using a 2 μm pore size filter (316 SS filter), the concentrations of the same elements (K, Ca, and P) were below 10 ppm. Lindfors et al.^354^ reported the hot-filtration of bio-oil from contaminated wood and forestry residues, with only slight changes observed in the content of Na, Mg, K, Ca, and other inorganic elements after hot vapor filtration. The nonfiltrered bio-oil from contaminated wood contained 84, 28, 18, 51, 180, and 260 ppm of Na, Ca, Fe, Si, S, and Cl, respectively. In contrast, the resulting bio-oil after hot filtration had 70, 10, 2.4, 14, 110, and 190 ppm of Na, Ca, Fe, Si, S, and Cl, respectively. Other elements, such as K, Mg, Cr, Mn, Cu, Zn, Pb, and P, showed very small or no removal during the filtration.

Jendoubi et al.^355^ compared the inorganics distribution in the char and bio-oil fractions of straw and beech wood pyrolyzed in a fluidized bed reactor. They reported that almost 99% of the initial inorganic content of biomass feedstocks remained in the chars recovered from cyclones, and only around 1% was recovered in bio-oils. The product vapor from the pyrolyzer was collected as various fractions. Heavy oils were recovered at 90 °C, light oils were condensed at room temperature, and the aerosols containing submicron char particles were recovered using an electrostatic precipitator. The remaining vapor was condensed in a liquid nitrogen/dry ice trap (−30 °C). They found that 60–79% of K, Ca, and Mg, and 50% of Na partition into the aerosols. These results confirmed that most inorganics transferred to the bio-oil from biomass pyrolysis come from submicron char particles.

As explained in the foregoing paragraphs, inorganics in pyrolytic bio-oil can pose challenges during its catalytic upgrading and also affect its long-term storage stability. Hwang et al.^356^ studied the storage stability of four types of bio-oil with varying inorganic content controlled by demineralization and impregnation of yellow poplar, for periods of 1, 2, 4, and 8 weeks at 23 °C. They reported increased bio-oil viscosity, average molecular weight, and solid content at longer storage durations. Additionally, they reported a progressive decrease in the concentrations of aldehydes, ketones, and levoglucosan. They found that the inorganic elements were initially concentrated in particles present in bio-oil and tend to leach out from the particles into the liquid phase during storage. The authors suggested that inorganics (e.g., K, Mg, and Ca) can influence the aging characteristics of bio-oil during storage by enhancing catalyzed polymerization reactions. For example, higher concentrations of K and Mg caused more changes in bio-oil properties, such as increased viscosity due to a higher concentration of high-molecular weight compounds compared to lower concentrations of these inorganics.

As outlined above, up to 10% of the inorganic elements in biomass can be transferred to the bio-oil during pyrolysis and an even higher amount in hydrothermal carbonization^357^ (up to 90% transfer of P, Ca, Mg, K, Na, and Mg from the biomass to the liquid phase at 180 °C). Hence, advanced technologies to remove inorganics from either the bio-oil or the biomass feedstocks are needed to mitigate their detrimental effects during bio-oil storage and its catalytic upgrading processes. Some of these technologies involve inorganic removal from biomass using leaching agents (such as water, acid solutions, or chelating agents), while others remove inorganics from the hot-vapor streams using filters, or removing aerosols using electrostatic precipitation, or subjecting the bio-oil to an adsorption process. Månsson et al.^358^ reported the use of γ-Al_2_O_3_ for P adsorption, achieving 85% removal, and the use of zeolites and resins to remove between 85 and 98% of certain inorganic elements (Ca, K, Mg, and Fe) at 30 °C. By selectively removing inorganic impurities in this manner, these methods can prevent catalyst deactivation during bio-oil HDO.

#### Section Summary

In this section, we have summarized the role of inorganics in thermochemical approaches for converting biomass, such as combustion, gasification, pyrolysis, and hydrothermal liquefaction. Inorganics in biomass cause wear, ash or char agglomeration, deposits, slagging, fouling, and corrosion of equipment in thermochemical processes, especially in combustion, pyrolysis, and gasification. During gasification, tars can lead to similar issues as the ones encountered with inorganics. Therefore, effective methods to prevent or mitigate tar formation are necessary. Since inorganics can have a catalytic effect on tar formation and elimination, careful consideration and process optimization are necessary to design mitigation strategies for biomass feedstocks. For example, K and Ca can improve the char reactivity during catalytic gasification under a CO_2_ atmosphere. In contrast, the gasification rate of acid-washed char is very low.^235^ N from biomass can be released in different species; HCN and NH_3_ are especially relevant as they are primary precursors of NO_*x*_ that can further react in the presence of water to form HNO_3_. Studies show that CaO can reduce the yields of HCN, NH_3_, and HNCO, while iron minerals tend to suppress HCN.^245^ During fast pyrolysis and hydrothermal processing, the primary catalytic effects of inorganics are to lower the decomposition temperatures and change the product selectivities and yields. Approximately 10% of the inorganics in biomass are transferred to the bio-oil product during pyrolysis. In sharp contrast, nearly 90% of the inorganics in the biomass feed are transferred to the liquid phase product during HTL and HTC. Inorganics transferred to the bio-oils pose challenges during bio-oil upgrading, as they can cause permanent deactivation of the catalysts and/or alter the selectivities of the desired products. Additionally, inorganics negatively impact long-term bio-oil stability, necessitating their removal from the bio-oil prior to downstream processing either through filtration or adsorption methods or by removing inorganics from the biomass feedstocks in a pretreatment step.

## 5 Influence of Inorganics on Biochemical Pathways for Biomass Conversion

### Section Overview

In this section, we discuss the potential challenges that inorganics pose in the biochemical conversion of biomass. The biochemical pathway typically involves pretreatment, enzymatic hydrolysis, and the fermentation of sugars. Herein, we focus on the effects that inorganics have on acid neutralization during the acidic pretreatment step and their interactions with the biomass structural components through complexation, which reduces pretreatment efficiency. We discuss how high inorganic content in biomass negatively impacts enzymatic hydrolysis by altering the enzyme structure and reducing catalytic activity. We examine how inorganics in limited quantities are essential for microbial growth during fermentation but in excess can disrupt the internal cellular environment in fermentative microorganisms, causing osmotic stress and cell membrane damage that reduces product yields. Additionally, we discuss the role of inorganics in the anaerobic digestion of biomass for biogas production, where inorganics are essential for optimal biogas production, but an excess can be detrimental to the process.

Biochemical methods utilize microorganisms and enzymes to convert renewable materials like biomass into chemicals and fuels.^359^ These methods offer sustainable alternatives to fossil fuel-dependent processes. For instance, microbial fermentation uses bacteria or yeast to transform sugars derived from biomass into biofuels, such as ethanol, and chemicals like lactic acid. Anaerobic digestion breaks down organic materials without oxygen, generating biogas that is composed mainly of methane and CO_2_. Biocatalysis employs enzymes to speed up specific chemical reactions, while metabolic engineering involves modifying microorganisms to enhance or create pathways for producing advanced biofuels, such as isobutanol. Biochemical processes typically begin with preparing biomass feedstock through pretreatment and enzymatic hydrolysis. This is followed by fermentation, which takes place in controlled environments to yield the desired products. Finally, downstream processes, such as distillation or extraction, are employed to recover and purify the final product.^359^ Integrated biorefineries streamline these steps to efficiently produce fuels, chemicals, and energy from renewable resources.

The complex structure of lignocellulosic biomass and the presence of inorganics pose challenges for effective biochemical conversion. Minerals taken up by the plant during growth primarily originate from the environment, and are derived from soil, fertilizer, irrigation water, and environmental pollution. Soil contamination during biomass growth, harvesting, handling, and storage can also introduce minerals onto the external surface of the biomass. The presence of minerals and their varied concentrations influence the efficiency and cost-effectiveness of biomass conversion processes.^360^

Biomass inorganics impact each stage of the conversion process. During acidic pretreatments, they can neutralize pretreatment chemicals and interact with structural carbohydrates, such as cellulose and hemicellulose, forming complexes that reduce their accessibility during enzymatic hydrolysis.^361^ Divalent ions, such as Ca^2+^ and Mg^2+^, can bind to lignin or polysaccharides, complicating the breakdown of biomass and creating unfavorable reaction conditions, such as pH imbalances that hinder pretreatment efficiency.^362^ Recent studies suggest that high inorganic concentrations can lead to decreased yields of fermentable carbohydrates, disrupting fermentation and enzymatic hydrolysis, and ultimately reducing biofuel production efficiency.^362^ High ash content in biomass, particularly above 5%, poses challenges for biological processing.^363^ It reduces the availability of fermentable sugars, inhibits enzymatic activity, and affects microbial metabolism. The specific impact depends on the type and concentration of inorganics, the pretreatment method used, and the nature of the biomass. Understanding the influence of these inorganics is essential for optimizing biomass conversion strategies, improving yields, and developing effective mitigation techniques.

The role of inorganics in biochemical conversion processes is often overlooked, as the conventional focus has been on the yields of primary biomass components like cellulose, hemicellulose, and lignin.^10^ This oversight has created critical knowledge gaps that hinder efforts to address inorganic-related inefficiencies. Soil is a considerable source of biomass mineral contamination (as discussed in Section 3.1.2), as it can inhibit biochemical processes such as pretreatment, enzymatic hydrolysis, and fermentation. This section will focus on these issues and discuss potential solutions to mitigate these challenges.

#### 5.1 Soil Contamination: A Significant Contributor to Mineral Content of Plants

Soil contamination impacts the mineral content in plants and biomass feedstock. As explained in Sections 2.8.1 and Section 3.1.2, minerals can either be absorbed during plant growth or adhere to plant surfaces during harvesting and handling processes of agricultural residues or dedicated energy crops. This interaction can lead to increased inorganic content in the biomass.^364^ While these minerals are vital for plant development, excessive mineral presence—caused by factors like overfertilization, contamination from heavy metals, or degraded soil—can lead to higher inorganic content in the biomass. Improving handling and cleaning techniques can significantly reduce the mineral retention, lowering the inorganic content.^365^ This is particularly important for crops harvested near the ground, where contact with soil is more likely.

Soil contamination, often stemming from farming practices, environmental conditions, and soil composition, presents both challenges and opportunities for improving the biochemical conversion of biomass into fuels and chemicals.

Minerals that enter biomass via soil contamination affect its processing properties. For example, silicon dioxide (SiO_2_) poses challenges due to its abrasive nature, which can cause wear on grinding mill equipment. Ca and Mg play crucial roles as acid neutralizers during pretreatment processes (Figure 12A), particularly in acidic pretreatments, as discussed later in detail.^370^ Reducing the presence of Ca and Mg can enhance the effectiveness of acidic pretreatments, potentially reduce chemical costs, and improve sugar yields during the conversion process. In addition to neutralizing acids, alkali metals, including K and Na, can also influence pretreatment processes by forming salts that limit enzymatic access to cellulose and hemicellulose. Addressing this interference can enhance processing efficiency (Figure 12B) and mitigate contamination issues in intermediate products, ultimately lowering water treatment costs. While Fe and Al can sometimes disrupt enzymatic hydrolysis, they can also act as catalysts in controlled amounts.^14^ Leveraging this knowledge allows us to optimize sugar yields and leads to more effective biomass conversion processes. By focusing on these aspects, we can turn the challenges of soil contamination into opportunities for innovation and efficiency in biomass processing.

**Figure 12:**
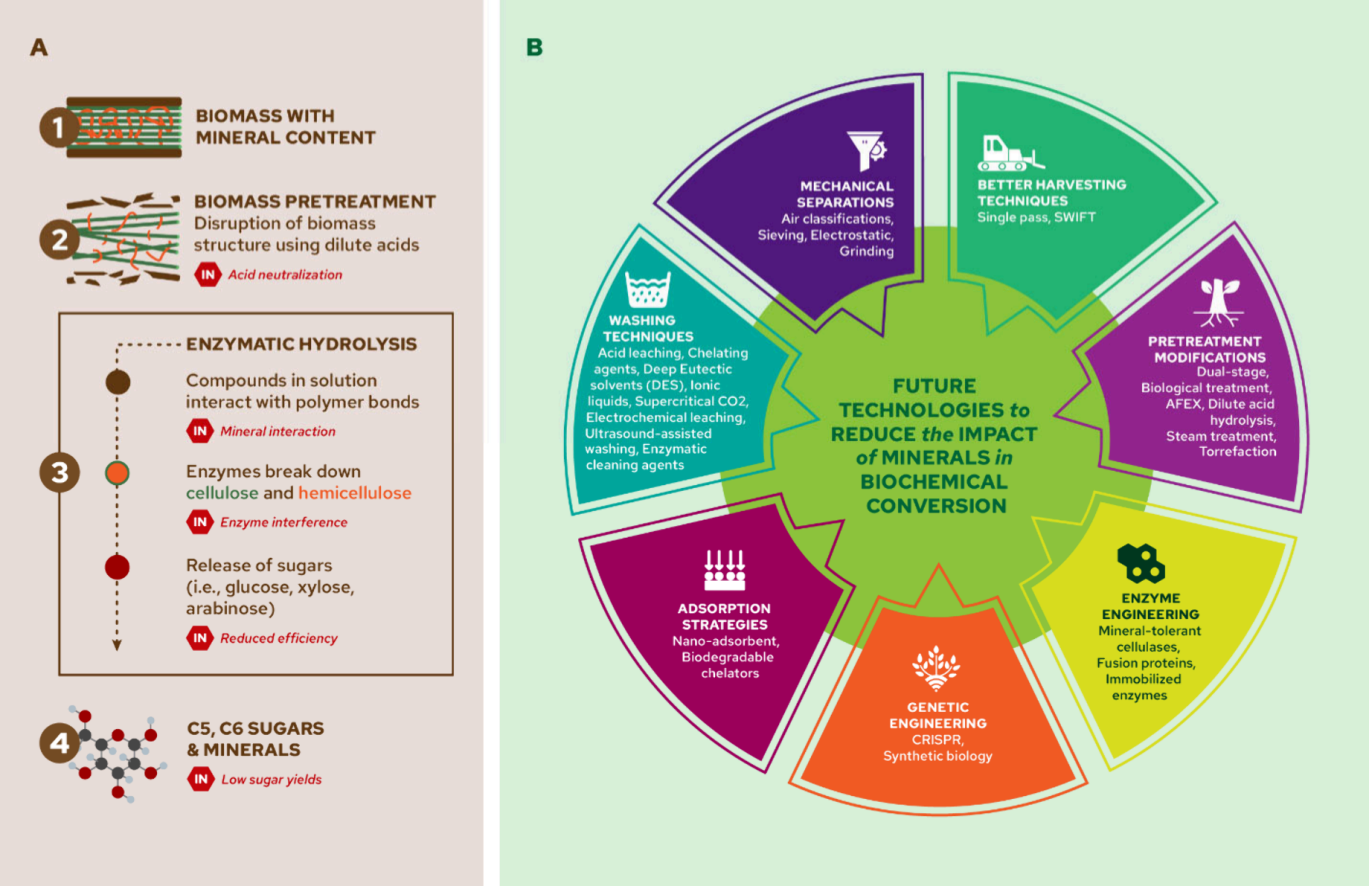
A) Pretreatment and lignocellulosic biomass conversion through enzymatic hydrolysis, showing the impact of inorganics on the total sugar yield. B) Future technology needs to reduce the impact of mineral content during the biomass conversion process. CRISPR: clustered regularly interspaced short palindromic repeats.^366^ AFEX: ammonia fiber expansion process.^367,368^ SWIFT: single pass, weather independent fractionation technology.^369^

By raising the cutting height during the harvest of crops such as corn stover, wheat straw, and sugar cane bagasse, farmers can reduce the risk of soil contamination and achieve a cleaner harvest.^371^ Timing is also crucial; harvesting in dry weather can prevent soil particles from adhering to damp plants, thereby maintaining cleanliness and quality. After harvest, mechanical cleaning methods like shaking, sieving, and blowing air can effectively reduce residual soil. Additionally, washing the biomass with clean water or dilute solutions helps eliminate external contamination; however, it is essential to manage the water effectively. Farmers can employ precision fertilization and site-specific nutrient management techniques to further mitigate soil contamination.^371^ Regular soil tests provide valuable insights into the mineral concentrations in the soil, allowing for informed adjustments to agricultural practices that optimize conditions for plant health and yield. Techniques such as acid washing (described in Sections 7.3.3–7.3.5), can dissolve water-soluble inorganics, including K and Na. This process reduces their concentration in subsequent processing stages, resulting in higher-quality biomass. Installing specialized ash separation equipment during preprocessing removes unwanted solid particulates and minerals. This step ensures that only clean biomass material is utilized in biochemical conversion processes, ultimately enhancing efficiency and output quality.

As shown in Figure 12B, a combination of on-field practices, advanced cleaning and preprocessing technologies, and scientific research is necessary to mitigate the issue of mineral contamination in biomass. These strategies, coupled with innovations in feedstock development and process optimization, can significantly improve the efficiency and sustainability of lignocellulosic biomass conversion. Moving forward, integrating agricultural practices with advanced biochemical engineering will play a key role in addressing the mineral contamination issue and unlocking the full potential of biomass for biofuel and chemical production.

#### 5.2 High Plant Mineral Content Has Dual Effects on Pretreatment and Enzymatic Hydrolysis of Biomass

Inorganics content can affect pretreatment effectiveness. In methods like dilute acid pretreatment, acids such as sulfuric acid help break down hemicellulose. However, minerals can neutralize the acid, requiring more acid to reach the desired pH, which increases chemical costs and lowers efficiency. For example, a corn stover with a high ash (minerals) content needs more acid to produce the same sugar yield as a low-ash corn stover. High mineral content can result in additional challenges, including a decrease in essential components such as cellulose.^372^

Inorganics such as Ca^2+^ and Mg^2+^ can form insoluble salts with the acid. For example, autohydrolysis, a pretreatment strategy that uses hot water to treat biomass, relies on the acetic acid released from the acetyl group on biomass hemicellulose. However, autohydrolysis of wheat straw with high ash content (>13%) is less effective, with reduced xylan removal and enzymatic hydrolysis efficiency. High inorganic content buffers the solution, reducing the concentration of protons (H^+^) needed to catalyze the breakdown and dissolution of hemicellulose.^373^ This buffering effect leads to poor pretreatment, resulting in reduced hemicellulose removal and minimal structural changes in cellulose. Consequently, there is a lower surface area for enzymes to act upon, decreasing sugar release during enzymatic hydrolysis. Interestingly, while higher inorganics content generally results in inefficiencies, its effects can vary depending on the biomass and pretreatment method used. For example, studies on mixtures of hybrid poplar (1.3–1.5 wt % ash content) and wheat straw (5–8 wt % ash content) have shown that inorganics in wheat straw can reduce acidity during pretreatment while simultaneously improving (40–55%) sugar recovery by mitigating sugar degradation.^374^ This behavior highlights the complex interplay between inorganic content, biomass composition, and pretreatment outcomes.^35^

Following pretreatment, enzymatic hydrolysis is used in the biochemical conversion of lignocellulosic biomass. Cellulolytic and xylolytic enzymes break cellulose and hemicellulose into fermentable sugars, which are then converted into ethanol, biochemicals, or other biobased products. However, the inorganic content can adversely affect enzymatic hydrolysis efficiency.

High inorganic content negatively impacts enzymatic hydrolysis by altering enzyme structure and reducing catalytic activity. K^+^ and Mg^2+^ can disrupt enzyme function and impede sugar release from hydrolyzing cellulose and hemicellulose.^35,375^ Previous studies^376,377^ have reported that wheat straw with an ash content exceeding 13% resulted in a 19% reduction in xylan removal during pretreatment and an 18% decrease in enzymatic hydrolysis efficiency.

Fitria et al.^35^ found that the adverse effects of minerals during pretreatment are due to their self-buffering properties, which decrease substrate accessibility. Their experiments showed that increasing the ash content in corn stover from 8.3% to 13.2% reduced sugar yields by 5.9% to 14.6% after pretreatment and by up to 6.7% during enzymatic hydrolysis. In contrast, alkaline pretreatment resulted in a roughly 3.1% decrease in sugar yields after pretreatment and a 0.9% reduction during hydrolysis. Sha et al.^362^ found that water washing to reduce mineral content of untreated corn stover led to a decrease in pH for both untreated and DLCA (Densifying Lignocellulosic biomass with Chemicals followed by Autoclave) pretreated corn stover (1.5 to 1.0 for DLCA + sulfuric acid; 10.5 to 9.0 for DLCA + calcium hydroxide), suggesting removal of alkaline elements (K, Na, Mg, and/or Ca). The removal corresponded to a significant increase in sugar conversion during enzymatic hydrolysis for the acidic pretreatment (DLCA + sulfuric acid) and a slight decrease in conversion for the alkaline pretreatment (DLCA + calcium hydroxide). These effects are related to the change in pH and catalytic activity of the pretreatment. A similar pattern was observed by Huang et al.^375^ and He et al.,^378^ who also used water washing to reduce the ash content from high ash fraction of wheat straw and corn stover, respectively. The loss of ash corresponded to a decrease in prehydrolysate pH (5.7 to 4.3 for liquid hot water pretreatment and 4.2 to 2.8 for dilute sulfuric acid pretreatment) and a significant increase in sugar recovery from enzymatic hydrolysis for both processes. He et al.^378^ evaluated the elemental composition of their demineralized corn stover and found an increase in Si, and a loss of Mg and K with decreasing ash content, with no obvious trend for Ca, Al, S, and P. This suggests that the removal of alkaline minerals containing Mg and K increases the efficiency of acidic pretreatments. The acceptable level of ash content in a feedstock varies depending on the pretreatment method used.

Inorganics like Ca^2+^, Mg^2+^, Fe^3+^, and Al^3+^ can bind to enzymes or active sites, altering their structure and reducing their catalytic efficiency. For example, the adsorption of Ca^2+^ onto cellulases can hinder their ability to attach to cellulose, leading to reduced sugar yields. Soluble ions such as Na^+^ and K^+^ can compete with enzymes to bind to biomass substrates, lowering enzyme efficiency. These ions disrupt the enzyme–substrate interaction by altering the charge and chemical environment of the reaction.^379^ Some minerals, such as SiO_2_, can form insoluble precipitates with biomass or enzymes. These precipitates reduce the bioavailability of both enzymes and substrates, decreasing the hydrolysis rate.^380^

Despite various mitigation strategies, including water washing and acid leaching, the cost-effective removal of ash remains a bottleneck, particularly for low-cost, high-ash feedstocks like agricultural residues. A key challenge is the diverse composition of biomass and its variable inorganic content, which influences the efficiency of different pretreatment methods. While alkaline and oxidative pretreatments exhibit better resilience to inorganic interference, they often come with higher operational costs and the risk of side reactions that degrade fermentable sugars. Furthermore, the lack of standardized inorganics removal protocols limits the ability to optimally apply demineralization strategies across different feedstocks and industrial settings. Future technologies for mitigating the impact of inorganic content in biomass conversion need to focus on innovative washing, pretreatment, enzyme engineering, adsorption, and genetic modification strategies, as shown in Figure 12B. When integrated, these emerging technologies may offer promising solutions for enhancing biomass processing efficiency and improving biofuel yields and endow future biorefineries the versatility to process a broad variety of feedstocks from different biomass sources.

#### 5.3 Mineral-Microbe Interactions in Fermentation and Anaerobic Digestion

Minerals are crucial for microbial activity, but excess minerals can inhibit processes, cause toxicity, or alter pathways, leading to reduced end-product yields. The effects vary based on the type and concentration of minerals and the microbial community involved. Mineral-microbe interactions can have either a positive or negative impact on digestion, depending on the type, concentration, and bioavailability of the minerals.

##### 5.3.1 Impact of Ash Content on Fermentation Efficiency and Biofuel Production

The presence of inorganics such as Na^+^, K^+^, and Cl^–^ can hinder the fermentation efficiency of biomass-derived sugars into biofuels and other products. These inorganics can disrupt the internal cellular environment, causing osmotic stress and potential damage to cell membranes (Figure 13A).^381^ Given that fermentative microorganisms are already challenged by highly inhibitory conditions during biofuel production, an excess of these inorganics can further reduce fermentation yield.^362^

**Figure 13:**
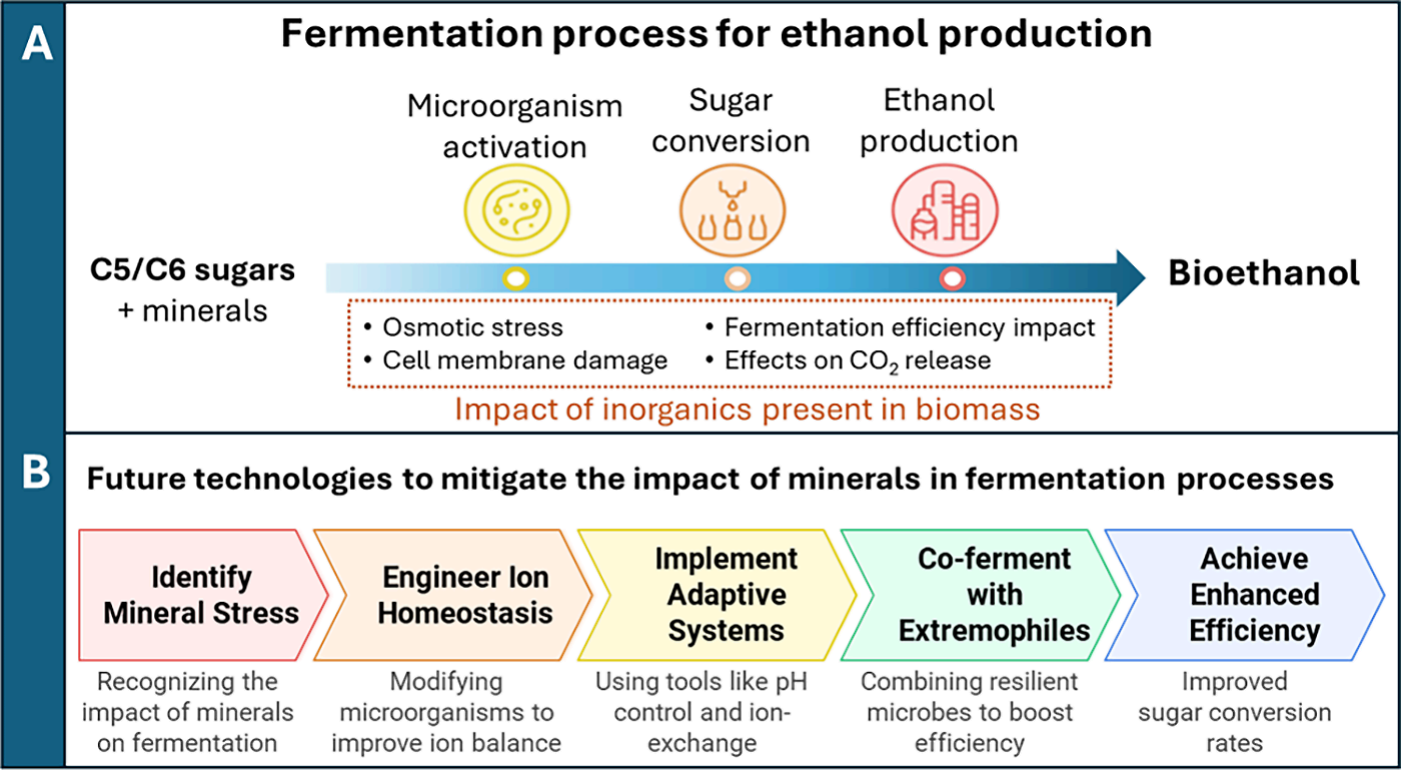
A) Minerals can reduce the efficiency of fermenting simple sugars into bioethanol by disrupting the internal cellular environment, causing osmotic stress and potential damage to cell membranes. B) Technologies to mitigate the impact of minerals on the fermentation process include microorganism engineering, control of the microbial environment (e.g., pH and ion balance), and cofermentation strategies.

Sha et al.^362^ investigated the effect of ash content on ethanol fermentation using DLCA pretreated corn-stover (DLCA-CS) hydrolysates and *Saccharomyces cerevisiae* CRD5HS. The hydrolysates were produced from sulfuric acid-pretreated (DLCA (sa)-CS) and calcium hydroxide-pretreated (DLCA (ch)-CS) corn stover, respectively. DLCA (sa)-CS hydrolysate achieved near-complete sugar consumption within 72 h, reflecting high fermentation efficiency. On the other hand, DLCA (ch)-CS hydrolysate exhibited slightly slower sugar utilization, mainly when ash content was higher than 5.5 wt %, leaving some residual xylose unconsumed. The authors attributed this to higher initial sugar concentrations in DLCA (ch)-CS hydrolysates. Optimal ethanol production was observed at an ash content of 3.4 wt %, yielding 60.7 g/L ethanol in DLCA (sa)-CS and 66.5 g/L ethanol in DLCA (ch)-CS). Increased ash content (9.5 wt %) reduced ethanol yields by around 2 g of ethanol per g of biomass for both acid and alkaline pretreatment, likely due to lower sugar release and metabolic interference in yeast cells. These findings suggest that while moderate ash content may optimize ethanol production, higher ash levels negatively impact fermentation efficiency by altering yeast metabolism and reducing sugar availability.

Huang et al.^375^ developed a biorefining process for waste wheat straw (WWS) with a very high ash content (29.5%), incorporating prewashing and liquid hot water pretreatment (LHWP). Their study highlighted the importance of demineralization via prewashing in improving enzymatic hydrolysis and fermentation efficiency. They found that prewashing significantly increased glucose yields from 39.7% to 76.6% by removing minerals and alkaline compounds. A robust ethanol yield of 0.4 g/g-cellulose was achieved across 5% to 30% of solid loadings. After enzymatic hydrolysis, xylobiose and xylotriose yields improved to 53 mg/g-WWS and 20 mg/g-WWS, respectively. The process could produce 1 ton of ethanol and 614.8 kg of xylooligosaccharides (XOS) per 10.9 tons of raw WWS, demonstrating the potential for value-added coproducts. Similarly, He et al.^378^ investigated the effect of reducing ash content in corn stover. They found that lowering ash content from 9.6% to 5.0% improved hydrolysis yield from 43.3% to 71.0% and ethanol yield from 51.7% to 73.5% in dry dilute acid pretreatment at 67% solids loading. The reduced efficiency at higher ash levels was attributed to sulfuric acid neutralization by alkaline minerals, reducing the effectiveness of the enzymatic hydrolysis and fermentation steps.

Although removing certain elements may positively impact pretreatment and enzymatic hydrolysis, removing them entirely would negatively impact downstream fermentation unless replaced. The cations K^+^, Mg^2+^, Ca^2+^, and Zn^2+^ are essential for yeast function and fermentation performance.^382^ Mg^2+^ in particular tends to have a positive effect on ethanol fermentation by a variety of organisms and can compensate for ethanol inhibition due to increased leakage of magnesium from the plasma membrane and by reducing the inhibitory impact of other ions such as Cu^2+^. Although certain ions are essential for microbial growth, if at too high a concentration, such as excess Ca^2+^ due to overliming, they can inhibit growth and depress ethanol yields.^39^

Engineering microorganisms for mineral or osmotic stress tolerance with enhanced ion homeostasis mechanisms (e.g., overexpressing cation transporters and osmoprotective genes) can increase Na^+^, K^+^, and Cl^–^ tolerance.^383,384^ Adaptive fermentation systems, such as using controlled pH and ion-exchange resins or chelating agents during fermentation, may help trap excess minerals, preventing microbial inhibition. Furthermore, cofermentation with tolerant microbial consortia, such as extremophilic microbes like halotolerant yeast and thermophilic bacteria that naturally withstand high salt and mineral concentrations, may enhance sugar conversion efficiency.^383^ Future technology needs for mitigating the impact of minerals on the fermentation process are summarized in Figure 13B.

##### 5.3.2 Effect of Minerals in Anaerobic Digestion for Biogas Production

Anaerobic digestion (AD) is a biological process that converts organic matter into biogas, primarily composed of methane (CH_4_), through the metabolic activities of a consortium of microorganisms. Whether naturally present in the substrate or externally added, minerals are essential for facilitating enzymatic reactions, stabilizing microbial communities, and optimizing the biochemical pathways involved in biogas production.^362,385^ However, when present in excessive concentrations, minerals and heavy metals inhibit microbial activity, disrupt enzymatic processes, and reduce biogas yields.^386^

Minerals such as Fe, Ni, Co, and Mo play critical roles in AD by supporting the structural and functional integrity of enzymes necessary for methanogenesis and hydrogen (H_2_) production. Ni, for example, is an essential cofactor for enzymes such as methyl-coenzyme M reductase (MCR) and CO dehydrogenase, which catalyze key steps in CH_4_ production.^378^ Similarly, Mo is crucial in formate metabolism and is linked to H_2_ production.^385^ Studies have shown that supplementing low-Ni substrates with Ni^2+^ at concentrations of 0.2–0.5 mg/L can increase CH_4_ yields by 10–20%, while Mo supplementation at 0.1–0.5 mg/L enhances both CH_4_ and H_2_ production in mixed-feed AD systems.^362,375,386^ While trace minerals are beneficial at optimal concentrations, their excess can harm microbial communities and impede AD performance. High Ni concentrations (>5–10 mg/L), for instance, are toxic to methanogens, causing cell membrane damage and inhibiting enzymatic activity.^32,386^ Similarly, while iron­(III) oxide (Fe_2_O_3_) nanoparticles can improve methane production by 20–30% by facilitating electron transfer and enhancing microbial metabolism, excessively high Fe levels can result in the formation of insoluble precipitates, such as iron phosphates. These precipitates reduce the availability of P, an essential nutrient for microbial growth.^362,385^ Excessive Fe can also cause oxidative stress in anaerobic microorganisms, further inhibiting methanogenesis.^35,378^

The interactions between minerals and microorganisms become more complex when substrates naturally contain clay minerals like kaolinite or iron-coated kaolinite. For example, Liu et al.^386^ demonstrated that kaolinite releases soluble Al (Al^3+^) into the system, which is toxic to methanogens, disrupting their cells and interfering with key enzymes needed for CH_4_ production. Fe-coated kaolinite exacerbates this issue by diverting electrons away from methanogenesis. Methanogens use ferric iron (Fe^3+^) as an electron acceptor, reducing it to ferrous iron (Fe^2+^) instead of producing CH_4_ from CO_2_ and H_2_. This phenomenon, referred to as “electron diversion,” was particularly pronounced during early experimental stages, significantly reducing methanogenesis.^386^ Despite these challenges, minerals can facilitate beneficial interactions such as direct interspecies electron transfer (DIET), where materials like Fe or conductive nanoparticles enhance the electron flow between microbial communities.^362,378,385^ This interaction improves microbial metabolism, accelerates methanogenesis, and boosts CH_4_ and H_2_ production. For instance, the addition of Fe_2_O_3_ nanoparticles in controlled amounts has been shown to increase CH_4_ production by up to 30% in mixed cultures.^32,386^

Balancing mineral concentrations is crucial in maintaining the delicate equilibrium between microbial diversity and enzymatic activity in AD systems. While trace minerals like Fe, Ni, Co, and Mo support enzymatic pathways in CH_4_ and H_2_ production, their concentrations must be carefully regulated to avoid toxicity or precipitation effects.^35,385^ By promoting beneficial interactions such as DIET and mitigating the negative impacts of excessive minerals or heavy metals, biogas production can be enhanced to create more sustainable and efficient anaerobic digestion systems for bioenergy applications.^362,375^

#### 5.4 Future Directions

The role of minerals in the biochemical conversion of biomass presents both opportunities and challenges for biofuel and other value-added product production. Although they are essential for microbial activity and catalytic processes, excessive mineral content can cause inefficiencies and operational issues. A multifaceted approach combining advancements in biomass cultivation, pretreatment, enzymatic hydrolysis, anaerobic digestion, and system-wide process optimization will enhance biofuel yields and sustainability.

Optimizing biomass mineral composition during cultivation via soil management, controlled fertilization, and genetic engineering can reduce extensive pretreatment in biofuel production. Soil amendments like biochar, compost, gypsum, and lime regulate mineral availability, preventing the excessive uptake of silica, sodium, and heavy metals. Cover cropping and crop rotation with hyperaccumulators or nitrogen-fixing legumes deplete surplus minerals and enhance fertility. Targeted fertilization with precision nutrient application, slow-release fertilizers, and microbial biofertilizers supplies essential elements without excessive absorption of inhibitory minerals. Synthetic biology enables the creation of bioenergy crops with low mineral uptake and high carbohydrate content for conversion.

Postharvest pretreatment strategies, including plasma-assisted pretreatment (which uses ionized gases to enhance enzymatic access), electrochemical leaching (which extracts metal ions to reduce ash), and ionic liquids (which selectively remove minerals like silica and potassium), can effectively eliminate inhibitory minerals from biomass, preserving fermentable sugars. When pretreatment is not feasible, innovations in enzyme engineering offer practical solutions for enzymatic hydrolysis in high-ash environments. Examples include fusion proteins, which enhance resistance to mineral inhibition, and immobilized cellulases, which improve stability in mineral-rich biomass. These technologies enhance biomass pretreatment and hydrolysis efficiency, promoting sustainable bioproduct formation and reducing mineral issues.

Following pretreatment and enzymatic hydrolysis, fermentation is key to producing high titers. If reducing the high mineral concentration in biomass during pretreatment and/or enzymatic hydrolysis is not feasible, engineering microorganisms for stress tolerance through ion homeostasis or by overexpressing cation transporters and osmoprotective genes can be an economical approach to counteracting the high mineral content in biomass. Co-fermentation may also enhance yields, as extremophiles improve enzyme activity and tolerate ionic stress. Combining microbial engineering with process adaptation can boost the efficiency of various biomass feedstocks. Trace minerals play vital roles in enzymatic pathways involved in methane and hydrogen production, supporting key microbial enzymes like hydrogenases and methanogenesis-related cofactors. Strategies such as mineral supplementation optimization, bioaugmentation with DIET-capable microbes, and selective adsorption or precipitation control of excess metals can be employed to create more sustainable and efficient anaerobic digestion systems. These strategies ultimately enhance methane production and bioenergy recovery.

The practical viability of future biorefineries will require merging advanced science with industrial innovations and sustainable policies. A comprehensive strategy encompassing feedstock selection, pretreatment, biochemical conversion, and process optimization is vital for addressing mineral-related challenges.

### Section Summary

In this section, we have reviewed the role of minerals during biochemical biomass conversion and the challenges they pose in process optimization, especially the negative effects they can have on reducing enzymatic hydrolysis and fermentation efficiencies. We discussed the effects of minerals during the enzymatic and fermentative steps and their interactions with the biomass structural components through complexation, reducing process efficiency. We discussed how high mineral content in biomass negatively impacts enzymatic hydrolysis by altering the enzyme structure and reducing their catalytic activity. We summarized how minerals disrupt the internal cellular environment in fermentative microorganisms, causing osmotic stress and cell membrane damage, reducing product yields. Additionally, we discussed the role of minerals in the anaerobic digestion of biomass for biogas production, where minerals are essential for optimal biogas production, but an excess can be detrimental to the process. We have also discussed some potential solutions and areas of research needed to mitigate the impact of minerals during biochemical biomass conversion.

## 6 Inorganics as Catalyst Poisons

### Section Overview

In this section, we discuss the impact of inorganic elements present in biomass feedstocks and other elements, such as Cl, S, N, and P, on several conversion technologies, including catalytic fast pyrolysis (CFP), biosyngas reforming, and hydrodeoxygenation (HDO). The aim is to help understand the key issues caused by the presence of inorganics in the biomass feedstocks, including catalyst deactivation. Future research needs for developing technologies that can mitigate the negative effects of such inorganics are discussed.

Catalyst stability in biomass conversion is critical for maintaining high product yield and continuous operation in a conversion process. Over the years, researchers have investigated thermochemical conversion of biomass feedstocks to produce renewable fuels and chemicals. These technologies include CFP, gasification, and HDO. However, catalyst deactivation has been a prominent issue for these technologies due to the presence of inorganics in biomass feedstocks.^12^ A relevant example is the deactivation of fluid catalytic cracking (FCC) catalysts by impurities in petroleum feedstocks.^387,388^ FCC units are primarily used to upgrade vacuum gas oils (VGO) and residues (VGR) to produce gasoline, distillates, and important chemical building blocks such as propylene and ethylene.^389^ FCC feedstocks differ in inorganic content found in biomass samples. Table 11 compares typical contaminant levels in VGO and biomass feedstocks that have been reported in the literature.^390–399^ In some cases, raw crude oil can contain high amounts of Ni (up to 200 ppm), V (up to 2,000 ppm), and Ca (up to 120 ppm).^400^ Prior to fractionation, the raw crude oil goes through a desalter (i.e., washing step) to reduce the inorganic content, especially alkali and alkaline earth salts.^401,402^ AAEMs are the major contaminants in biomass feedstocks. Biomass feedstocks can contain an order of magnitude more AAEMs and other elements compared to FCC feedstocks, thus making it more challenging to catalytically convert biomass.

**Table 11:** Comparison of Inorganic Species Content between Conventional Crude Oil Feedstocks and Typical Biomass Feedstocks from Agricultural and Forest Residues.^a^

	Elemental concentration (ppm)										
**Feedstock**	S	N	P	Ni	V	Fe	K	Na	Mg	Ca	Si
***Petroleum***,^390^ ^b^											
Crude oil	500–39,000	100–3,000	–	0–55	0–145	0.9–9.0	–	1.0–6.5	–	–	–
Vacuum gas oil	1,200–35,000	300–1,700	–	0–1	0–1	0	0	0	–	–	–
Vacuum gas residue	2,000–67,000	1,000–7,600	–	1–150	0.2–400	5–30	0	0	–	–	–
***Agricultural residues***											
Corn stover^391–393^ ^e^	1,500–1,700	23,000	760–3,300	–	–	160–340	8,300–33,000	400	2,800–4,000	3,400–5,000	14,000
Wheat middlings^394–396^ ^e^	2,000	25,000–29,000 ^406^ ^d^	9,500–11,000	–	–	140–150	10,990–13,400	60–350	4,000–5,030	1,200–1,300	–
Soybean hulls^397,398^ ^e^	900–1,000	15,000–20,000 ^406^ ^d^	1,700–2,200	–	–	320–670	13,000–17,000	100–110	2,500	5,600–6,000	–
***Forest***											
Loblolly pine^399^ ^c^,^e^	80–130	–	140–260	–	–	430–1,200	1,200–2,300	130–350	400–730	1,200–2,200	2,600–6,600

a Units are on a mass basis for all calculations.

b Values were obtained from published ExxonMobil crude assays and calculated on a basis of 100 units of crude oil, where vacuum gas oil is the summation of vacuum cuts from 370–550 °C and residues are cuts above 550 °C.

c Values were calculated and normalized assuming 100 units of biomass based on elemental ash analysis.

d Nitrogen concentrations were estimated using a nitrogen to protein ratio of 6.25.

e In some references, the ash content oxide analysis of biomass fractions was used to normalize the ppm concentration of the original biomass; in others, the mass balance of the feedstocks was provided along with the elemental analysis; in others, the averages across feedstocks cultivated from various sources were reported.

In both crude petroleum and biomass feedstocks, inorganic contaminants needs to be either removed or managed prior to catalytic upgrading. These contaminants can deactivate catalysts via a variety of potential mechanisms. Some contaminants may poison catalysts by adsorbing on and occupying active sites that are essential to facilitate chemical reactions. Others may foul catalyst surfaces and pores which can physically block reactants from undergoing reaction. Some contaminants also promote unwanted reactions to form low-value byproducts. Depending on the mechanism and species, catalyst poisoning can be either irreversible or reversible. Inorganic elements such as P have both deleterious as well as promotional effects on the performance of zeolites depending on the reaction system.^403^ Certain transition metals have been used as dopants to promote the activity and/or selectivity of zeolites used for converting ethanol to hydrocarbons^404^ and catalysts used in hydrodeoxygenation of model bio-oil compounds.^405^ Model biomass substrates are relatively pure and do not contain inorganics typically found in biomass-derived substrates. Clearly, caution must be exercised when applying the results of studies performed with model substrates in the processing of real biomass.

Over time, contaminants in the biomass gradually accumulate on the catalyst and cause deactivation. In the case of FCC catalysts, the management of catalyst activity by deliberate withdrawal and replacement of small amounts of catalyst periodically results in the accumulation of contaminants to a pseudo steady-state concentration level. At that point, the catalyst activity remains stable, and the catalyst inventory is referred to as being “at equilibrium” (or “E-cat”), allowing the process to run uninterrupted for extended periods.^387,388^ This balancing of replacement rates and deactivation rates is strongly influenced by the contaminant level in the feed. Similarly, catalytic biomass conversion processes are also sensitive to contaminant-induced deactivation phenomenon. However, because of higher concentrations of contaminants in the feed, catalyst activity management in biomass conversion processes is more challenging compared to petroleum processes.

As shown in Table 11, biomass samples have a wider range and higher concentration of inorganics compared to conventional crude oil feedstocks. For example, petroleum feedstocks can contain trace amounts of AAEMs (≈7 ppm) whereas biomass feedstocks can have concentrations as high as 13,380–42,400 ppm in total AAEMs concentration. S, N, and P content in biomass feedstocks can also be anywhere from 2 to 3 orders of magnitude higher in concentration than crude oil feedstocks. Si is also present in biomass feedstocks due to the mechanisms of plant growth, whereas no detectable levels are present in crude oil feedstocks. Therefore, with biomass feedstocks, it becomes crucial to understand the transfer of inorganic materials from biomass to catalysts and establishing catalyst deactivation protocols and methods to provide cleaner feedstocks for biomass conversion.

#### 6.1 Catalytic Fast Pyrolysis Using Zeolite Catalysts

CFP of biomass feedstocks has been extensively reported in the literature for production of aromatics, including benzene, toluene, and xylene (i.e., BTX), and olefins such as ethylene and propylene.^33,407,408^ These molecules are currently utilized as feedstocks in the petrochemical industry to manufacture a wide range of everyday products, including plastics, synthetic fibers, detergents, and lightweight transportation materials. Zeolites have been the main choice for upgrading biomass via CFP due to providing (i) the highest BTX yields, (ii) deoxygenation chemistry, cracking, carbon–carbon coupling, and aromatization chemistry, (iii) shape-selectivity by the micropore channels within their structures to produce specific molecules based on molecular size,^409^ and (iv) good hydrothermal stability.^410^ The zeolite catalysts undergo temporary deactivation due to coking, which is burned off in a regenerator.^409^ At the lab scale, CFP chemistry has been extensively studied.^407,409,411^ Despite this, the effects of inorganic species in biomass on catalyst activity are not yet fully understood.

Inorganics such as K, Na, Mg, and Ca can poison zeolite catalysts by strongly binding to the active sites. The active site on a zeolite is a Brønsted acid site, resulting from a charge imbalance between silicon and aluminum atoms, as illustrated in Figure 14. A major deactivation route for zeolites is via ion exchange, which can permanently deactivate these Brønsted acid sites.^412,413^ The deactivation of Brønsted acid sites over zeolites is a function of the size and charge of the metal ion. In general, ions with larger radii and monovalent (+1) charge can exchange more readily than divalent ions (+2 charge). For inorganics that are typically prevalent in biomass feedstocks (Table 11), the tendency for ion exchange decreases as follows: K > Na > Mg > Ca.

**Figure 14:**
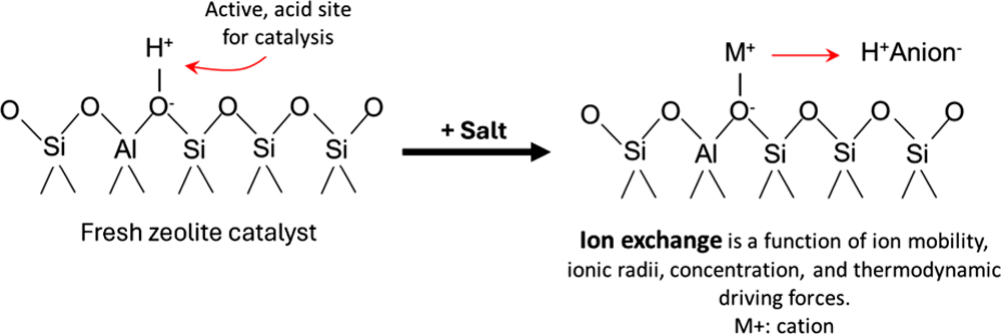
Ion-exchange mechanism of metals onto the surface of zeolites. H^+^ signifies the Brønsted acid site. M^+^ signifies the metal attacking Brønsted acid sites.

Zeolites can be negatively affected by water via hydrothermal dealumination (HDAL),^410^ causing the destruction of the zeolite framework by removing Al content and destabilizing the framework, resulting in a loss of activity. As a result of HDAL, the number of active Brønsted acid sites decreases. For feedstocks with high oxygen content, such as biomass, water will be produced during thermochemical conversion processes. Therefore, Brønsted acid sites can be affected by both HDAL and ion-exchange by AAEMs. Kalogiannis et al.^414^ suggested steaming the zeolite catalysts at around 800 °C prior to CFP to hydrothermally stabilize the catalyst and avoid HDAL issues at temperatures below 800 °C during regeneration processes, though this can result in reduced activity due to HDAL.^410^ This approach can also be used to decouple the effects of Inorganic accumulation and HDAL, as most of the loss in acidity can be attributed to the accumulation of AAEMs. As a future research challenge, decoupling these two effects by implementing a steam pretreatment protocol for HDAL could provide a better understanding of how metal accumulation affects catalytic activity.

Carlson et al.^33^ showed that after 10 regeneration cycles, the activity of the HZSM-5 catalyst permanently decreased due to the reduction of total acidity and the accumulation of AAEMs (i.e., Mg, K, Ca) on the catalyst. The activity loss was attributed to the loss of weak nonframework Lewis acid sites in the nonzeolite components of the catalyst, such as alumina, with only a slight change in the Lewis and Brønsted acid sites of the zeolite, even though the reactions lasted for about 5 h. The authors point out that at longer reaction times, metal accumulation will be a concern, similar to the gradual accumulation of and deactivation by V and Ni during crude-oil processing with FCC catalysts. Other studies^294,415^ have reported similar results, where the catalyst activity was permanently lost after 5–8 regeneration cycles. Yildiz et al.^294^ reported that after eight regeneration cycles, the catalyst activity was the same as that of conventional thermal pyrolysis. Niu et al.^415^ provide a similar trend, as the oxygenate yields obtained in CFP after 5 regeneration cycles were similar to those found in noncatalytic pyrolysis, suggesting a permanent decrease in the degree of deoxygenation afforded by the catalyst. In general, it has been found that ash/metal accumulation permanently deactivates zeolite catalysts,^294,415^ decreases the aromatic yields,^415,416^ and increases the yields of noncondensable gases^294^ and oxygenated species,^415,416^ suggesting a low degree of deoxygenation.

In many cases, experimental findings on inorganic/ash accumulation were conducted using *in situ* CFP, where biomass is directly fed into the reactor and the pyrolysis vapors react on the catalyst to form bio-oil in the same reactor. This approach can result in a high accumulation of inorganics, ash, and char on the catalyst prior to regeneration. Leijenhorst et al.^109^ conceptualized a simplified model depicting how inorganics in biomass can be transferred into the gas phase due to the presence of organic volatile compounds and char. During catalyst regeneration, the organic volatiles and char can be burned off and can either (i) leave behind inorganics present or (ii) volatilize inorganics and travel throughout the catalyst pores. In both cases, these two potential effects can hinder catalytic activity by interacting with acid sites on the surface and within the pores of zeolite catalysts.

Kalogiannis et al.^414^ performed *ex situ* pyrolysis in a cascade reactor system to provide direct evidence of the effects of inorganic accumulation on catalyst activity. The cascade involves a first reactor with inert beds where thermal pyrolysis is conducted to capture ash/inorganics from biomass. The gases produced in the first reactor then go to a second reactor packed with zeolite catalyst. The most important trends found were the following: (i) ash/inorganic accumulation in the inert bed in the first reactor promotes the conversion of vapors into solid products and decreases bio-oil production, (ii) CO_2_ yields increase as ash/inorganics accumulate in the first reactor, and (iii) the degree of deoxygenation increases as the amount of inorganics accumulated in the first reactor increases and not due to changes in activities or properties of the zeolite catalyst in the second reactor. The results were reproduced when the inert bed in the first reactor was replaced with a fresh one. For reliable control of bio-oil properties, it is essential to control the degree of catalytic deoxygenation by minimizing inorganic interference with the active sites. During *ex situ* CFP, the reduction in surface area of the zeolite was negligible, and Brønsted acidity was not significantly altered (10% decrease in *ex situ* CFP vs 64% decrease with *in situ* CFP despite higher biomass to catalyst ratios being used in *ex situ* CFP). Other techniques have shown similar results when contaminants were removed. Niu et al.^415^ reported decreases in surface area and micropore volume of a supported HZSM-5/Al_2_O_3_ catalyst after five regeneration cycles when pyrolyzing untreated algae feedstocks using *in situ* CFP. During the *in situ* CFP of washed (using 1 M citric acid to remove mineral content) algae feedstock, the surface area and micropore volume were fully restored compared to untreated algae that had a higher concentration of minerals. Their findings were also supported by energy-dispersive X-ray spectroscopy (EDS) mapping, which showed that demineralized samples resulted in minimal accumulation of minerals on the catalyst surface compared to using untreated feedstocks.^415^

Other sources of zeolite deactivation include nonmetal species such as N, P, S, and Cl. Nitrogen-containing molecules can affect acid sites and alter the structure of zeolite catalysts; however, nitrogen poisoning is generally reversible. Nonetheless, certain operating conditions can have an impact on catalyst activity and zeolite structure. For example, nitridation can occur at temperatures above 500 °C, which can alter Si–O–Si and Si–OH–Al bonds via oxygen substitution.^417,418^ The degree of nitridation also increases with reaction temperature and time.^419^ Nitrogen compounds can deactivate Brønsted acid sites by forming Si–NH_2_–Al bonds and can alter the structure of the zeolite forming Si–NH–Si bonds. At temperatures below 500 °C, it is possible to remove nitrogen-containing compounds from the zeolite framework via the Hoffmann elimination reaction^420^ given by eqs 4 and 5 where R is the hydrocarbon group associated with the nitrogen-containing compound.

4 $${\mathrm{HRNH}}_{2}+\mathrm{Si}-\mathrm{OH}-\mathrm{Al}\overset{K}{↔}{\mathrm{HRNH}}_{3}^{+}+\mathrm{Si}-\mathrm{Al}$$

5 $${\mathrm{HRNH}}_{3}^{+}+\mathrm{Si}-O^{-}\overset{k}{↔}{R+\mathrm{NH}}_{3}+\mathrm{Si}-\mathrm{OH}-\mathrm{Al}$$

As discussed in Section 3 and summarized in Tables 3–5, the typical concentrations of nitrogen in biomass feedstocks are highly variable and depend on the type and source of the biomass. It can be up to 6,000 ppm in woody biomass, up to 14,000 ppm in herbaceous biomass, and up to 12,300 ppm in agricultural residues. In some cases (Table 11), the nitrogen content can be even higher, such as in soybean hulls and wheat middlings, estimated at up to 20,000 ppm and up to 29,000 ppm, respectively, mainly present in the form of proteins and amino acids. These species may pose significant challenges for zeolites depending on the reaction conditions used. Typically, basic nitrogen compounds interact strongly with acid sites in zeolites, while inorganic nitrates do not typically pose significant issues as the anions do not interact with the zeolite. Therefore, one crucial factor to understand is the amount of basic nitrogen present in biomass. This nitrogen can typically be in the form of proteins containing amine (−NH_2_) groups^421^ that, once volatilized, can interact strongly with zeolite acid sites. Basic nitrogen containing species have been shown to affect industrial catalysts such as those used in FCC.^422^

Phosphorus compounds have similar effects as those seen with nitrogen compounds on zeolite catalysts. It is important to note that P has been used at the industrial scale to improve the hydrothermal stability of zeolites, which can be considered a “controlled” addition.^403,410^ Typically, the P content added is sufficient to minimize loss of acid sites while maximizing hydrothermal stability. In the case of biomass feedstocks, phosphorus minerals that interact with the zeolite can be considered an “uncontrolled” addition, which can have significant detrimental effects on catalytic activity. As shown in Table 11, the P content can vary from 140 to 11,000 ppm depending on the biomass feedstock. Therefore, the following discussion of P effects is centered on what can occur if the “uncontrolled” addition is significant.

P-containing compounds can influence the zeolite structure and affect Brønsted acidity. It has been shown that increasing the P/Al ratio with phosphorus content results in a reduction of Brønsted acid sites.^403,423^ Furthermore, one study found that the loss of acid sites is not due to physical blocking but rather a chemical transformation, which can indicate irreversible deactivation.^423^ P is also known to accelerate hydrolysis reactions, promoting Si–O–Al breakage in the presence of water to chemically alter the structure.^403^ It has also been reported that high loadings of P on zeolites can permanently remove Brønsted acid sites,^403,423^ decrease micropore volume and surface area,^403^ and thereby introduce diffusion barriers and alter shape selectivity.^424^ Most of the negative effects of P on zeolites occur during catalyst regeneration cycles and thermal treatment, such as catalyst steaming, where high temperature is used to either remove coke deposits or hydrothermally stabilize the zeolite. However, prior to catalyst regeneration, reversible deactivation of P can be achieved by washing with hot water.^403,425^ Therefore, before undergoing catalyst regeneration or other thermal treatments, it is recommended to water wash the catalyst to minimize P content and preserve activity and structure.

Chlorine in biomass samples can also be problematic for zeolite catalysts.^12^ Heating biomass can result in the production of volatile organic compounds containing Cl, which can negatively affect the activity of acidic zeolites, particularly in oxidative environments. Aranzabal et al.^426,427^ found that irreversible deactivation of Brønsted acid sites can occur with Cl^–^ containing compounds. For S compounds, deactivation can occur in the presence of hydrocarbon streams to produce hydrocarbon poly sulfides and pure sulfur (S_8_), causing blockage within the pores of metal-based zeolite catalysts. These polysulfide compounds can be reversibly removed by using H_2_ to break polysulfides back into H_2_S and light hydrocarbons at reaction temperature (380 °C).^428^

Coke formation is a major problem in CFP. The hydrocarbon pool that is responsible for producing aromatic and olefin molecules,^429^ also contributes to coke formation. The aromatic yield initially goes through a maximum before parallel coke buildup deactivates the catalyst after 40 min.^33^ Hence, in an industrial process, either continuous replenishment with fresh catalyst or regeneration of the coked catalyst in a separate vessel is required to address the coking problem. Various zeolite regeneration techniques have been proposed. Heracleous et al.^430^ proposed using an air regeneration temperature of 500 °C, as lower temperatures do not completely remove coke, and higher temperatures can cause dealumination as a result of water formation, destroying the Brønsted acid sites. Yung et al.^431^ reported that regeneration at 500 °C can almost fully regenerate zeolite catalysts, noting that economic trade-offs between regeneration temperature and kinetics will determine the optimum regeneration temperature and time.

At the industrial scale, companies such as Anellotech, Inc. have seen similar issues with coke, ash, and inorganic accumulation during CFP of biomass. Anellotech Inc. has implemented a catalyst regenerator/solids separator system, in which a pseudo steady-state activity is maintained by partially replenishing deactivated catalyst with fresh catalyst.^34,47^ Tolerable limits of AAEMs accumulation on the catalyst (0.1 to 0.2 wt % K) were established to mitigate catalyst deactivation.^34,47^ Figure 15 shows K accumulation as a function of reaction cycles in the case of unpretreated pine wood feedstock. A reaction cycle includes the total time duration of biomass reaction followed by catalyst regeneration post-reaction. Approximately 50 reaction cycles can be accommodated in a day. As shown in Figure 15, K accumulation on the catalyst increases linearly with the number of reaction cycles and exceeds the tolerable limit within a few cycles. This clearly emphasizes the importance of removing problematic elements from biomass to tolerate limits to sustain reaction cycles at an economically viable level.

**Figure 15:**
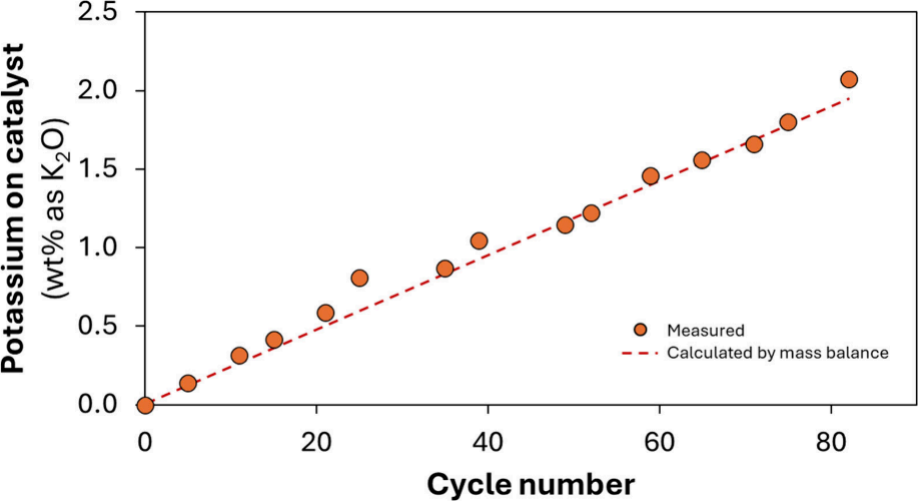
Potassium accumulation in the BioTCat CFP process, developed by Anellotec Inc., as a function of reaction cycles.

Anellotech Inc. further demonstrated that reducing the inorganic content in the feedstock by acid washing reduces the amount of coke and char produced during CFP, aligning with the results obtained by Kalogiannis et al.^414^ Another important issue at the industrial scale is how inorganic species can affect equipment functionality. Inorganics, ash, and halogens can accumulate on surfaces in pipes, heat exchangers, separators, and other pieces of equipment, causing adverse events such as slagging, fouling, and corrosion, leading to significant downtime, loss of performance, and increased maintenance costs. Compounds containing S and N can also corrode equipment and contaminate effluents, making the downstream purification of products difficult.^34,47^

As illustrated in Figure 16, reactant molecules diffuse into the micropores of the zeolites, where they undergo reaction on the active sites. As the reaction proceeds, the hydrocarbon pool also contributes to coking reactions. The gradual buildup of coke deposits causes pore clogging, limiting access to acid sites. The coke deposits, including biochar, can be burned off in an oxygen-rich environment, recovering catalytic activity. Accumulation of metals such as K and Ca will poison Brønsted acid sites, causing irreversible deactivation. Other species, such as N and P, can also adversely affect Brønsted acid sites and inhibit activity.

**Figure 16:**
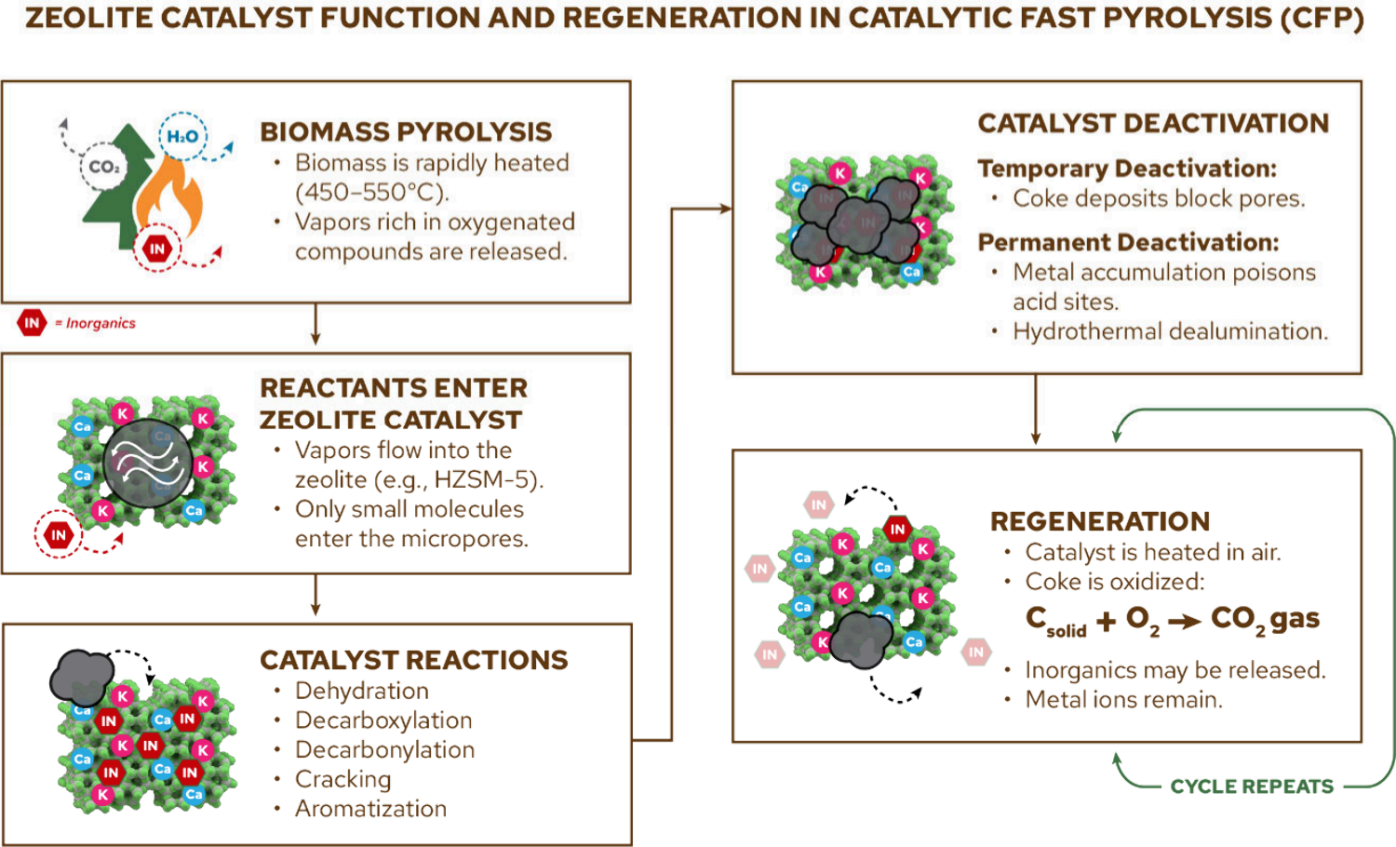
Schematic of reaction and deactivation mechanisms over zeolite catalysts during CFP of biomass.

Mild steaming of zeolites can improve hydrothermal stability and decouple the effects of HDAL and metal accumulation. Mild HDAL can also significantly mitigate the effects of water present in reaction streams as the deoxygenation of biomass leads to the formation of high levels of water present in the reaction system. This methodology would thus use an equilibrated catalyst for biomass conversion, further leading to more stable and prolonged reaction times. Removal of contaminants should be prioritized prior to feedstocks entering the catalytic system to mitigate the negative effects of inorganic accumulation and maintain control over desired reaction mechanisms. Table 12 thus summarizes the reported deactivation mechanisms found in the literature and presented in this section, specifically those triggered by the presence of inorganic species in biomass feedstocks.

**Table 12:** Key Deactivation Mechanisms of Major Inorganic Species in Biomass for Zeolite-Based Catalysts.

Contaminant species	Chemical deactivation by	Influenced by	Outcome
Inorganic elements	Ion-exchange, pore blockage, general fouling, structural damage	• Cation size	• Inner pore deactivation based on cation radii and charge equal to +1
		• Cation charge	• Surface deactivation of species based on cation charges larger than +1
		• Cation mobility	
Nitrogen	Inhibition of acid activity	• Reaction temperature	• Brønsted acid site destruction at temperatures >500 °C via nitridation
		• Reaction time	Si–OH–Al → Si–NH_2_–Al
		• Basic nitrogen concentration in the feed	• Reversible deactivation via Hoffman elimination
Phosphorus (uncontrolled addition)	Structural damage and diffusion barriers	• Concentration of P to Al during thermal treatment	• Permanent deactivation of Brønsted acid site activity at high loadings
			• Reversible deactivation if pretreated with washes prior to thermal treatment
Chlorine	Structural damage	• Reaction temperatures	• Irreversible deactivation of Brønsted acid sites

#### 6.2 Biomass Reforming

Biosyngas is the synthesis gas product made by the gasification of biomass feedstocks.^432^ Similar to other carbon-containing feedstocks, biosyngas can be produced by steam or autothermal reforming of biomass feedstock to produce streams with desirable H_2_/CO ratios for methanol synthesis or Fischer–Trøpsch synthetic fuels. Contaminants in the biomass feed can be carried into the syngas and influence the performance of steam reforming and downstream water–gas shift catalysts.^109^ Gas cleanup methods have been established^206,433^ to reduce contaminant levels. For downstream processes, contamination levels can be important. In the case of Fischer–Trøpsch, the tolerable limit for alkali metal is around 10 ppb.^206^ In the case of water–gas shift catalysts, Cl and S species can accumulate on the catalyst surfaces and deactivate them.^434^

Ni-based catalysts have primarily been utilized industrially for biomass steam reforming. They are commonly supported over refractory Al_2_O_3_, calcium aluminate, or magnesium aluminate. In some cases, noble metal catalysts have also been used in biogas reforming.^432^ One of the most concerning biomass contaminants for metal-based catalysts is the presence of S. Sulfur, a plant macronutrient, occurs in biomass at concentrations ranging from 0.01 to 0.8 wt %, depending on the type of biomass (Tables 3–5) and in some exceptional cases, it can be up to 2.5 wt %.^435,436^ The presence of S can pose major challenges during the catalytic conversion of lignocellulosic biomass. Depending on the metal catalyst and the reaction environment, S can strongly adsorb on the catalyst surface as either sulfide or sulfate, altering the geometry and electronic environment around the active metal site.^408,437–445^

In the case of Ni-based catalysts, S and Cl species are the main poisons since low amounts can cause catalyst deactivation.^446^ High-temperature regeneration using steam is needed to regenerate and reuse catalysts poisoned/deactivated by S adsorption.^447^ S can react with the Ni active site to produce NiS and NiSO_4_, and they can be removed by the following reactions.^446,448^

6 $$Nis+H_{2}O↔NiO+H_{2}S$$

7 $$NiSO_{4}+H_{2}↔NiO+H_{2}S+3H_{2}O$$

Bartholomew et al.^449,450^ and Lin et al.^413^ have provided detailed explanations on the deactivating effects of S on Ni-based catalysts. Catalyst deactivation occurs even at low S concentrations (tens of ppm) and is attributed to chemical adsorption and structural changes that occur on the metal surface. A single S atom can simultaneously interact with many Ni sites, as well as restructure the surface, decreasing the availability of active sites (i.e., Ni°).^413^ As shown in Tables 3–5 and 11, S concentrations in biomass feedstocks can be as high as 8,450 ppm in terrestrial biomasses and up to 69,000 ppm in aquatic biomasses, which can range from 2 to 3 orders of magnitude higher than the sulfide limit for Ni-based catalysts. Hence, substantial removal of S from the feedstocks is vital for the effective performance of syngas reforming catalysts.

Albertazzi et al.^446^ investigated the effects of using raw biosyngas produced from gasification to mimic realistic steam reforming conditions used in industry. Contaminants such as H_2_S and HCl cause a decrease in the specific surface area of Ni catalysts. Appari et al.^451^ showed that higher H_2_S content accelerates deactivation. Both Appari et al.^451^ and Albertazzi et al.^446^ showed that catalytic activity can be recovered via steam regeneration methods, followed by H_2_ reduction to recover the metallic species.

Hulteberg et al.^452^ showed that noble metals are more resistant to S poisoning compared to Ni, and that the poisoning resistance correlates with S-metal binding strength.^413,452^ Among the noble metals, Pd showed the highest resistance. Furthermore, noble metals may undergo deactivation by similar routes as Ni. In a study of single-stage catalytic gasification of lignin, similar sulfates and sulfides were found on Ru.^453^ Similar studies on the metal-binding strength of contaminants such as P, N, and Cl, on metal catalysts will be useful to assess their poisoning resistance and understand the deactivation mechanisms of these contaminants over metal-based catalysts to guide catalyst formulation.^450^

Ruthenium (Ru), Rhodium (Rh), and Iridium (Ir) metal catalysts have been investigated as biogas reforming,^432^ and biomass hydrodeoxygenation catalysts.^454,455^ As of 2025, the spot price of Ni is $16.0/kg.^456^ In comparison, the prices of Ru, Rh, and Ir are currently at $14,950/kg, $149,500/kg, and $141,000/kg, respectively.^456^ The justification for using these metals as catalysts includes better activity/product selectivity and greater resistance to deactivation,^432^ compared to Ni catalysts.^455^ It must be noted that during the ongoing energy transition, there will be competition between catalytic and energy metals that could drastically change the supply/demand dynamics for metals such as Ni and Co.^457^

While catalysts such as Ni/Al_2_O_3_–SiO_2_ catalysts perform better than Ru-based catalysts for lignin gasification, they are nevertheless susceptible to S poisoning.^458^ When lignosulfonates were gasified over NiO/CaAl_2_O_4_ catalyst to produce synthesis gas, hydrogen sulfide and carbonyl sulfide were formed as byproducts. The deposition of solid S compounds causes pore plugging.^459^ The gasification of biomass-derived syngas to methane over Ni catalysts results in multilayer S deposition on the catalyst surface, forming Ni sulfides at high temperatures.^460^ S species such as thiophene and hydrogen sulfide, present in the syngas feed, were implicated in the catalyst poisoning.^461–463^

Ash/inorganic accumulation is also an important deactivation route for biogas reforming catalysts. Albertazzi et al.^446^ found that between 40–50% of the specific surface area lost during biomass reforming was attributed to the accumulation of ash, soot, alkali, and carbon present. A drop in the metal surface area was also detected, which was attributed to the blocking of surface metal species and the sintering of metal particles. SEM images of spent catalyst showed that the surface was covered by smaller particles rich in alkali and transition metals as well as Si. These deposits can block both the pores and the active metal site. In the case of feedstocks rich in Si, such as landfill gas, siloxanes can decompose at reforming reaction temperatures, creating SiO_2_ deposits that cause pore narrowing and deactivate catalysts while also affecting equipment functionality.^464^

#### 6.3 Hydrodeoxygenation (HDO)

HDO is used extensively in industry to convert oxygen-containing fatty acid molecules into fully saturated paraffinic hydrocarbons, primarily for distillate fuel applications. It has been the subject of numerous academic studies to identify reaction mechanisms and catalytic effects. HDO has been extensively investigated for pretreating bio-oils to remove oxygen content from highly oxygenated bio-oils and other biomass-related feedstocks, such as vegetable oils. HDO involves a series of reactions, including hydrogenolysis, hydrogenation, decarbonylation, decarboxylation, and transalkylation. Transalkylation involves the transfer of methyl groups from methoxy groups in phenolic compounds to aromatic rings, which in return produce alkylphenols and alkylbenzenes.^465^ Unsaturated fatty acids contain one or more olefinic groups, and these carbon double-bonds are hydrogenated in parallel with oxygen-removal reactions. In the CFP processing of lignocellulosic biomass, some oxygenates are made including alcohols, ethers, and carboxylic acids. However, compared to fatty acids from animal and vegetable oils, the total concentration of oxygen in CFP products is lower.^429^ In HDO, supported metal catalysts are employed in the presence of pressurized H_2_ to remove O, S, and N heteroatoms, and saturate hydrocarbon molecules from substrates typically present in a liquid phase.^455^ Solvents such as short-chain alcohols (e.g., methanol, ethanol, propanol, butanol), formic acid, and water have been employed. These solvents can improve mass transfer and act as hydrogen donors, promoting an external source of H_2_ during the reaction.^466,467^ The primary aim of HDO is to increase the hydrogen-to-carbon ratio (H/C) and improve fuel stability and properties. HDO enhances the physicochemical properties of bio-oils, increasing their heating value while reducing acidity and viscosity.^468^ In academic research laboratories, HDO is typically carried out at temperatures between 200–400 °C and pressures of 70–200 bar, whereas industry and emerging HDO technologies have operating conditions for hydrotreating from 320–440 °C and 10–150 bar, depending on the feedstock used.^469–475^ In an ideal HDO process, oxygen in the bio-oil is converted to water without losing any carbon atoms. However, due to the complexity of the underlying reaction mechanisms, byproducts such as CO, CO_2_, and CH_4_ can also form.^346,348,465^ Although HDO can upgrade bio-oils and produce valuable chemicals, a major drawback is the high H_2_ pressure required. HDO technologies have implemented supported Ni and noble metals as catalysts,^455^ using supports similar to those used in biogas reforming.^455^ Noble metals have recently been used because they dissociate H_2_ effectively and have longer catalyst lifetimes compared to conventional hydrotreating catalysts for biomass derived feedstocks.^455^

Abdullah et al.^476^ showed the importance of cleaner bio-oils and the implications of inorganic accumulation on catalyst stability. Early hydrotreating technology could only be run for around 100 h on stream at 20 CV, where CV is defined as the number of catalyst bed volumes of bio-oil processed (for every unit of catalyst, only around 20 units of pyrolysis oil could be processed prior to regeneration).^476^ To avoid catalyst poisoning, an initial bio-oil cleaning protocol was established in which the inorganic content was reduced from 125.7 to around 30.5 ppm. The bio-oil was then stabilized by hydrotreating using a Ru/TiO_2_ catalyst that was regenerated at around 540 and 940 h TOS. The entire run was conducted for 1,200 h TOS, excluding regeneration time. After bio-oil pretreatment and stabilization using the Ru/TiO_2_ catalyst in the first catalytic step, 1,060 h TOS was achieved using a single charge (i.e., without the need for catalyst regeneration) of a sulfided CoMo catalyst for hydrocarbon production. This translated to 250 CV over 1,000 h, significantly improving catalyst stability and product output compared to previous HDO technology (i.e., 20 vs 250 CV). Clearly, for the development of new HDO technologies, it is important to understand how inorganic accumulation affects catalyst lifetime and product yields. It is also important to understand the configuration in which HDO is conducted. Fixed-bed reactors are commonly used, though trickle-bed reactors have been implemented. The patent literature is scarce on the dependence of metal ppm limits and reactor configuration, though a few key insights can be obtained. In the case of a trickle bed reactor, the feedstock has an established limit of around 1 ppm by weight or less of iron in its elemental form.^471^ In other cases, 10 ppm by weight of AAEM is desired, although 5 ppm or lower is preferred.^469^ It is evident that, in the case of HDO, low levels of contaminants are desired to avoid affecting catalytic activity, and further research should be undertaken to establish the lower limits observed at the industrial scale.

The literature on how metal/ash accumulation affects HDO catalysts is scarce. However, literature on conventional hydrotreating catalysts, such as NiMo/Al_2_O_3_ sulfided catalysts, provides some insights on how biomass contaminants may affect HDO catalysts. For sulfided catalysts, a S-containing compound needs to be continuously fed to maintain activity.^455^ For feedstocks with significant oxygen content (i.e., vegetable oil), HDO results in the formation of large amounts of water at the catalyst surface. The presence of water molecules and oxygen atoms can strip S from the catalyst. As a result, S compounds (i.e., H_2_S) are fed into an HDO reactor to counteract the stripping effect of water.^455^

In a recent study,^477^ the effects of contaminants such as Fe, P, and alkali metals were reported for a sulfided NiMo catalyst. The following trends were observed: (i) contaminants were mainly found near the surface layer (up to 100–200 μm depth) of the catalyst particle, (ii) surface area, pore volume, and size distribution were negatively affected, and (iii) deposition of contaminants on the catalyst particles contributes to pore blockage, exacerbating transport resistances and reducing catalyst effectiveness.^477^ Mortensen et al.^478^ investigated the effects of alkali metals and proposed that their accumulation permanently deactivates HDO catalysts by blocking active sites. While the effects of S-based compounds can be obscured in sulfided catalysts, their effects on nonsulfided HDO catalysts are scarce. An example of nonsulfided HDO catalysts is provided by Mortensen et al.^478^ Their studies showed that introduction of S can completely deactivate Ni-based HDO catalysts after 12 h of exposure.^479^ The general trends listed above also manifest in many recently reported HDO catalysts that use similar active metal species and supports including zeolites to impart an acid functionality.^455,480^

Another deactivation route involves the sintering of metal species on the catalyst surface, caused by weak metal–support interactions among other factors.^481^ Such detrimental effects can be amplified in the presence of inorganic contaminants as they cover the metal surface and possibly interact with active metal species. P-containing species can decompose into phosphoric acid, leading to the polymerization of carbon species on the catalyst. Indeed, this was observed with vegetable oils after 100 h of TOS, leading to severe plugging.^482^ Inorganics can also potentially interact with phosphates, leading to the formation of phosphate-based deposits. To prevent this from occurring, a degumming pretreatment step can be applied to remove phospholipids from the reaction stream.^483^

#### 6.4 Other Biomass Technologies

The following are additional examples of how inorganic contaminants can affect other biomass conversion systems.

In one example, woody biomass is pretreated with sodium sulfide and sodium hydroxide in the Kraft process to separate the lignin fraction. This results in a S concentration of up to 3 wt % in the resulting lignin.^484^ Ru/TiO_2_ catalysts, used for lignin gasification to CH_4_ and H_2_, are easily poisoned by the presence of S in the lignin feedstock. The S reacts with the Ru sites, forming ruthenium sulfide, ruthenium sulfite, and ruthenium sulfate species, all of which are inactive for lignin gasification.^485^ S impurities in the feedstock also deactivate the Ru/C catalyzed synthesis of 2,5-dimethylfuran or 2,5-dimethyltetrahydrofuran from 5-hydroxymethylfurfural.^486,487^

During HTL of kraft lignin, high sulfide concentrations in the feed resulted in decreased yields of aromatic monomers.^488^ When approximately 20 wt % of the S was removed from the kraft lignin using Soxhlet extraction with methanol, the overall monolignols yield increased during thermal depolymerization.^489^ Steam reforming of biomass tar has been reported over Ni, Rh, La_0.8_Sr_0.2_-Co_0.5_Ti_0.5_O_3_, and La_0.6_Sr_0.4_-Co_0.5_Ti_0.5_O_3_ catalysts. The presence of hydrogen sulfide in the tar causes catalyst poisoning by forming metal sulfides.^490–493^ During the hydrotreating of molasses over a Ni catalyst, the presence of organosulfur compounds in the feed poisoned the Ni catalyst, resulting in low yields of sugar alcohol.^494^

During hydrogenolysis of lignosulfonate to form monolignols, the Pd/C catalyst is poisoned by S adsorption. Similarly, Ru/Al_2_O_3_ catalysts used to hydrogenate biomass sugars to sugar alcohols are poisoned by S in the biomass feed.^495,496^ S poisoning was also the predominant cause of deactivation of Ru catalysts during hydrogenation of bio-oils.^497^ During Ni/C-catalyzed hydrogenolysis of biomass, hydrogen sulfide was formed as a byproduct.^498^ During HDO of phenol and anisole (as model lignin substrates) over a CoMo/Al_2_O_3_ catalyst, hydrogen sulfide deactivates the catalyst by forming sulfhydryl groups on CoMo.^499^ On Rh catalysts, hydrogen sulfide adsorbs strongly and blocks the active sites.^500^ The use of sulfuric acid as a catalyst during acetosolv fractionation of corn cobs increases the S content in the isolated lignin to around 2,900 ppm. During reductive catalytic depolymerization of the lignin to monolignols with a Pd/C catalyst using H_2_, the Pd sites were gradually poisoned by S. X-ray photoelectron spectroscopy (XPS) revealed that sulfide and sulfate groups formed on the surface Pd sites, rendering them inactive and amenable to possible leaching.^501^ The presence of S and Na in dealkaline lignin has been found to deactivate solid acid catalysts such as HZSM-23 during lignin depolymerization.^502,503^

Phosphorus also has the potential to affect catalytic activity by preventing the reduction of metals to the metallic active phase. One study showed that Co-based Fischer–Trøpsch synthesis (FTS) catalysts exhibited lower activity with increasing P content.^504^ This can be attributed to the strong interaction with oxide phases during regeneration, which prevents them from being reduced to the metallic form. N-containing compounds can also deactivate noble metals, with poisoning occurring due to the presence of the N–R groups.^505^ Halides such as Cl can interact with metallic Ni to form Ni–Cl species that promote sintering.^506^ XPS showed the existence of NiCl_2_ surface species. When HCl is removed from the stream, the presence of Cl is no longer detected.

Beyond S- and P-containing species, significant levels of inorganic salts and ash (such as Na, Ca, Mg, and Si), leftover carbohydrates, extractives, and trace metal ions from pulping and recovery procedures are frequently found in these feedstocks.^507–509^ These components may cause sintering, pore blockage, catalyst poisoning or alterations in the acidity or basicity of the surface, all of which can affect catalyst activity, selectivity and lifetime.^509^ Catalyst performance is also affected by variations in the concentrations of these elements across various lignin sources.^507^ These challenges underscore the importance of feedstock demineralization for robust catalyst design that is less influenced by the inorganics in the biomass feedstock.^508,510,511^

### Section Summary

In this section, we discussed the challenges that impurities in biomass pose to catalyst activity and lifetime. It is clear from the reviewed literature that it is important to understand how inorganics inherently present in biomass interact with various catalytic systems used to process the biomass. Due to the wide range of deleterious effects that biomass feed impurities can cause in different catalytic systems, it is recommended to establish inorganic removal strategies for biomass feedstocks prior to their catalytic processing. Such cleaning technologies should significantly remove AAEMs and inorganics such as S, P, Cl, and N. These pretreatment procedures should not only shed insights into the effects of the various elements on catalyst activity but also ultimately lead to rational strategies for improving overall catalyst performance and promoting practical viability of biorefineries.

In Table 13, we have summarized the elements in biomass that cause catalyst deactivation in various reactions, including AAEMs, silica species, P, S, Cl, and a few transition metals. The type and severity of catalyst deactivation across various elemental concentrations are also reported.

**Table 13:** Typical Concentration Ranges of Common Residual Ash Elements Associated with Measurable Catalyst Deactivation.^449,475,512–517^

Ash element/species	Typical form in biomass/feeds	Reforming catalysts (Ni-, Pt-, Rh-based)	Zeolites (acid catalysts, FCC)	Hydrotreating catalysts (CoMo, NiMo)	Primary mode of deactivation
**Alkali metals (K, Na)**	Salts, oxides, carbonates	1–10 ppm onset; >10–50 ppm rapid	10–100 ppm: measurable acidity loss; >100 ppm: severe	10–50 ppm: onset; 50–100 ppm: severe	Acid site neutralization, sintering
**Alkaline earths (Ca, Mg)**	Oxides, carbonates	10–50 ppm onset	50–200 ppm: gradual deactivation	50–200 ppm: pore blocking	Pore and site blockage
**Silicon (volatile siloxanes, silica)**	Silicon dioxide, organosiloxanes	1–10 ppm: severe	>100–500 ppm: framework damage	10–50 ppm: pore plugging	Surface deposition, site and pore blockage
**Phosphorus (P)**	Phosphates, organophosphates	1–5 ppm: strong poisoning	1–10 ppm: acidity loss	5–20 ppm: rapid	Strong chemisorption, site masking
**Sulfur (S)**	Hydrogen sulfide, organosulfur	<1–5 ppm for noble metals	Tolerant up to 100–500 ppm	Designed for 0.5–3 wt.% sulfur	Metal sulfide formation
**Chlorine (Cl)**	Hydrochloric acid, salts	1–10 ppm: causes poisoning	10–50 ppm: framework damage	10–100 ppm: activity loss	Corrosion, metal volatilization
**Iron (Fe), Nickel (Ni)**	Oxides, particulates	>10–50 ppm: causes coking	>100 ppm: fouling	>50–200 ppm: metal deposition	Fouling, coke promotion
**Zinc (Zn)**	Salts, oxides	1–5 ppm: severe	10–50 ppm: site blockage	5–20 ppm: strong	Alloying, irreversible poisoning

## 7 Inorganics Removal from Biomass

### Section overview

In this section, we discuss some of the more promising methods for removing inorganics from lignocellulosic biomass feedstocks. We summarize the benefits of torrefaction as a thermochemical pretreatment that can help improve inorganics removal by breaking down the biomass cell structure, air classification as a method to reduce the mineral content in biomass, and chemical leaching which can significantly reduce the inorganic content in biomass feedstocks. Additionally, we discuss the fundamentals of solubility, thermodynamic, kinetic aspects associated with mineral removal from biomass and how such an understanding can aid the rational development of removal technologies.

Figure 17 summarizes the various approaches to remove the inorganics from lignocellulosic biomasses.

**Figure 17:**
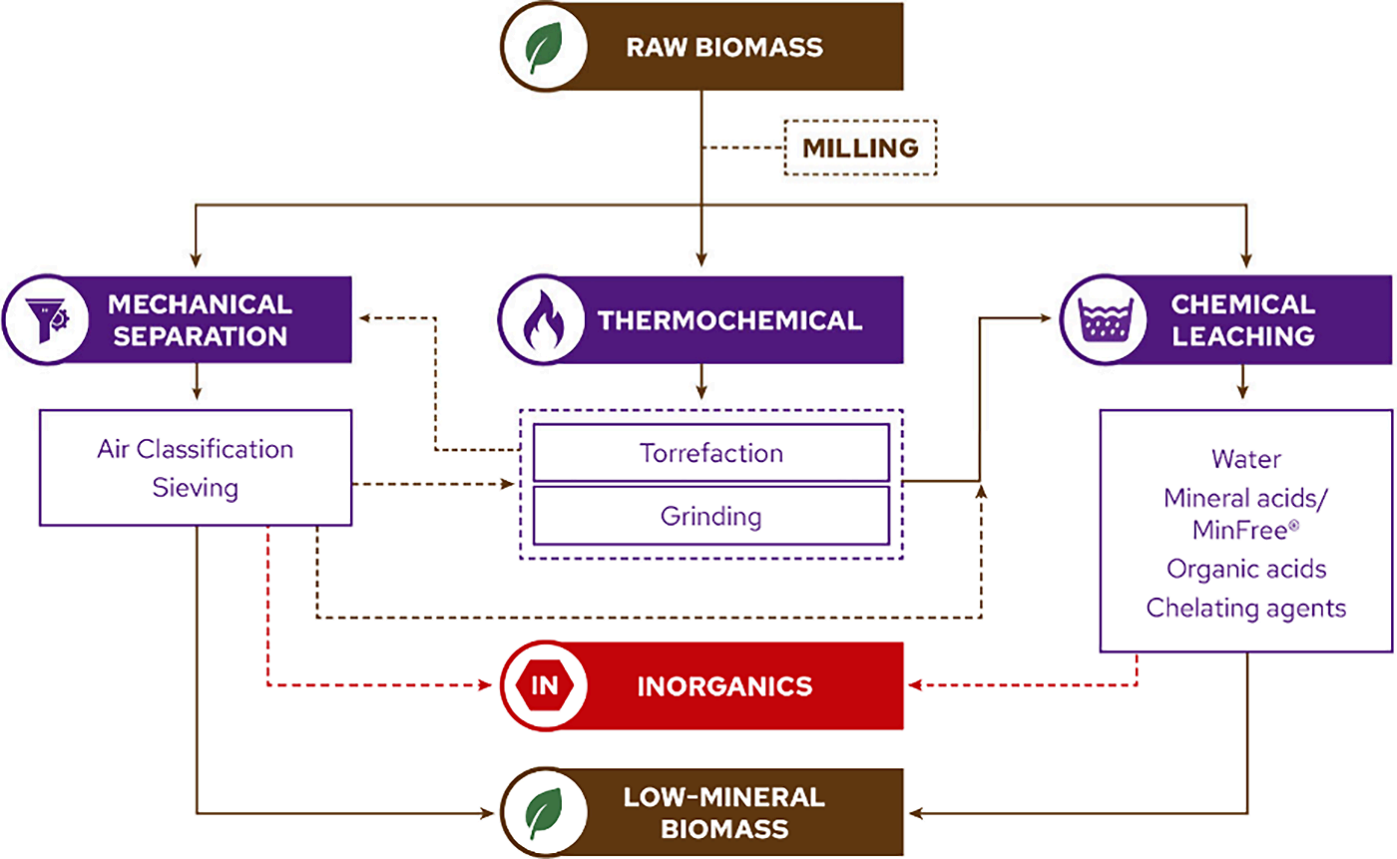
Possible pretreatment approaches to remove minerals/inorganics from biomass. Dashed lines indicate additional pretreatment steps to ease removal via the main pathway shown for mineral removal.

#### 7.1 Biomass Preconditioning: The Potential of Torrefaction in Biomass Demineralization, Grinding, and Sifting

##### 7.1.1 Torrefaction Pretreatment for Biomass Quality Improvement

Torrefaction^518^ is an airless thermal degradation process occurring between 200 to 320 °C that is used as a heat-pretreatment to improve biomass quality for combustion and gasification. Torrefaction can improve biomass grindability, reduce its hydrophilic nature, increase the heating value, and improve resistance to biodegradation.^104^ During torrefaction, biomass undergoes cell and tissue changes in the temperature range of 50–150 °C (called the nonreactive drying zone) due to moisture loss, generating structural changes such as cell wall shrinkage and a decrease in pore volume.^519,520^ The three main components of biomass (hemicellulose, cellulose, and lignin) decompose at different temperatures (Figure 18).^520,521^ In the first decomposition stage, between 150 and 200 °C (called the reactive drying zone), water and CO_2_ are removed through dehydration and decarboxylation along with the removal of some light volatiles. Between 180 and 270 °C the reaction is more exothermic, and hemicellulose undergoes depolymerization (200–215 °C), deacetylation (215–245 °C), and degradation (>245 °C). Amorphous cellulose decomposes at temperatures around 200 °C, while crystalline cellulose depolymerizes and decomposes at temperatures higher than 270 °C, typically between 240 and 350 °C, resulting in anhydrous cellulose and levoglucosan. Lignin degrades at a wide range of temperatures, from 250 to 500 °C. At 280 °C, it degrades to phenols due to cleavage of ether bonds. At temperatures higher than 280 °C, the reactions are more exothermic, increasing emissions of CO, phenols, cresols, and other heavier products. When the torrefaction temperature reaches approximately 290 °C, the three lignocellulosic biomass components degrade at a similar rate. Torrefaction temperatures above 300 °C give rise to the degradation of cellulose and lignin and are usually considered part of the pyrolysis process.^518–520,522^

**Figure 18:**
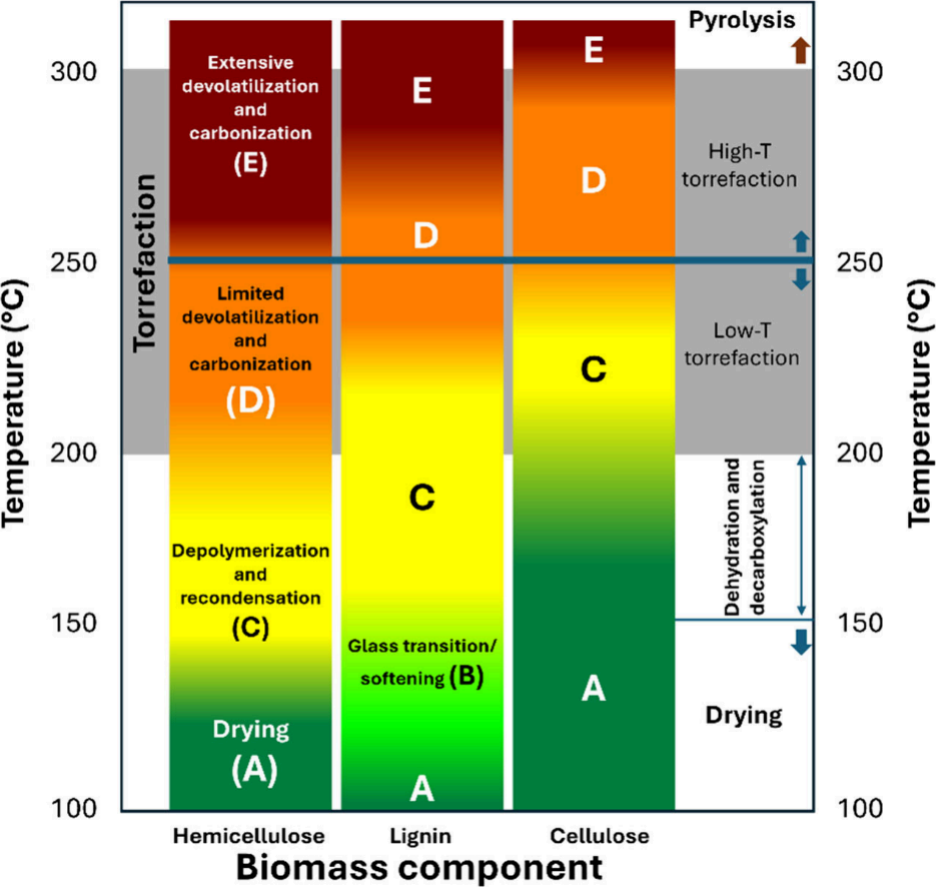
Decomposition regimes of lignocellulosic components during torrefaction. Adapted and edited from Bergman et al.^521^ Not subject to U.S. copyright.

Biomass torrefaction produces three main components: torrefied biomass, liquids (water, acetic acid, aldehydes, alcohols, furans, phenols, and ketones), and noncondensable gases (CO_2_, CO, CH_4_).^522–524^ The solid product yields of torrefied biomass from beech, willow, larch, and straw have been reported in the range of 95 wt % (5 wt % mass loss) at 230 °C to 80 wt % (20 wt % mass loss) at 270 °C with a high dependence on the biomass type and residence time. The liquid yields could range from as low as 2 wt % (230 °C) to 28 wt % (300 °C), with the products (Table 14) comprising water (4–13 wt %), acetic acid (1–5 wt %), and methanol (0.5–4 wt %). The noncondensable gas yields can range from 2 wt % to around 5 wt % when temperatures increase from 230 °C (50 min) to 300 °C (10 min), with CO_2_ (0.5–4 wt %) and CO (0–1 wt %) as the primary components.^519,522,524^

**Table 14:** Liquid and Gaseous Product Yields during Biomass Torrefaction at Temperatures from 230 °C (50 min) to 300 °C (10 min). Biomass: Willow, Larch, and Straw.^519,522,524^

Component	Yield (wt %)
*Liquid products*	
Water	4–13
Acetic acid	1–5
Formic acid	0.5–2
Methanol	0.5–4
Lactic acid	0.5–3
Furfural	<0.5
Hydroxy acetone	<1
Phenol	Traces
*Noncondensable gas products*	
CO	0–1
CO_2_	0.5–4

Torrefaction has been primarily developed as a biomass pretreatment technique that converts biomass into an easily grindable hydrophobic material. During torrefaction, dehydration, dihydroxylation, and tar condensation are responsible for biomass transformation from highly hygroscopic into hydrophobic char.^518^ Torrefied wood pellets showed a reduction in moisture uptake from 16 wt % of nontorrefied wood to 14 wt % when torrefied at 250 °C, and to 12 wt % after torrefaction at 300 °C.^519^ These characteristics allow torrefied biomass storage for a longer time without biological degradation. Torrefied biomass has enhanced grindability, enabling it to be used in entrained-flow coal power plants. Torrefied biomass has also been used in other applications, including gasification, pyrolysis, and metallurgical applications, as a potential replacement for coal to produce metallurgical coke.^519,525^

##### 7.1.2 Effect of Inorganics on the Torrefaction Process

Only a few authors^104,187,289,526,527^ have reported the effect of inorganics in the torrefaction process, and most of the reports focus on the impact of K. Khazraie S. et al.^104^ studied the effects of K, Na, Ca, and Mg on spruce and pine torrefaction by impregnating them with a nitrate solution of each metal. The results showed that K, Na, and Mg bound to organic sites increase the mass loss during torrefaction between 240 and 280 °C, with K having the most significant effect. Ca addition did not influence the mass loss rate during torrefaction. Spruce wood impregnated with 1.2 wt % of K showed a mass loss of 16.2% at 240 °C for 30 min, while the nondoped biomass had a mass loss of 7.6% under the same torrefaction conditions.

Barta-Rajnai et al.^526^ studied the effect of inorganic content during torrefaction of black locust wood, wheat straw, and rape straw, in the range of 200 to 300 °C for 1 h. They found from TGA studies of washed and unwashed samples that the removal of alkali ions by water washing at 60 °C caused a shift of the maximal decomposition rate of cellulose and hemicellulose to a higher temperature. They concluded that the thermal stability of cellulose in the washed locust wood was raised by 30 °C and by 50 °C in the washed straw sample compared to the unwashed samples due to the removal of the alkali ions, thus confirming the catalytic effect of alkali ions on cellulose decomposition. Additionally, the TGA of unwashed straw and torrefied at 300 °C for 1 h indicated almost complete decomposition of cellulose. In contrast, under the same torrefaction conditions, the cellulose content in washed straw was only slightly affected. They also concluded that the catalytic effect of inorganic elements is more pronounced at higher torrefaction temperatures. Saddawi et al.^187^ reported the torrefaction of washed and unwashed short-rotational coppiced willow, eucalyptus, miscanthus, and wheat straw in both chipped (1–4 mm) and chopped (<1 mm) forms. For example, the washed wheat straw and miscanthus showed a 15–18% increase in the dry torrefied mass yield compared to the unwashed biomass. It was attributed to the removal of key catalytic metal species (e.g., Na and K), resulting in slower reaction rates. Richa et al.^289,527^ have reported similar catalytic effects of K during the torrefaction of beech wood, hemicellulose, cellulose, and lignin. They impregnated the biomass samples with 0.004, 0.008, and 0.012 M K_2_CO_3_ and found that, at 250 °C, the catalytic effect was weaker compared to 300 °C torrefaction (105 min and 65% mass loss). The catalytic effect of K was more pronounced over hemicellulose and cellulose, causing a shift in the maximal degradation rate of cellulose by 11–14 °C when the K concentration was 0.012 M.

##### 7.1.3 Biomass Grinding Energy and Torrefaction

Grinding biomass reduces particle size and increases the surface area. It also exposes the inner parts of the biomass for more effective washing, facilitates a more uniform reaction, and enhances the overall efficacy of subsequent processing steps.^528^ The energy required to break biomass into smaller particle size comes from the deformation before fracture and can be modeled using the Rittinger law^529^ as defined by eq 8, where E is the grinding energy per weight, *C*_*R*_ is the Rittinger coefficient, and *d*_*p*_ and *d*_*f*_ are the diameters of the product and feed grind particles, respectively.

8 $$E=C_{R}\left(\frac{1}{d_{p}}-\frac{1}{d_{f}}\right)$$

The equation has been empirically validated for wheat straw, corn stover, switchgrass, miscanthus, and canola straw within the range of 0.43 mm to 2 mm.^529,530^ The grinding energy requirement is inversely proportional to particle diameter, which dictates the fineness of the ground product subject to economic and processing considerations. Torrefied biomass requires only 10–20% of the grinding energy needed for nontorrefied biomass.^531^ For example, torrefying pine chips or logging residues at 300 °C for 30 min reduces the grinding energy from 250 kWh/ton to 25–50 kWh/ton.^532^ Figure 19 shows the grinding energy for raw and torrefied beech and spruce biomass at different torrefaction conditions. It shows that the grinding energy of torrefied (280 °C, 28.1 wt % mass loss) beech is 90% less than the grinding energy for the nontorrefied biomass, reducing it from 850 to 90 kWh/ton.^533^ A similar reduction in grinding energy was reported, from 274 kWh/ton for nontorrefied sawdust to 84 and 43.6 kWh/ton for the torrefied sawdust with 15–25 wt % and 40–50 wt % mass loss, respectively. This is comparable to the grinding energy (45.1 kWh/ton) required by powder river basin coal,^534^ which serves as a reference. Similar reduction in grinding energy has been reported by other authors.^533,535,536^

**Figure 19:**
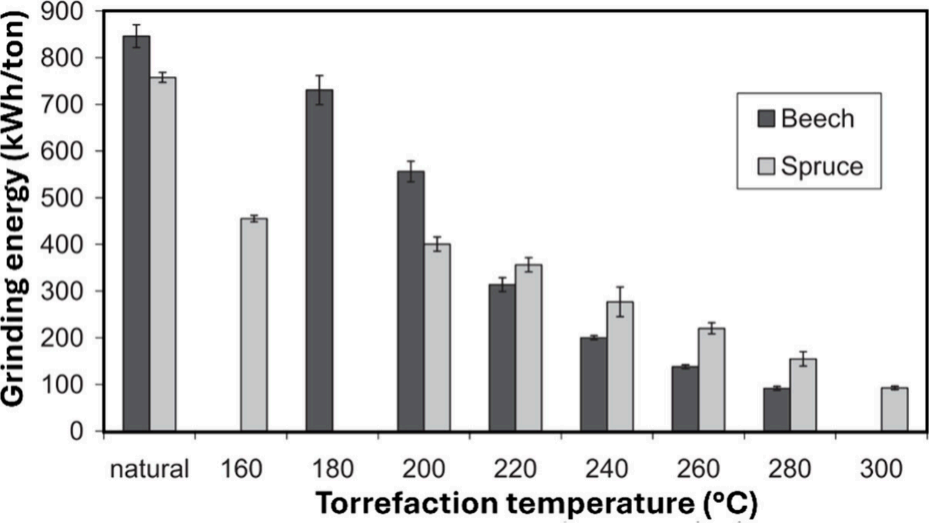
Grinding energy requirements for raw and torrefied beech and spruce as a function of torrefied temperature. Reprinted with permission from Repellin et al.^533^ Copyright 2010, Elsevier.

Another reason for grinding the biomass in the mineral removal process is the uneven distribution of minerals across different particle sizes. The ash content is a function of the biomass particle sizes after torrefaction and sieving. The ash content decreases with an increased biomass particle size.^410^ For example, switchgrass with a particle size of less than 0.09 mm has an ash content of 6.9%, while the fraction with a particle size greater than 0.09 mm has only 3.1%.^537^ Similar correlations between ash content and particle size have been reported for short-rotation forestry,^538^ pine bark,^539^ straw,^540^ and canarygrass.^537^

##### 7.1.4 Torrefaction Potential to Enhance Inorganics Removal

Previous studies combining acid leaching, torrefaction, and grinding generally followed the order acid leaching-torrefaction-grinding,^118,119,135,541–547^ or torrefaction-acid leaching-grinding.^548,549^ However, few of these approaches have explored torrefaction-grinding-acid leaching, despite its potential to enhance inorganic removal efficiency.^550^ Matyáš et al.^551^ revealed that during torrefaction (270 °C for 30 min) of *Arundo donax* biomass, elements such as K, Cl, and S form amorphous or crystallized clusters of KCl and K_2_SO_4_ on the outside of the particle surfaces, with a size of 1–100 μm. Water leaching at room temperature showed a slightly better removal efficiency from dried biomass (≈98% of K and Cl, and 75% of S) compared to torrefied biomass (≈90% of K and Cl, and 70% of S). Donepudi^534^ demonstrated that when biomass undergoes torrefaction with a 7% mass loss, combining it with high-shear stirring (5,000 rpm for 5 min) during the leaching process can help remove those mineral-rich clusters. This approach enhances ash removal of ground *Arundo donax* (<0.43 mm) by 40% compared to leaching without prior torrefaction. Bar-Ziv et al. have studied the effect of torrefaction on Cl removal^552–556^ and demineralization^534^ of both biomass and other wastes, such as municipal solid waste (MSW). They demonstrated that torrefaction (300–400 °C) can effectively remove organically bound chlorine from polyvinyl chloride and mixed plastic wastes, as well as from MSW and biomass blends. Residual inorganic Cl and other inorganics can be removed by mechanical and chemical methods.

Saleh et al.^557^ reported the release of Cl and S from two herbaceous biomasses (wheat straw and miscanthus) and four woody biomasses (spruce chips, spruce bark, waste wood, and short rotation coppice poplar) under torrefaction conditions at 250 and 350 °C. At 350 °C, approximately 64% of Cl was released from straw, over 80% from woody biomasses, 50–60% of S from straw and miscanthus, and 40–70% of S from woody biomass. In contrast, at 250 °C, only about 20% of the Cl from straw and 20–50% of S from woody biomass was released. Methyl chloride (CH_3_Cl) was identified as the primary chlorine compound released during torrefaction. Similar results were reported by Khazraie S. et al.^558^ in their study on birch wood, where approximately 85% of Cl and 50% of S were released during torrefaction at 280 °C.

Khazraie S. et al.^558^ also reported changes in the solubility of various inorganic elements during torrefaction. Elements such as Ca, Mg, and Mn transitioned from being organically bound and soluble in ammonium acetate to becoming more acid-soluble. The amount of acid-soluble P also increased after torrefaction at 280 °C. Potassium, which was primarily water-soluble before torrefaction, became slightly soluble in ammonium acetate and acidic solutions. These changes in solubility are attributed to several reactions involving biomass structural components and inorganic species during torrefaction, including (i) thermal degradation altering the carboxylic group content, (ii) reactions between KCl and organic functional groups (e.g., carboxylic and phenolic groups), leading to the release of HCl and the formation of organically bound K, and (iii) alterations in the amount of water-soluble organic compounds.^558^ Further research is needed to fully understand the transformations minerals undergo during torrefaction and their implications for solubility in different solvents.

While numerous reviews^518,525,531,559–561^ address the use of torrefaction in biomass processing, there is little understanding on the role of torrefaction in biomass demineralization. Here, we hypothesize the beneficial role that torrefaction could play in removing inorganics from biomass. A combination of pretreatments, such as torrefaction followed by washing, can help reduce the AAEMs, as well as S and Cl in the feedstock, thereby enhancing downstream gasification and pyrolysis processes. As reported elsewhere, such inorganics reduction results in a better quality bio-oil characterized by reduced water, acids, and oxygenates contents.^135,187,534,541,548^

The following findings motivate the further investigation of torrefaction for biomass demineralization:

1. During torrefaction, inorganics within the biomass form microcrystallites within the structure of torrefied material.^551^ Salt (e.g., KCl and K_2_SO_4_) deposits are formed due to the condensation of volatile species (e.g., K, Cl, and S), leading to the separation between the minerals and the organic structure. Matyáš et al.^551^ and Donepudi^534^ reported SEM and EDS mapping results confirming micron-size mineral (2–20 μm) particles entirely detached from the biomass structure. Those particles contained a variety of elements, including Na, K, Si, Mg, Ca, S, Cl, and Al.
  Following grinding of the torrefied material, the salt deposits separate from the biomass structure. The detached minerals are divided into two groups: (i) water-soluble (potassium and sodium minerals, and nitrates), and (ii) nonwater-soluble (silicates, carbonates, etc.). Biomass torrefaction thus enables the dissolution of water-soluble minerals at orders of magnitude faster rates than from raw biomass. For example, the potassium can be almost completely removed in under 3 min.^534^
2. Matyáš et al.^551^ and Donepudi^534^ also concluded that the mineral microcrystallite size can be controlled, which may partially enable separation between the minerals and the torrefied biomass. Figure 20 represents the three types of particles in ground biomass: organic material (black), minerals (blue hexagons), and organic particles with attached minerals (black with blue spots). This distribution is somewhat analogous to that of coal. The extent of grinding can control the size of the organic particles, while the size of the mineral particles remains almost constant with pulverizing time.

**Figure 20:**
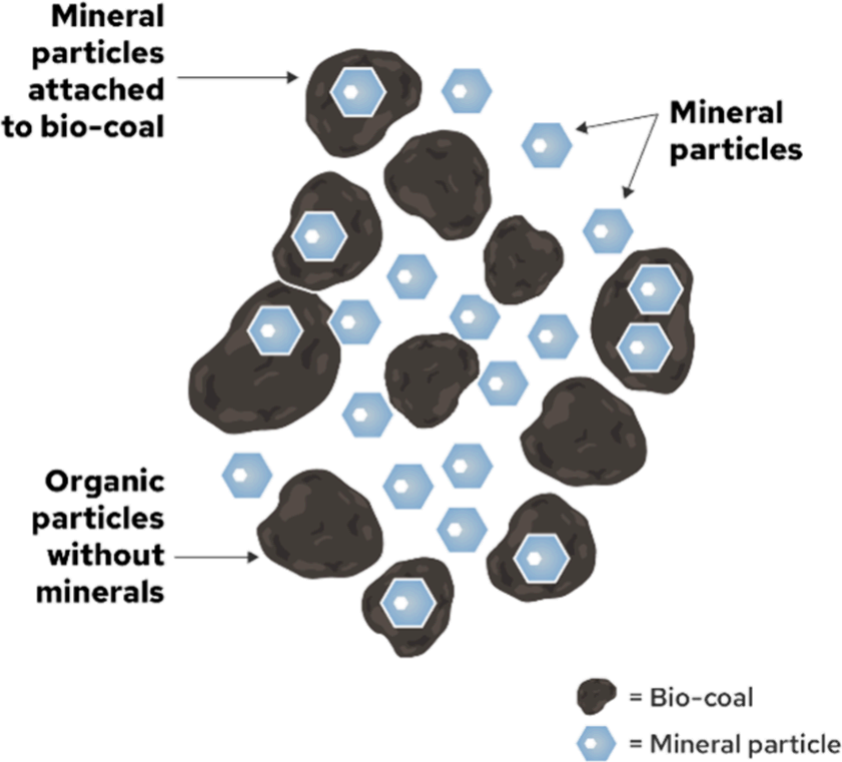
Representation of the three types of particles resulting from pulverizing a torrefied biomass.

#### 7.2 Mechanical Removal of Minerals from Biomass

Air classification and size separation are common techniques used to cost-effectively manage biomass feedstock quality. These methods utilize the physical property variations inherent in a given biomass to achieve targeted separation by fractionation. Air classification is a mechanical separation technique that harnesses differences in particle shape, size, and density to separate materials into distinct fractions. A diagram illustrating the air classification process is shown in Figure 21. This process involves using an air stream to fluidize and classify particles based on their aerodynamic properties. Lighter particles are carried away by the air stream, while heavier particles remain fluidized and are collected. Size separation involves using sieves or screens to separate particles based on their size. This method can be particularly effective when coupled with air classification, as it allows for the removal of finer particles that may contain higher concentrations of ash or other undesirable components.

**Figure 21:**
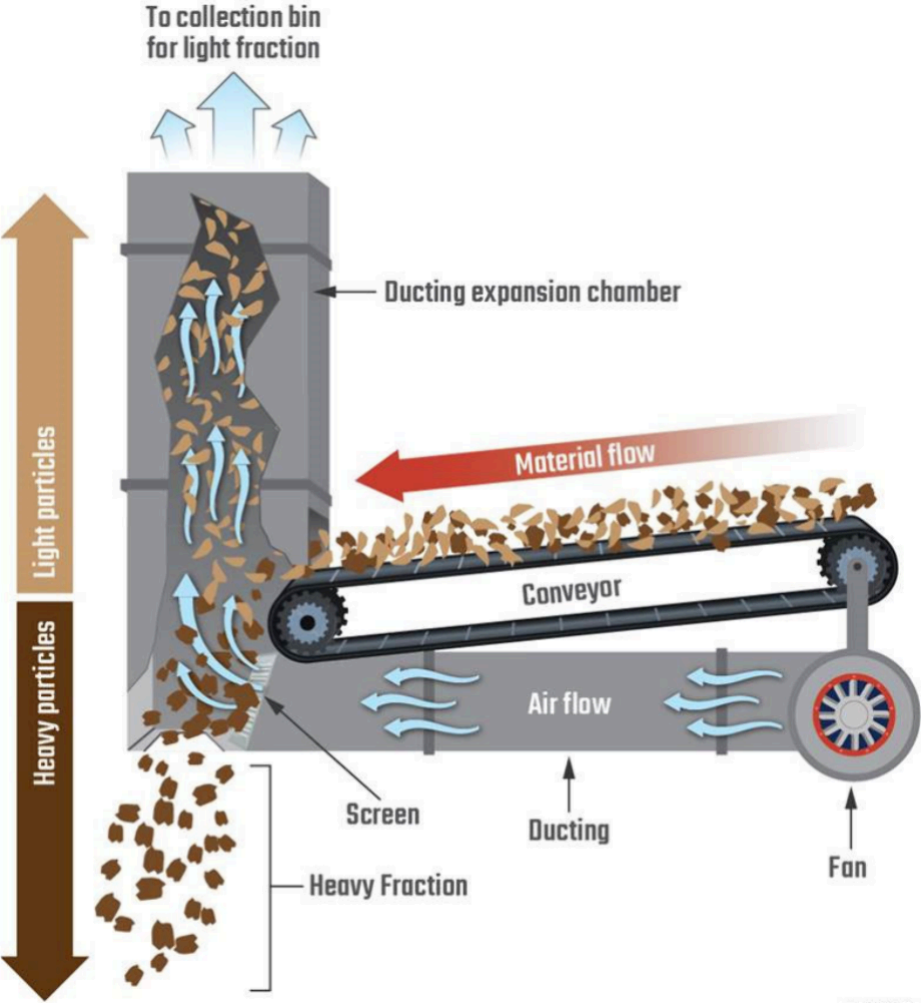
An illustration of air-classification separation of particles with differing aerodynamic properties. Lighter particles are entrained in the air stream and separated from the heavier particles.

By removing fine particles and dust that typically contain higher ash content, air classification produces cleaner biomass feedstocks.^7,399,562,563^ Such cleaner, low-ash biomass feedstocks result in higher conversion efficiencies, leading to better-quality end products and fewer operational problems.^21,24,25,27^ Air classification is a cost-effective preprocessing method compared to other ash reduction techniques.^27,564–566^ The relatively low capital and operating expenses, combined with higher conversion efficiencies outweigh the increased value of cleaner feedstock, making it an economically viable option for large-scale biomass processing.

By separating and removing high-ash fractions, air classification helps minimize waste and mitigate environmental impact. As shown in Figure 21, the particles that are entrained in the air stream at the end of a conveyor are often referred to as “light” particles, and those that are not entrained are referred to as “heavy” particles. Although not shown in Figure 21, the conveyor may also be perforated to allow fine particles to fall through the conveyor and eventually beneath the screen deck. Air classification enables the formulation of custom feedstock blends by producing distinct fractions with specific properties.^565^ These blends can be tailored to meet feedstock quality specifications required for different conversion processes, enhancing the flexibility and efficiency of biomass utilization. The separated high-ash fractions can also be repurposed for other applications, such as soil amendments, construction materials, or as a lower-grade fuel, adding value to what would otherwise be discarded as waste material. Thus, air classification provides a combination of economic, environmental, and operational benefits, making it a valuable tool for optimizing biomass utilization in sustainable energy and chemical production.

##### 7.2.1 Key Mechanisms of Mechanical Classification

The fundamental principle behind air classification is fluidization. When a stream of air flows upward through a bed of particles, it creates a fluid-like environment where the particles are suspended and can move freely. The behavior of each particle in this environment is governed by its aerodynamic properties, specifically its terminal velocity, defined as the constant speed that a particle reaches when the force of gravity pulling it downward is balanced by the drag force of the air pushing it upward. The terminal velocity is influenced by the particle size, shape, and density. Larger, denser particles have higher terminal velocities and tend to settle quickly, while smaller, lighter particles remain suspended and are carried away by the air stream. Cousins et al.^567^ report a case study for air separation of corn stover anatomical tissues. The counteracting forces during particle fluidization without particle entrainment are given in eq 9 where *C*_*D*_ is the coefficient of drag based on the particle shape, *ρ*_*f*_ is the fluidizing medium density, *u* is the velocity of the fluid, *ρ*_*p*_ is the particle density, *g* is the gravitational constant, and *V*_*p*_ is the particle volume. The separation or terminal velocity, *u*_0_, derived from eq 9, is defined with the particle mass, *M*_*p*_, when the fluidization forces exceed that of the gravitational forces by eq 10.

9 $$C_{D}\frac{\rho_{f}u^{2}}{2}A_{p}=\left(\rho_{p}-\rho_{f}\right)gV_{p}$$

10 $$u_{0}=\sqrt{\frac{2gM_{p}}{C_{D}\rho_{f}A_{p}}}$$

As a result, controlling the air velocity is crucial for effective air classification. By adjusting the speed of the air flow, operators can fine-tune the separation process to target specific fractions. Lower air velocities may only fluidize the lightest particles, while higher velocities can suspend and carry away denser particles. This ability to control air velocity allows for multiple passes through the classifier, progressively separating particles from finer to coarser fractions. For example, in a typical air classification setup, the process might start with a low air velocity to remove the lightest, most dust-like particles. The remaining material is then subjected to incrementally higher air velocities, each pass separating out progressively denser fractions until only the heaviest particles remain.

Complex relationships govern the size and shape distributions of particles by air classification. Irregularly shaped particles with larger surface areas experience greater drag forces, making them more likely to be carried away by the air stream even if they are relatively dense. Conversely, spherical particles with smaller surface areas experience less drag and tend to settle more quickly. For example, it has been shown that a pseudo particle density (based on cross-sectional area) follows a power law relationship where corn cobs are the most dense (2.3 mg/mm^2^) followed by nodal regions (1.8 mg/mm^2^), whole stalk (0.78 mg/mm^2^), stalk rind (0.37 mg/mm^2^), stalk pith (0.25 mg/mm^2^), sheath (0.20 mg/mm^2^), husk (0.13 mg/mm^2^), and finally leaves (0.04 mg/mm^2^).^567^ Based on these densities, experimental separation results based on air speeds and particle sizes/shapes yielded correlations of drag coefficients and cross-sectional area of these plant regions. These correlations led to fairly accurate (<10% error) prediction of particle entrainment for most tissues across several air classification stages in series.

Separation of particles based on their size and shape with screens or sieves is a widely deployed technique. In general, these devices operate with a flat or cylindrically rolled perforated sheet that is agitated to enable particle motion and separation. These devices typically operate with eccentric angles and motion but perform separation based on the size and shape of the perforations. As a result, the separation efficiency tends to depend on size differences, the style of the sieve screen, the magnitude and frequency of agitation, the mechanism by which the particles are in motion, the particle size and density, and the duration of the separation. Despite the wide adoption of these techniques, many approaches typically use empirically calibrated phenomenological models, such as the one presented in eq 11, where *W* is the weight of particles passing or retained on the sieve, depending on the defined convention, after *t* time, *W*_0_ is the initial sample mass, λ is a rate of separation with units of time^–1^.^568,569^ Similarly, the screening efficiency (ϵ) can be represented as shown in eq 12 and defined as the ratio of particles passed after a given time, *j* is the number of discretized size classes, λ is a size dependent rate, *w*_*i*_ is the mass ratio within the size range, *i*, and *u* is the mass ratio of particles that can be separated.

11 $$W\left(t\right)=W_{0}\exp\left(-\lambda t\right)$$

12 $$\epsilon \left(t\right)=1-\frac{1}{u}\overset{j}{\underset{i=1}{\sum }}w_{i}e_{i}^{-\lambda t}$$

Despite their simplicity, empirical models are the most broadly reported ones in the literature. Improved models have been developed incorporating population balance^570^ and other probabilistic frameworks.^571^ Discrete element modeling indicates that the most important factors influencing separation are agitation mode, magnitude, and frequency, as well as sieve inclination angle and length.^572,573^ Due to the low density, fibrous, and interlocking nature of biomass fibers, it is likely that the sieve path length influences the separation more than in dense, uniform aggregate materials.

##### 7.2.2 Application to Inorganics Removal from Biomass

Lacey et al.,^7,20,399^ performed a systematic study of the partitioning of inorganics based on size- and air-classification of forestry thinnings and logging residues. They found that air classification can effectively separate biomass into high- and low-ash content fractions. The lighter fractions, which contain the highest ash content, can be removed to significantly reduce the overall ash content in the remaining biomass. For both feedstocks, the inorganics were present at the highest concentration in the needles, followed by the bark, twigs, branches, cambium and the white wood. With increasing air flow rates from 10 to 25 std. ft^3^/min in the studied device, the forestry thinnings and logging residues separated into mass fractions and ash contents, as shown in Figure 22. The volumetric flow and resulting superficial velocity are controlled by the equipment blower, fit with a variable frequency drive (VFD) to linearly adjust air flow with the input AC frequency (Hz). For forestry thinnings, removing the below screen (BS) and 10 Hz fractions reduced the overall initial ash content from 1.7% to 1.1%, removing only 6.7% of the total biomass. Similarly, for logging residues, removing the BS and 10 Hz fractions reduced the overall initial ash content from 1.1% to 0.7%, also removing 6.7% of the biomass. Among the specific inorganics removed, it was found that Si, representing the exogenous inorganics or mostly soil contamination, was reduced in content by almost 70%. In contrast, Ca removal was only about 20%. Overall, the exogenous elements (e.g., Si, Al, Fe) are concentrated in the lighter fractions and are more easily removed through air classification. Physiological elements (e.g., Ca, K, Mg) are more uniformly distributed across the plant parts, making their selective removal more challenging. The study found that needles were predominantly found in the air classification fractions collected at 12, 15, and 18 Hz. While it is possible to selectively collect these fractions to isolate the needles, their removal has a minimal impact on the overall ash reduction because they constitute a small portion of the total biomass. Conversely, bark was distributed across all air-classified fractions, making its selective removal through air classification more challenging. The reported cost estimate for implementing air classification is $2.23 per dry ton, making it an economically attractive option for ash removal.^7,20,399^

**Figure 22:**
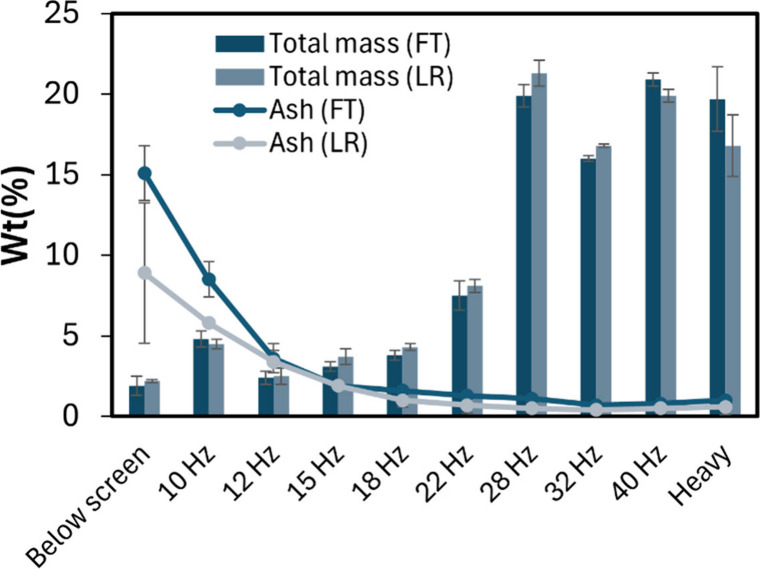
Influence of air velocity on extent of biomass fractionated and corresponding ash contents for loblolly pine forestry residues: forestry thinnings (FT) and logging residues (LR). Values shown are average weight percent ± 1 standard deviation. Adapted with permission from Lacey et al.^399^ Copyright 2015, Elsevier.

Thompson et al.^565^ investigated the impact of air classification on reducing high ash concentrations in corn stover, switchgrass, and grass clippings. They found that air classification effectively separates biomass into light and heavy fractions. Light fractions contained high concentrations of elemental ash components such as Na, Al, Si, Fe, and Ti from soil contamination, while heavy fractions were depleted in these components and had relatively lower total ash content. For each separation, the classifier fan was initially set to the lowest speed, and the shaker table was activated. A bag containing 3–5 kg of biomass was manually poured onto the infeed shaker table, ensuring it formed a single layer as it moved over the classifier air stream. The air flow separated the biomass into light and heavy fractions. The fan speed was then increased, and the heavy fraction was passed through the air classifier again to create new light and heavy fractions. This sequential/series process continued until light fractions were collected at all fan speeds and the remaining heavy fraction was gathered. Grass clippings exhibited the highest ash content, ranging from 14.4% in the 7.5 Hz heavy fraction to 11.4% in the remaining heavy fraction at 15 Hz, while switchgrass had the lowest ash content, with values of 6.6% in the 7.5 Hz heavy fraction and 2.5% in the 15 Hz heavy fraction. For corn stover, over 30–40% of the total ash was removed with only a 20% loss in biomass. Similarly, for switchgrass, this process resulted in 51% ash and 34% biomass removal, and for grass clippings, 32% ash and 24% biomass removal. Compared to a previous study^566^ on ash removal from pine thinnings and forest residues, where 41% of total ash was removed with only 6.7% biomass loss, the ash removal for herbaceous residues was less pronounced. Air classification effectively removes exogenous ash from soil contamination, with physiological ash removal proportional to biomass loss. For example, light fraction removal resulted in 39% Si removal with 20% biomass loss for corn stover; 56% Si removal with 34% biomass loss for switchgrass; and 32% Si removal with 24% biomass loss for grass clippings. The total cost of air classification was reported to be $1.05 per ton of classified biomass, which is lower than that for woody feedstocks, making it an even more attractive pathway to remove ash from herbaceous biomass.

Saha et al.^26^ investigated the impact of air classification on ash removal and pyrolysis yield using loblolly pine residues. Consistent with previous studies,^565,566^ they found that light fractions containing higher ash content resulted in lower bio-oil yields than heavier fractions. For example, the bio-oil yield difference between heavy and light fractions was as high as 15%. The study also demonstrated that effective separation requires higher air velocities when biomass has higher moisture content, which increases particle adhesiveness, ultimately affecting bio-oil quality.

In a related study, Emerson et al.^562^ used failure mode and effects analysis (FMEA) to investigate failures and effects in a pilot-scale woody biomass preprocessing facility. They evaluated failures related to the fixed carbon specification, analogous to those observed with moisture specification deviations. Critical quality attributes, including fixed carbon concentration and ash content, were affected primarily by inadequate bark removal during air classification. Increased moisture content, which affects separation efficiency, led to cascading failures and the highest risk priority score, which was linked to potential adverse effects in the high-temperature reactor. Insufficient bark removal increased inorganic components, particularly ash content, reducing the fixed carbon concentration and causing wear in downstream preprocessing units such as the hammer mill. The study identified moisture content and tissue fraction dimensions as primary factors contributing to deviations from the fixed carbon specification. When tissue fractions, such as smaller white wood fragments, closely resemble bark and needles in size, the separation by air classification leads to anomalous results.

Several articles^25,27^ have recently examined the impact of material attributes of loblolly pine on fast pyrolysis and potential cost implications of remediation with air classification. The conversion study^25^ reveals significant differences in the pyrolysis performance of various loblolly pine feedstocks, influenced by their anatomical fractions, tree age, and ash content. By reducing the ash content through classification at 10 and 28 Hz (from 1.6 wt % to 0.9 and 0.6 wt %, respectively), the residues exhibited a decrease in char production and an increase in light gas yields. With increasing extent of separation by air classification, the amount of extractives (proteins, waxes, resins, and other nonstructural soluble components) in the remaining fraction, measured using wet chemistry, also diminished, due to the removal of bark and needles. As a result, the relative proportions of carbohydrates on a summative basis were higher. Consequently, the cleaner, nonentrained portion of the air-classified residues produced higher oil yields and a more uniform product where the carbon content in the total recovered liquid increased from 33.2 wt % to 43.1 wt %, and the water decreased from 35.1 wt % to 26.5 wt %. Consistent with this trend, the isolated stemwood consistently produced the highest oil yields, reaching up to 72 wt %, in contrast with the bark which yielded the lowest oil. Mixtures of stemwood and needles or bark and needles demonstrated unique interactions, with stemwood and needles producing a two-phase oil indicative of increased dehydration reactions, while bark and needles yielded a single-phase oil, reducing char formation. Air classification of lignocellulosic biomass residues to produce a cleaner, nonentrained fraction effectively enhanced light gas production and overall pyrolysis efficiency. Economic analysis^27^ indicated that the additional costs and material losses are outweighed by the improved conversions and fuel yields. However, it was also noted that higher air classification severity leads to higher rates of organics removal in the entrained stream, thereby diminishing the benefit and economic potential. The economically optimal air classification speed was identified at 18 Hz, balancing conversion benefits against material losses.^27^ Furthermore, valorizing the material removed during preprocessing as a coproduct could further enhance the economic and sustainability aspects of the biorefinery. For example, valorizing the entrained stream of biomass removed during classification at $30/dry Mg reduced the minimum fuel sale price (MFSP) by up to $0.25, or around $0.20 at the optimal conditions without coproduct valuation.^27^

Investigations of particle sized-based separations of corn stover^7^ revealed that the inorganics were mostly concentrated in the leaves (10.4 wt %), followed by the sheath (6.9 wt %), nodal sections (4.0 wt %), husks (3.7 wt %), internode sections (3.5 wt %), and cobs (1.5 wt %), with the whole sample showing an ash content of 4.9 wt %. When a ground sample passed through a ∼19 mm particle retention screen (similar to how primary size reduction would be done prior to pelleting or preparation for biochemical conversion), fractions with smaller particle sizes showed higher concentrations of inorganic species. Particles with sizes greater than 9.5 mm have an ash content of 3.5%, while the smallest particles (<0.15 mm) had an ash content of 22.5%. The trade-offs between retention size and ash content are shown in Figure 23.

**Figure 23:**
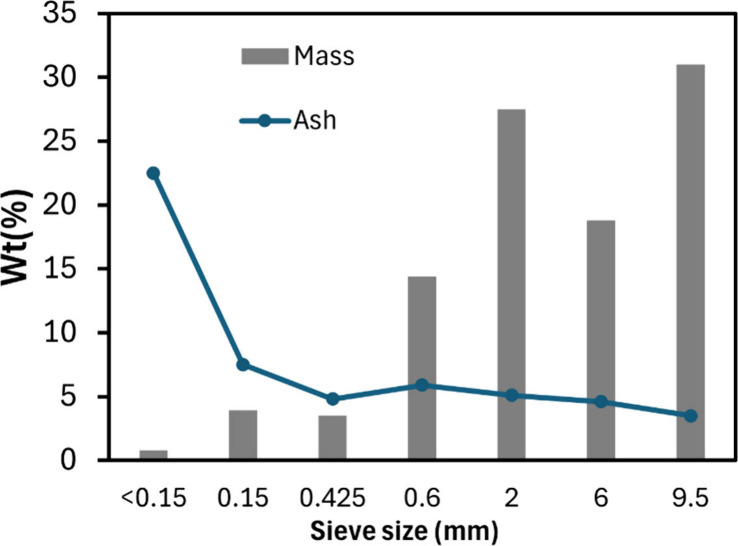
Distribution of mass by size retention in sieve analysis along with ash content. Adapted with permission from Lacey et al.^7^ Copyright 2016, Elsevier.

Ramirez-Quintero and Waldir^574^ compared air classification and mechanical separations by sieving several different biomass types, including bagasse, straw, bamboo, and cassava. In general, they found that the most significant reduction in inorganics was achieved around 1–2 m/s separation air velocity. On average, straw was reduced from 6.5% to 3.5%, cassava from 3.5% to 2%, bagasse from 3% to 1%, and bamboo from 2% to 1.5%. When examining the partitioning of inorganic concentrations at varying air speeds, they found similar trends for the different biomass types studied, with the most consistent result being the separation of exogenous inorganic species (soil contamination).

##### 7.2.3 Key Takeaways

Air classification and mechanical size separation are useful techniques for improving the quality of biomass feedstock by removing inorganics. These methods exploit variations in particle shape, size, and density to produce cleaner, low-ash biomass feedstocks, which significantly improve the conversion of the cleaned biomass, resulting in better-quality end products and fewer operational problems. Air classification is a cost-effective preprocessing method due to its relatively low capital and operational expenses, combined with the savings in conversion costs and the increased value of cleaner feedstock. Studies have shown that implementing air classification can reduce overall ash content significantly, thereby improving bio-oil yields and reducing char production during pyrolysis. For instance, reducing ash content in loblolly pine residues enhanced light gas production and overall pyrolysis efficiency.

However, the economic trade-offs must be considered. While the additional costs and material losses associated with air classification can be justified by the improved conversion efficiency and fuel yields, higher air classification severity could also lead to higher removal of clean biomass in the entrained stream, which would be considered as a loss of feedstock. Additionally, valorizing the removed high-ash fractions to make coproducts, such as soil amendments or construction materials, can further enhance economic viability and sustainability. Implementing air classification at an estimated cost of approximately $1.1–$2.2 per dry ton for woody and herbaceous biomass makes it an attractive option for large-scale biomass processing, improving both product quality and operational efficiency while minimizing environmental impact.

#### 7.3 Inorganics Removal by Water Washing and Chemical Leaching

To complement mechanical methods of mineral removal, leaching treatments using water or acid solutions have been shown to enhance product yield and improve the quality of conversion processes like fast pyrolysis.^575^ Leaching involves dissolving minerals in a liquid phase, thereby removing minerals from biomass and allowing selective recovery of desirable elements.^576^ Leaching can also extract inorganics embedded in the cell wall, which cannot be achieved by mechanical fractionation alone.^577^

##### 7.3.1 General Principles of Demineralization by Dissolution

Numerous process parameters can affect demineralization by leaching, including the type of leaching agent and process parameters such as time, temperature, and pH.^578^ These factors can be broadly understood by considering the various transport and reaction processes during mineral dissolution. Steps involved in dissolution include:

1) Diffusion (transport) of the bulk fluid and any species that promote dissolution to the minerals.
2) A reaction that breaks bonds to dissolve the mineral in the fluid.
3) Diffusion of the reaction products (e.g., dissolved ions) from the mineral to the bulk fluid.

For leaching minerals from biomass, steps (1) and (3) involve fluid transport through the porous biomass matrix. As discussed in Section 3, the minerals present in biomass include highly soluble species (e.g., water-soluble ions) that are loosely bound within the biomass matrix and are likely to be rapidly dissolved through the disruption of physical interactions such as hydrogen bonds in step (2). However, some inorganics can be tightly bound within the matrix or are present as solid mineral phases, which likely require longer dissolution times. We focus the remaining discussion on the dissolution of solid mineral phases, which is expected to be much slower than the removal of water-soluble species.^578^

For a solid mineral phase, a typical dissolution reaction in water has the form according to eq 13, where *M*_*x*_ is the mineral cation, *A*_*y*_ is the mineral anion, and *x* and *y* are stoichiometric coefficients. Dissolution is promoted at acidic pH, which increases the proton (H^+^) concentration and introduces anions that can interact with cationic mineral species. For example, generic reactions for the dissolution of calcium and potassium through acid treatment can be expressed as shown in eqs 14 and 15. *X* and *Y* in eqs 14 and 15 indicate unspecified anions from either the range of mineral compounds that may be present in biomass or from the added acid. Dissolution reactions occur via surface-mediated bond breaking with rates that depend upon the available surface area.^579^ pH is also known to strongly influence the rate of dissolution (*r*_diss_).^580,581^

13 $$M_{x}{A_{y}}_{\left(s\right)}+y{H^{+}}_{\left(aq\right)}⇌x{M^{n+}}_{\left(aq\right)}+yHA_{\left(aq\right)}$$

14 $$CaX_{\left(s\right)}+2HCl_{\left(aq\right)}\to CaC{l_{2}}_{\left(aq\right)}+H_{2}X_{\left(aq\right)}$$

15 $$KY_{\left(s\right)}+HN{O_{3}}_{\left(aq\right)}\to KN{O_{3}}_{\left(aq\right)}+HY_{\left(aq\right)}$$

16 $$r_{\mathrm{diss}}=A_{S}ka_{H^{+}}^{n}\left(1-\frac{Q}{K_{eq}}\right)$$

These factors can be combined into a general rate law for mineral dissolution as shown in eq 16. In this expression, *A*_*S*_ is the surface area of the mineral available to react, *k* is the intrinsic rate constant for the reaction, *a*_*H* ^+^_^*n*^ quantifies the dependence of the rate on the pH as expressed through the activity of protons (*a*_*H* ^+^_) and a reaction-specific exponent *n*, *Q* is the ion activity product, and *K*_*eq*_ is the equilibrium constant for the reaction.^578^ The ion activity product is the product of dissolved ion activities raised to their stoichiometric coefficients as shown in eq 17 where *m*_*i*_ is the molality of species *i*, *v*_*i*_ is the stoichiometric coefficient (positive for products, negative for reactants), and *γ*_*i*_ is the activity coefficient to account for thermodynamic nonidealities in solutions with complex compositions. *Q* evolves as a reaction proceeds and the concentrations of dissolved species change.^578^

17 $$Q=\prod {\left(\gamma_{i}m_{i}\right)}^{\nu_{i}}$$

18 $$K_{eq}=\prod {\left(\gamma_{i}m_{i}\right)}^{\nu_{i}}=\exp\left(-\frac{\Delta G^{{}^{\circ}}}{RT}\right)$$

The equilibrium constant shown in eq 18 will determine the maximum solubility of a given mineral at a particular pH and temperature, in addition to affecting the rate of dissolution because the ratio *Q*/*K*_*eq*_ determines how close a reaction is to equilibrium (*Q*/*K*_*eq*_ = 1). The equilibrium constant depends upon the temperature and the species present in the reaction (e.g., specific anions and cations) which will influence Δ*G*°.^578,582^ These factors also influence the rate constant, which can be related to temperature through an Arrhenius equation shown in eq 19, with pre-exponential factor *A* and activation energy *E*_*A*_.

19 $$k=A\exp\left(-\frac{E_{A}}{RT}\right)$$

Together, eqs 16–19 highlight the factors that affect the thermodynamics and kinetics of mineral dissolution.^578,583^ However, demineralization also requires fluid transport through the biomass matrix during steps (1) and (3) of the framework introduced above; these processes could also limit the rate of mineral removal. The concentration of fluid within a biomass particle *C* during a leaching process can be described by Fick’s second law shown in eq 20, which is written in one dimension for simplicity. *D* is the diffusion coefficient, which itself will depend upon the temperature and porosity of the biomass matrix, and *x* is the spatial distance.^578,584^

20 $$\frac{\partial C}{\partial t}=D\frac{\partial^{2}C}{\partial^{2}x}$$

While this simplified expression omits the complexities of geometry associated with real materials, it highlights that diffusion into and out of the biomass matrix will also impact the rate of biomass demineralization, particularly if demineralization is transport-limited, through factors such as the diffusion coefficient and the size and geometry of the biomass sample.^578,583^

##### 7.3.1.1 Factors Influencing Mineral Removal and Their Trends

The preceding general conceptual framework for mineral dissolution, while not unique to biomass, can be used to understand the large number of factors that have been experimentally reported to impact leaching-based demineralization processes. Table 15 qualitatively summarizes these factors, including the type of biomass feedstock, type of leaching agent, leaching time, particle size/porosity, temperature, liquid-to-solid ratio, pH, and concentration/stoichiometric ratio of leaching agent.^576^ We can interpret these factors as impacting the concentrations of different minerals in the feedstock, the equilibrium constants and rates for different dissolution processes, the time required for mass transport into/out of the biomass matrix, and the thermodynamics of dissolution reactions. Quantitative analysis of specific mineral removal efficiencies for different leaching methods is provided in the following sections.

**Table 15:** Qualitative Descriptions of Major Factors Affecting Mineral Removal. Sections 7.3.2–7.3.6 Provide a Quantitative Analysis of These Factors.

Factor	Description	Magnitude/Impact
pH	The pH of the leaching solution is determined by acid/base strength and concentration. Adjusting the pH enables selective leaching by targeting specific inorganic groups that dissolve more efficiently under certain pH conditions.^585^	*Neutral pH*: Effective for soluble salts (K^+^, Na^+^, Cl^–^). Preserves biomass integrity. Ineffective for embedded minerals.^586,587^
		*Acidic pH*: Effective for K^+^, Mg^2+^, Ca^2+^, chlorides, and sulfates. High mineral removal but biomass degradation, organic matter loss, and environmental concerns.^586,587^
		*Alkaline pH*: Effective for silicates, phosphates, and lignin components. Modifies the structure and increases cellulose accessibility but leads to Na^+^ retention and limited divalent cation removal.^586,587^
Leaching agent	The leaching agent refers to the specific species added to the leaching solution. Examples include different types of acids (mineral or organic) and chelating agents.	Acids (mineral or organic) can remove minerals embedded in the lignocellulosic matrix, with organic acids (e.g., acetic acid) achieving high mineral removal efficiency with less biomass degradation than mineral acids.^576^ Chelating agents (e.g., sodium citrate) can selectively bind structural minerals without damaging cellulose and hemicellulose.^588^
Particle size	The physical size of the biomass particle is determined by the type of biomass and processing steps.	Smaller particle sizes increase the surface area in contact with the leaching solution, facilitating mineral dissolution and enhancing leaching efficiency.^589^
Leaching time	Leaching time is the duration that the biomass is exposed to the leaching agent.	Longer leaching times increase mineral removal efficiency, but the removal rate slows down over time, and long times heighten the risk of biomass degradation.^589,590^
Temperature	The temperature refers to the temperature at which the leaching process is performed.	Higher temperatures improve mineral dissolution but may accelerate biomass degradation.^591^ Higher water temperatures can also affect the moisture remaining in the biomass after washing, which could impact downstream processing.^591^

##### 7.3.1.2 Quantification of Demineralization Processes

The prior section highlights the qualitative factors expected to influence demineralization through leaching. However, the same factors that promote mineral dissolution may also degrade the biomass itself, which is undesirable for downstream processing. Assessing the trade-off between demineralization and biomass degradation requires metrics to quantify these processes to enable the development of efficient demineralization strategies that minimize biomass degradation and enhance product yields from resulting thermochemical treatments. Unfortunately, such metrics have not yet been widely adopted or consistently reported in the literature on biomass demineralization. We therefore highlight possible methods for quantification while establishing metrics that will be discussed in Sections 7.3.2–7.3.7.

Effective removal efficiency considering mass loss can be quantified by the reduction of mineral substances during leaching according to eq 21 ^143,592^ where *i* is the element or constituent that was removed, *m*_*u*_ is the mass of the raw biomass sample, *m*_*w*_ is the mass of the biomass sample washed by water or a leaching agent, and *C*_*i*__,*u*_ and *C*_*i*__,*w*_ are the mass fractions of the element or constituent in the raw biomass sample and the washed biomass sample, respectively.

21 $$\mathrm{Effective Removal Efficiency}\;\left(\%\right)=\left(1-\frac{m_{w}\times C_{i,w}}{m_{u}\times C_{i,u}}\right)\times 100$$

While mineral removal efficiencies are not consistently reported in the literature, we adopt this metric to reflect the mass loss and to compare leaching treatments in the remainder of this section, using available literature data to compute the removal efficiency when possible. For studies that did not explicitly account for mass loss, the reported removal efficiencies in Tables 16–19 were either taken directly from the literature or calculated using eq 21 without incorporating mass loss (i.e., calculated based only on the initial raw biomass weight), and are typically identified only as “Removal efficiency”.

**Table 16:** Effective Removal Efficiencies of Water Washing on Biomass Feedstocks. Tables 3–5 Show the Ash and Inorganic Content of Raw Biomass.

	Leaching condition					Effective Removal Efficiency (%)									
Biomass	T (°C)	Time (h)	N	S	P	K	Ca	Mg	Na	Al	Fe	Si	Ash	Biomass loss (wt %)	Ref.
Barley straw	50	4	n.a.	n.a.	100.0	83.6	28.7	55.4	85.6	44.7	36.9	n.a.	42.2	12.8	^607^
Candlenut wood^a^	30	3	n.a.	75.3^c^	n.a.	70.1^c^	n.a.	n.a.	n.a.	n.a.	n.a.	n.a.	7.7^c^	n.a.	^591^
	60	3	n.a.	75.8^c^	n.a.	85.0^c^	n.a.	n.a.	n.a.	n.a.	n.a.	n.a.	30.5^c^	n.a.	^591^
	90	3	n.a.	76.2^c^	n.a.	87.5^c^	n.a.	n.a.	n.a.	n.a.	n.a.	n.a.	32.1^c^	n.a.	^591^
Corn stalk^a^	30	3	n.a.	91.9^c^	n.a.	83.9^c^	n.a.	n.a.	n.a.	n.a.	n.a.	n.a.	41.8^c^	n.a.	^591^
	60	3	n.a.	88.2^c^	n.a.	84.3^c^	n.a.	n.a.	n.a.	n.a.	n.a.	n.a.	52.3^c^	n.a.	^591^
	90	3	n.a.	96.4^c^	n.a.	85.6^c^	n.a.	n.a.	n.a.	n.a.	n.a.	n.a.	63.0^c^	n.a.	^591^
Cotton stalk^a^	30	3	n.a.	90.2^c^	n.a.	84.6^c^	n.a.	n.a.	n.a.	n.a.	n.a.	n.a.	63.3^c^	n.a.	^591^
	60	3	n.a.	90.6^c^	n.a.	85.0^c^	n.a.	n.a.	n.a.	n.a.	n.a.	n.a.	63.8^c^	n.a.	^591^
	90	3	n.a.	91.1^c^	n.a.	89.8^c^	n.a.	n.a.	n.a.	n.a.	n.a.	n.a.	68.5^c^	n.a.	^591^
Douglas fir	121	0.5	n.a.	n.a.	n.a.	85.0	66.3	77.5	97.3	n.a.	n.a.	n.a.	70.0	10	^616^
Eucalyptus	50	4	n.a.	n.a.	100.0	90.2	17.1	53.7	88.9	5.0	56.4	n.a.	29.8	10.6	^607^
Hybrid poplar	121	0.5	n.a.	n.a.	n.a.	82.6	91.0	33.6	73.4	n.a.	n.a.	n.a.	28.3	7	^616^
Miscanthus	50	4	n.a.	n.a.	100.0	80.2	22.1	42.0	72.6	57.6	47.4	n.a.	23.0	4.4	^607^
Oak	50	4	n.a.	n.a.	100.0	100.0	14.2	39.8	100.0	4.0	6.5	n.a.	59.3	10.6	^607^
Pine	50	4	n.a.	n.a.	100.0	89.5	15.6	35.4	100.0	39.9	19.3	n.a.	43.1	7.0	^607^
Rice hull^a^	30	3	n.a.	80.6^c^	n.a.	81.9^c^	n.a.	n.a.	n.a.	n.a.	n.a.	n.a.	3.8^c^	n.a.	^591^
	60	3	n.a.	61.1^c^	n.a.	88.7^c^	n.a.	n.a.	n.a.	n.a.	n.a.	n.a.	4.99^c^	n.a.	^591^
	90	3	n.a.	81.2^c^	n.a.	90.5^c^	n.a.	n.a.	n.a.	n.a.	n.a.	n.a.	11.3^c^	n.a.	^591^
Rice straw^a^	30	3	n.a.	91.0^c^	n.a.	89.1^c^	n.a.	n.a.	n.a.	n.a.	n.a.	n.a.	19.9^c^	n.a.	^591^
	60	3	n.a.	95.6^c^	n.a.	89.1^c^	n.a.	n.a.	n.a.	n.a.	n.a.	n.a.	29.5^c^	n.a.	^591^
	90	3	n.a.	91.5^c^	n.a.	90.1^c^	n.a.	n.a.	n.a.	n.a.	n.a.	n.a.	41.9^c^	n.a.	^591^
Sugar cane bagasse	25	1	10.0	n.d.	n.a.	85.4	n.a.	37.9	55.7	41.9	42.5	52.9	38.4	11.2^c^	^617^
Sugar cane trash	25	1	25.6	n.d.	n.a.	94.3	n.a.	37.6	89.9	56.5	53.7	(8.3)	26.3	7.9^c^	^617^
Switchgrass	62	0.08	(50.0)	35.6	71.7	98.0	39.1	66.6	n.a.	n.a.	37.4	17.1	49.9	5.9	^599^
	78	0.17	0.0	29.7	83.9	98.9	38.7	66.9	n.a.	n.a.	32.9	8.4	40.1	4.7	^599^
	95	0.25	0.0	39.6	70.8	99.4	42.9	69.5	n.a.	n.a.	39.3	22.5	50.1	5.7	^599^
	109	0.33	0.0	41.2	88.3	99.4	45.9	71.8	n.a.	n.a.	37.7	28.0	43.9	4.9	^599^
Wheat straw^a^	30	3	n.a.	91.5^c^	n.a.	90.5^c^	n.a.	n.a.	n.a.	n.a.	n.a.	n.a.	57.3^c^	n.a.	^591^
	60	3	n.a.	88.7^c^	n.a.	91.0^c^	n.a.	n.a.	n.a.	n.a.	n.a.	n.a.	65.0^c^	n.a.	^591^
	90	3	n.a.	86.2^c^	n.a.	91.2^c^	n.a.	n.a.	n.a.	n.a.	n.a.	n.a.	75.7^c^	n.a.	^591^
	50	4	n.a.	n.a.	100.0	84.9	42.3	63.0	91.2	23.4	39.4	n.a.	17.2	12.8	^591^
Cardoon^d^	80	2	n.a.	42.8	87.8	98.3	n.a.	n.a.	n.a.	n.a.	n.a.	n.a.	27.0	n.a.	
Giant Reed^d^	80	2	n.a.	77.9	92.4	97.5	n.a.	n.a.	n.a.	n.a.	n.a.	n.a.	65.9	n.a.	
Loblolly pine bark^d^	140	0.75	n.a.	n.a.	n.a.	67.5	33.0	50.8	60.1	n.a.	n.a.	n.a.	73.3	n.a.	^618^
Poplar wood chips^d^	25	96	n.a.	(11.5)	76.5	87.5	8.0	27.2	0.0	n.a.	(48.7)	4.8	25.0	4.2^b^	^619^
Rice husk^d^	25	2	16.4	25.0	n.a.	92.0	10.2	40.2	84.7	n.a.	n.a.	n.a.	13.7	n.a.	^614^
Rice straw^d^	25	2	25.5	6.3	n.a.	82.3	17.1	33.5	80.4	n.a.	56.6	n.a.	33.3	n.a.	^605^
	50	2	0.0	n.a.	n.a.	82.6^c^	11.9^c^	22.6^c^	72.2^c^	n.a.	n.a.	n.a.	3.8	n.a.	^620^
Rice Straw (Andalusia)^d^	n.a.	n.a.	n.a.	25.5	0.0	49.1	n.a.	18.0	60.0	n.a.	n.a.	n.a.	19.8	n.a.	^606^
Rice Straw (Egypt)^d^	n.a.	n.a.	n.a.	59.4	7.8	26.1	n.a.	22.0	35.7	n.a.	n.a.	n.a.	17.1	n.a.	^606^
Rice Straw (Murcia)^d^	n.a.	n.a.	n.a.	30.3	0.0	49.5	n.a.	0.0	40.1	n.a.	n.a.	n.a.	10.7	n.a.	^606^
Rice Straw (Valencia)^d^	n.a.	n.a.	n.a.	55.2	34.8	49.4	n.a.	38.8	52.9	n.a.	n.a.	n.a.	15.8	n.a.	^606^
Switchgrass^d^	140	0.75	n.a.	n.a.	n.a.	98.8	13.0	83.5	72.4	n.a.	n.a.	n.a.	69.6	n.a.	^618^
	80	2	n.a.	77.7	92.2	98.9	n.a.	n.a.	n.a.	n.a.	n.a.	n.a.	62.5	n.a.	

a Concentrations were originally reported as metal oxides and were converted to mineral concentrations using stoichiometric calculations.

b All masses of reported chemical composition data were added up when calculating biomass loss.

c The data was not provided as a table in the literature, it was estimated from the graph.

d Removal efficiencies were taken directly from literature or calculated according to eq 21, based only on the initial raw biomass weight (*m*_*w*_ *=m*_*u*_). n.a. = not applicable (not reported). n.d. = not detectable. Negative removal efficiencies are represented in parentheses and were calculated based on the mineral concentrations reported in the literature.

The quantification of biomass degradation and associated carbon loss is more challenging. One metric developed to compare pulping processes in the paper industry is the H-factor, which combines pulping time and temperature into a single value, representing the extent of pulping (delignification).^593,594^ A similar concept, the severity factor (*R*_0_), was adapted to compare pretreatment methods for lignocellulosic biomass.^595,596^ *R*_0_ is shown in eq 22, where *t* is the leaching time of treatment in min, *T*(*t*) is the treatment temperature, and 100 is the reference temperature. The arbitrary constant (i.e., 14.75) is based on activation energy under pseudo-first-order kinetics^597^ and is commonly used without further optimization.^598^ *R*_0_ has been calculated to account for reduced ash and inorganic compounds after demineralization and considers the leaching time, temperature, and pH of the extraction solution. As shown in eq 23, a correction to the severity factor, *R*_0_^″^, was further developed to consider both acidic and alkaline pretreatments equally by incorporating pH deviations from neutrality,^596,599^ providing a more balanced evaluation of demineralization that could, in principle, be combined with the definition of the effective mineral removal efficiency (eq 21) to assess trade-offs between different demineralization processes. In this context, a higher value of *R*_0_^″^ indicates a more aggressive and severe pretreatment. However, while this metric does permit quantitative comparisons, it does not directly measure biomass degradation.

22 $$R_{0}=\int_{a}^{b}\exp\left(\frac{T\left(t\right)-100}{14.75}\right)dt=t\cdot \exp\left(\frac{T\left(t\right)-100}{14.75}\right)s$$

23 $$\log\left(R_{0}^{’’}\right)=\log\left(R_{0}\right)+\left|pH-7\right|$$

To assess degradation in the sections below, we also list carbon loss where reported, but we note that most studies do not distinguish between carbon or biomass loss due to the removal of extractives^600,601^ (e.g., waxes, starches) and loss due to the true degradation of polymeric biomass components (e.g., cellulose, hemicellulose, lignin). Thus, the reported values of total carbon or biomass loss may include loss of water-soluble extractives and not necessarily structural degradation.

The concentrations of inorganic elements vary depending on the biomass type, how the biomass was harvested and how the biomass was grown, as presented in Tables 3–5 and summarized in Figure 7. A typical removal efficiency of 97.8% or higher is required for woody biomass, 98.8% for herbaceous biomass, and 99.4% for agricultural residues to obtain less than 100 ppm of Ca, K, and N in the washed biomass. Similarly, to reduce the concentration of the major inorganic elements (Ca, K) and N to less than 10 ppm in the washed biomass, the removal efficiency should be higher than 99.8%, 99.9%, and 99.94% for woody biomass, herbaceous biomass, and agricultural residues, respectively. Other elements may require lower removal efficiencies, depending on their initial concentrations in the biomass. Figure 24 shows the effective removal efficiency, calculated according to eq 21, that is needed to reduce the concentration of inorganics to 10, 50, 100, or 250 ppm as a function of the initial concentration of inorganics in the raw biomass, assuming 5% and 25% biomass mass loss due to extractives.

**Figure 24:**
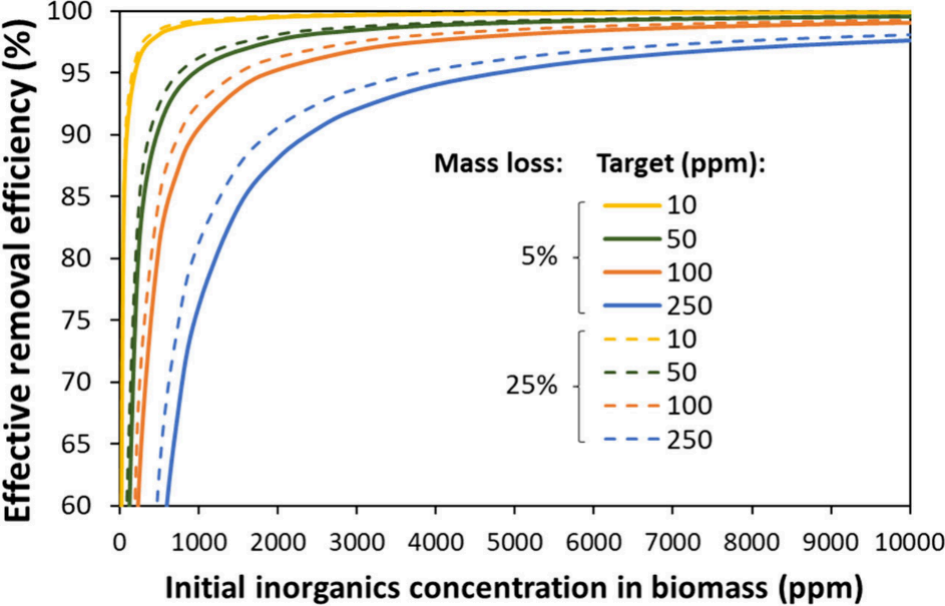
Effective removal efficiency of inorganics from biomass needed to reduce the inorganics concentration below 10, 50, 100, or 250 ppm, considering 5% or 25% mass loss. Values are calculated according to eq 21 as a function of the initial inorganics concentration.

##### 7.3.2 Water Washing

Water washing is a simple treatment for removing minerals in which no additional leaching agent is added to promote mineral dissolution. Table 16 lists quantitative mineral removal efficiencies for water washing applied at different conditions and to different feedstocks. In this table, removal efficiencies were calculated using eq 21 by comparing the reported mass of inorganic elements in biomass samples before and after demineralization in each listed reference. Negative removal efficiencies indicate a reported increase in inorganic content after treatment, which can be due to the presence of trace minerals (e.g., Ca, Na, Al, and Si) in the leaching solvents or glassware used during the demineralization process.^602^ Biomass loss was either taken directly from studies that explicitly reported mass loss or, when such values were not reported, estimated by applying eq 21 to the difference in the sum of chemical composition components (e.g., sugars) before and after demineralization. All removal efficiencies reported in the table are expressed as a percentage of the initial mineral content, which itself will vary with feedstock reported in Tables 3–5. In some cases, a range of removal efficiencies is reported in the table rather than a single value. This is because multiple data under different treatment conditions in the same literature yielded comparable results. These similar outcomes were represented as a range to avoid unnecessary repetition. This same overall approach was used to report values in Tables 17, 18, and 19 associated with text in Sections 7.3.3–7.3.5 below.

**Table 17:** Effective Removal Efficiencies of Mineral Acid Leaching on Biomass Feedstocks. Tables 3–5 Show the Ash and Inorganic Content of Raw Biomass.

	Leaching conditions							Effective removal efficiency (%)									
Biomass	T (°C)	Time (h)	Acid type	Acid conc.	N	S	P	K	Ca	Mg	Na	Al	Fe	Si	Ash	Biomass loss (wt %)	Ref.
Barley straw	50	2	HNO_3_	1 wt %	n.a.	n.a.	100.0	99.2	95.1	92.8	83.3	22.3	68.6	n.a.	54.0	14.5	^607^
Eucalyptus	50	2	HNO_3_	1 wt %	n.a.	n.a.	100.0	100.0	78.3	100.0	100.0	24.3	37.4	n.a.	69.9	10.9	^607^
Miscanthus	50	2	HNO_3_	1 wt %	n.a.	n.a.	100.0	98.7	96.9	90.5	87.8	65.7	65.8	n.a.	38.6	4.9	^607^
Oak	50	2	HNO_3_	1 wt %	n.a.	n.a.	100.0	100.0	100.0	100.0	100.0	33.6	67.4	n.a.	89.8	10.4	^607^
Pine	50	2	HNO_3_	1 wt %	n.a.	n.a.	100.0	100.0	100.0	100.0	100.0	62.5	64.0	n.a.	87.8	7.8	^607^
Poplar wood powder	60	6	HCl	2 M	30.7	n.a.	52.4	99.9	97.8	98.1	58.2	(11.7)	75.7	(312)	89.1	30.7	^602^
	25	1	HF	3 wt %	2.7	n.a.	12.8	99.4	25.0	78.2	(20.5)	(146)	17.3	(241)	83.3	2.7	^602^
Sugar cane bagasse	25	1	HCl	0.18 kg/L	60.0	n.d.	n.a.	99.2	n.a.	93.0	53.6	68.5	73.1	66.5	53.7	22.4^c^	^617^
	25	1	H_2_SO_4_	0.50 kg/L	60.0	n.d.	n.a.	99.3	n.a.	92.6	100.0	65.0	75.3	59.1	53.7	19.5^c^	^617^
Sugar cane trash	25	1	HCl	0.18 kg/L	47.2	n.d.	n.a.	99.4	n.a.	97.5	23.8	78.0	77.9	4.1	40.2	12.0^c^	^617^
	25	1	H_2_SO_4_	0.50 kg/L	47.2	n.d.	n.a.	99.5	n.a.	96.4	62.4	75.6	73.7	27.8	40.2	11.9^c^	^617^
Switchgrass	69	0.08	H_2_SO_4_	10 g/L	(50.0)	33.1	74.9	98.8	99.2	99.4	n.a.	n.a.	71.7	23.7	70.8	7.3	^599^
	85	0.17	H_2_SO_4_	10 g/L	0.0	29.7	90.1	99.3	99.2	99.5	n.a.	n.a.	73.9	13.0	58.5	10.0	^599^
	105	0.25	H_2_SO_4_	10 g/L	(50.0)	44.6	81.5	98.8	99.5	99.6	n.a.	n.a.	85.4	18.8	54.7	22.0	^599^
	121	0.33	H_2_SO_4_	10 g/L	(50.0)	45.0	94.3	99.2	99.6	99.6	n.a.	n.a.	89.6	22.4	59.7	32.9	^599^
Wheat straw	50	2	HNO_3_	1 wt %	n.a.	n.a.	100.0	99.5	97.8	97.3	96.6	58.2	19.1	n.a.	21.8	14.3	^607^
Moso bamboo^d^	25	2	HCl	3 wt %	n.a.	38.1	63.2	98.2	69.4	66.8	10.5	n.a.	72.0	n.a.	n.a.	n.a.	^627^
	25	2	HNO_3_	3 wt %	n.a.	38.4	63.0	94.8	51.5	52.1	17.7	n.a.	66.2	n.a.	n.a.	n.a.	^627^
	25	2	HF	3 wt %	n.a.	31.4	71.6	95.6	59.3	38.3	(3.3)	n.a.	35.3	n.a.	n.a.	n.a.	^627^
	25	2	H_2_SO_4_	3 wt %	n.a.	(62.2)	66.0	96.6	48.8	44.7	33.3	n.a.	70.1	n.a.	n.a.	n.a.	^627^
Palm empty fruit bunches^a^,^d^	20	48	HCl	2 M	66.5	59.1	64.1	96.3	93.9	94.8	(39.1)	(196)	16.4	(245)	62.9	n.a.	^623^
	25	48	HClO_4_	2 M	52.9	53.5	66.0	97.4	97.5	96.5	39.1	(156)	(19.7)	(230)	55.7	n.a.	^623^
	25	48	H_2_SO_4_	2 M	60.0	56.5	60.4	97.7	96.0	97.0	n.a.	(160)	36.0	(244)	58.6	n.a.	^623^
	25	48	HF	2 M	93.1	94.5	(10.3)	98.5	(172)	(38.3)	n.a.	(40.3)	(45.5)	82.1	93.9	n.a.	^623^
	25	48	HNO_3_	2 M	31.3	66.0	73.4	97.4	92.7	94.6	13.0	(257)	(74.4)	(258)	60.0	n.a.	^623^
Palm kernel shell^a^,^d^	20	48	HCl	2 M	71.4	71.8	23.5	(7.8)	98.2	78.0	(740)	(360)	(433)	(204)	71.8	n.a.	^623^
	25	48	HClO_4_	2 M	69.4	74.4	4.1	(10.9)	98.8	85.9	(46.9)	(270)	(512)	(191)	70.6	n.a.	^623^
	25	48	H_2_SO_4_	2 M	65.6	69.5	7.1	1.6	91.4	89.3	(15.6)	(330)	(510)	(145)	66.9	n.a.	^623^
	25	48	HF	2 M	22.4	40.7	76.5	86.0	3.5	55.4	50.0	20.5	23.7	95.1	33.7	n.a.	^623^
	25	48	HNO_3_	2 M	58.5	76.8	31.6	12.4	92.0	84.2	(78.1)	(237)	(540)	(183)	70.6	n.a.	^623^
Palm mesocarp fiber^a^,^d^	20	48	HCl	2 M	29.8	30.3	74.0	90.9	99.1	95.5	28.0	(94.1)	24.3	(151)	27.5	n.a.	^623^
	25	48	HClO_4_	2 M	28.4	36.3	78.3	90.7	97.4	96.4	40.0	(80.8)	5.3	(139)	27.4	n.a.	^623^
	25	48	H_2_SO_4_	2 M	32.0	34.2	80.2	90.9	98.1	96.4	48.0	(86.3)	23.3	(156)	25.0	n.a.	^623^
	25	48	HF	2 M	82.7	87.2	56.4	77.5	(29.7)	33.1	(66.7)	(80.1)	9.6	79.4	83.9	n.a.	^623^
	25	48	HNO_3_	2 M	(9.4)	41.6	81.2	92.8	96.3	96.4	32.0	(74.5)	9.1	(168)	22.6	n.a.	^623^
Pine bark^d^	20	48	HCl	0.5 M	n.a.	25.0	n.a.	92.2	89.6	97.8	100.0	66.0	66.7	n.a.	n.a.	n.a.	^628^
Poplar wood chips^d^	80	3	H_2_SO_4_	0.05 M	n.a.	(1629)	43.6	68.4	48.7	70.8	9.9	n.a.	4.2	(6.7)	70.0	9.3	^619^
Rice husk^a^,^d^	25	2	HCl	2 M	n.a.	98.4	72.1	99.1	93.3	95.6	n.a.	46.8	(133)	(4.7)	4.3	n.a.	^613^
	25	2	HF	2 M	n.a.	88.1	70.3	96.7	72.5	88.7	92.5	60.1	71.2	99.1	98.1	n.a.	^613^
	25	2	H_3_PO_4_	2 M	n.a.	96.5	43.1	99.0	81.6	90.0	n.a.	44.5	(126)	(3.7)	4.8	n.a.	^613^
	25	2	HCl	5 wt %	17.9	37.5	n.a.	99.7	86.3	98.8	90.4	n.a.	n.a.	n.a.	17.9	n.a.	^614^
Rice straw^d^	25	2	HF	3 wt %	11.1	n.a.	76.6	96.3	33.8	86.9	89.6	3.0	73.7	(36.1)	93.1	(20)	^629^
	25	2	H_2_SO_4_	5 wt %	11.8	31.3	n.a.	99.8	95.7	98.6	88.5	n.a.	68.5	n.a.	46.0	n.a.	^605^
	25	2	HCl	5 wt %	22.5	37.5	n.a.	99.7	97.9	99.2	88.1	n.a.	76.3	n.a.	50.7	n.a.	^605^
	25	2	HNO_3_	5 wt %	14.7	31.3	n.a.	99.7	96.8	99.1	84.8	n.a.	69.5	n.a.	54.7	n.a.	^605^
	25	2	H_3_PO_4_	5 wt %	18.6	25.0	n.a.	99.7	62.3	98.7	86.2	n.a.	57.9	n.a.	47.7	n.a.	^605^
	50	2	HCl	pH 2.9	14.3	n.a.	n.a.	99.33^b^	93.6^b^	97.2^b^	90.6^b^	n.a.	n.a.	n.a.	30. 8	n.a.	^620^
Wheat straw^d^	25	2	HCl	1.4 M	66.2	85.0	n.a.	77.4	89.1	88.0	65.1	n.a.	n.a.	n.a.	n.a.	n.a.	^630^
	25	2	HF	1.4 M	64.4	85.0	n.a.	70.6	85.6	87.6	57.9	n.a.	n.a.	n.a.	n.a.	n.a.	^630^
	25	2	HCl+HF	1.4 M	40.6	77.5	n.a.	68.8	46.0	45.3	23.7	n.a.	n.a.	n.a.	n.a.	n.a.	^630^

a Concentrations were originally reported as metal oxides and were converted to mineral concentrations using stoichiometric calculations.

b All masses of reported chemical composition data were added up when calculating biomass loss.

c The data was not provided as a table in the literature, it was estimated from the graph.

d Removal efficiencies were taken directly from literature or calculated according to eq 21, based only on the initial raw biomass weight (*m*_*w*_ *=m*_*u*_). n.a. = not applicable (not reported). n.d. = not detectable. Negative removal efficiencies are represented in parentheses and were calculated based on the mineral concentrations reported in the literature.

**Table 18:** Effective Removal Efficiencies of Organic Acid Leaching on Biomass Feedstocks. Tables 3–5 Show the Ash and Inorganic Content of Raw Biomass.

	Leaching conditions						Effective removal efficiency (%)										
Biomass	T (°C)	Time (h)	Acid type	Acid conc.	N	S	P	K	Ca	Mg	Na	Al	Fe	Si	Ash	Biomass loss (wt %)	Ref.
Barley straw	50	2	Acetic acid	1 wt %	n.a.	n.a.	100.0	99.7	59.5	87.1	99.2	78.1	67.4	n.a.	49.4	14.1	^607^
Eucalyptus	50	2	Acetic acid	1 wt %	n.a.	n.a.	100.0	96.1	22.7	88.8	94.8	(18.2)	43.0	n.a.	19.8	10.8	^607^
Miscanthus	50	2	Acetic acid	1 wt %	n.a.	n.a.	100.0	99.0	56.4	84.8	89.6	73.9	53.4	n.a.	31.5	4.7	^607^
Oak	50	2	Acetic acid	1 wt %	n.a.	n.a.	100.0	100.0	41.7	97.7	100.0	26.5	7.1	n.a.	76.7	9.8	^607^
Pine	50	2	Acetic acid	1 wt %	n.a.	n.a.	100.0	100.0	73.1	100.0	100.0	57.0	23.4	n.a.	77.3	8.0	^607^
Sugar cane bagasse	25	1	Citric acid	0.192 kg/L	55.0	n.d.	n.a.	99.2	n.a.	78.6	86.0	57.7	66.1	54.0	47.9	13.0^c^	^617^
Sugar cane trash	25	1	Citric acid	0.192 kg/L	45.4	n.d.	n.a.	99.3	n.a.	94.5	92.0	67.1	61.1	57.2	36.5	8.6^c^	^617^
Switchgrass	61	0.08	Acetic acid	10 g/L	(50.0)	31.2	67.9	98.5	69.3	95.2	n.a.	n.a.	31.6	23.7	59.9	5.6	^599^
	79	0.17	Acetic acid	10 g/L	(50.0)	26.5	83.6	98.8	69.4	95.2	n.a.	n.a.	25.7	9.1	55.0	4.9	^599^
	98	0.25	Acetic acid	10 g/L	0.0	39.4	72.0	98.6	77.7	96.2	n.a.	n.a.	44.7	25.3	54.3	5.1	^599^
	109	0.33	Acetic acid	10 g/L	0.0	34.6	88.0	98.7	81.4	96.3	n.a.	n.a.	35.9	15.8	62.8	6.1	^599^
	65	0.08	Citric acid	10 g/L	0.0	31.8	67.8	98.8	81.7	97.6	n.a.	n.a.	42.7	22.2	64.5	5.8	^599^
	82	0.17	Citric acid	10 g/L	(50.0)	25.8	82.9	99.3	88.5	97.9	n.a.	n.a.	36.9	7.0	59.1	5.4	^599^
	100	0.25	Citric acid	10 g/L	(50.0)	39.6	71.9	98.8	94.7	98.3	n.a.	n.a.	58.9	25.0	60.2	5.6	^599^
	111	0.33	Citric acid	10 g/L	(50.0)	35.8	87.0	99.2	95.6	98.4	n.a.	n.a.	58.7	22.6	52.5	6.8	^599^
Wheat straw	50	2	Acetic acid	1 wt %	n.a.	n.a.	100.0	99.4	71.3	90.0	97.8	62.2	59.3	n.a.	18.5	13.9	^607^
Bagasse^d^	90	2	SCL^a^	pH 2.1	50.0	n.a.	n.a.	93.1	92.0	94.8	25.0	n.a.	n.a.	n.a.	0.0^c^	(3.1)	^633^
Beech wood^d^	90	2	Acetic acid	0.3–1 wt %	n.a.	n.a.	n.a.	98.6	31.8	96.5	99.9	n.a.	n.a.	n.a.	n.a.	n.a.	^642^
	30–90	0.5–2	Acetic acid	5–10 wt %	n.a.	n.a.	n.a.	99.3	60.3	99.1	99.5	n.a.	n.a.	n.a.	n.a.	n.a.	^642^
Hay^d^	90	2	SCL^a^	pH 2.1	(27.3)	n.a.	n.a.	99.6	88.5	95.4	96.5	n.a.	n.a.	n.a.	83.3	9.3	^633^
Pine^d^	90	2	SCL^a^	pH 2.1	100.0	n.a.	n.a.	97.7	96.2	97.7	96.7	n.a.	n.a.	n.a.	100.0	28.4	^633^
Rice straw^d^	25	2	Acetic acid	5 wt %	20.6	6.3	n.a.	99.4	21.8	91.3	81.6	n.a.	54.2	n.a.	47.1	n.a.	^605^
	50	2	Bio-oil^b^	pH 2.9	(14.3)	n.a.	n.a.	99.7	95.2	96.6	91.7	n.a.	n.a.	n.a.	38.3	n.a.	^620^
Straw (batch 1)^d^	90	2	SCL^a^	pH 2.1	(60.0)	n.a.	n.a.	97.6	92.8	97.3	97.4	n.a.	n.a.	n.a.	66.7	(7.3)	^633^
Straw (batch 2)^d^	90	2	SCL^a^	pH 2.1	50.0	n.a.	n.a.	99.6	92.1	96.5	81.2	n.a.	n.a.	n.a.	37.5	3.9	^633^

a SCL is a synthetic condenser liquid, which consisted of 10 wt % acetic acid, 3.75 wt % acetone, 3.75 wt % ethanol, 1.5 wt % guaiacol, 1.5 wt % propionic acid, and 79.5 wt % of demineralized water.

b Bio-oil is composed of several organic compounds and is classified as an organic acid leaching solution because acetic acid is a major component.

c The data was not provided as a table in the literature, it was estimated from the graph.

d Removal efficiencies were taken directly from literature or calculated according to eq 21, based only on the initial raw biomass weight (*m*_*w*_ *=m*_*u*_). n.a. = not applicable (not reported). n.d. = not detectable. Negative removal efficiencies are represented in parentheses and were calculated based on the mineral concentrations reported in the literature.

Water washing is performed at ambient pressure and room temperature or above. At low temperatures, this method partially dissolves substances that are soluble in water (e.g., alkali metals such as Na and K ions, alkaline earth metals such as Mg and Ca ions, Cl, S, and P) depending on the water temperature and duration of the washing procedure.^603–608^ While simple and able to remove soluble ions, water washing has the lowest mineral removal efficiency compared to other methods, with an upper limit to its removal efficiency depending on the type of biomass. Water washing can effectively remove alkali metals such as K and Na from various biomass types, although its efficiency for multivalent ions like Ca and Mg is more limited. In many cases, K removal exceeds 80% except for candlenut wood at 30 °C even under mild or moderate conditions.^120,139,299,609–611^ Na is also substantially removed, often with efficiencies over 70%, except in sugar cane bagasse. In contrast, Ca and Mg removal is typically much lower, generally remaining below 70%, except in select cases such as hybrid poplar (91.0% for Ca) and Douglas fir (77.5% for Mg) at 121 °C.^299,612^ Mineral removal can be more effective at higher temperatures and there is no significant difference in efficacy unless the leaching time is short (e.g., 10 min), suggesting limitations due to the slow dissolution of insoluble solid mineral phases.^603,607^ In addition, water washing is not effective for removing silica, which is abundant in corn stover and rice husk.^588,603,608,613,614^ Therefore, water washing alone is not suitable for demineralizing biomass feedstocks with high silica content, mandating the use of other demineralization methods capable of more comprehensive mineral removal. An advantage of water washing is that it largely preserves biomass structure at temperatures below 60 °C due to its mild nature. However, biomass loss can occur at high temperatures; for example, the amount of extractives and hemicellulose of Douglas-fir and hybrid poplar samples were partially reduced at 120 °C.^603,615^ These various considerations indicate that water washing alone is likely to be insufficient to achieve high removal efficiencies (>97.8%) and concentrations below 100 ppm for the main inorganics (Ca, K, Mg, and Na) and N required in the demineralized biomass.

##### 7.3.3 Mineral Acid Leaching

Biomass pretreatment using dilute acids is widely used to remove inorganic compounds such as AAEMs and other metals (e.g., Fe, Al, Si, Mn).^603–605,607,615,621–623^ Rahbari et al.^163^ carried out a comprehensive study on improving biomass combustion by extracting inorganics (K, Na, Mg, Ca, Cl, Si) using water or acid leaching. The most common industrial leaching types to remove minerals are immersion, immersion with agitation, and spray percolation or pouring of water. From these types of industrial leaching, immersion leaching with agitation had the most efficient removal of minerals. As discussed in Section 7.3.1, acids are expected to promote effective mineral removal because some of the ash-forming compounds in the biomass have higher solubility at lower pH values and because low pH increases the rate of mineral dissolution.^619^ Since not all minerals may be removed with a single acid, acid treatments can be performed in either a single step or in multiple sequential steps.^363,624^ Acid treatments can be classified as mineral acid or organic acid leaching, depending on the type of acidic reagents. Both classifications will be discussed in this and the following sections.

Mineral acid leaching, also known as inorganic acid leaching, uses acids derived from inorganic compounds as leaching agents. Common examples include hydrochloric acid (HCl), nitric acid (HNO_3_), sulfuric acid (H_2_SO_4_), phosphoric acid (H_3_PO_4_), hydrofluoric acid (HF), and perchloric acid (HClO_4_), most of which are classified as strong acids. Table 17 lists quantitative mineral removal efficiencies for mineral acid leaching applied at different conditions and to different feedstocks. This method has been applied for biomass demineralization at varying acid concentrations (0.1–10 wt %) and temperatures, and the efficiency of mineral removal through acid leaching varies depending on the type of biomass and the acid used. K removal is particularly effective, with values exceeding 98% in most cases regardless of acid type or feedstock.^139,143,299,609,625^ Mg and Ca also show high removal efficiencies, ranging from 90% to nearly 100% when strong acids such as HNO_3_, HCl, or H_2_SO_4_ are used at moderate temperatures (25–50 °C).^139,143,299,609,625^ Na removal is more variable, with efficiencies spanning from negative to nearly 100% removal, depending on certain conditions such as biomass type. For instance, HCl and HNO_3_ achieve consistently high removal for multiple minerals, including P, K, Ca, Mg, and Na, across a wide range of biomass feedstocks. H_2_SO_4_ also exhibits high removal efficiencies especially for K, Ca, and Mg.^139,299^

Using the data in Table 17, eq 21 (illustrated in Figure 24), and the initial inorganics concentrations reported in the literature, it is possible to estimate the final inorganic concentrations achievable under specific washing conditions. For instance, the 99.2% effective removal efficiency of K from Barley straw (Table 17) washed with 1 wt % HNO_3_ at 50 °C for 2h, and a 14.5 wt % biomass mass loss, corresponds to an estimated K final concentration of 164 ppm, considering that the initial concentration is 17,562 ppm.^607^ Under the same washing conditions, the 95.1% and 92.8% effective removal efficiencies of Ca and Mg for barley straw in Table 17, and initial concentrations of 4,879 and 1,298 ppm, correspond to a final concentration of 280 and 109 ppm, respectively. Therefore, to achieve a final concentration below 100 ppm, the effective removal efficiencies of K, Ca, and Mg for this specific biomass under similar washing conditions should be higher than 99.5%, 98.3%, and 93.4%, respectively. These examples highlight the importance of considering not only the effective removal efficiency but also the biomass mass loss and initial inorganic concentration when evaluating washing effectiveness.

Table 17 and associated literature indicate that the efficiency of inorganic removal by acid leaching generally improves with increased acidity of the extraction medium and higher temperatures. However, these conditions result in a noticeable reduction in biomass mass and significant changes to its composition.^603,615,622^ Mineral acid leaching effectively removes inorganic components along with other soluble low-molecular-weight components, such as extractives and tannins. Mineral acid leaching also risks removing acid-soluble hydrocarbons, as well as hemicellulose and cellulose components of biomass, which could negatively affect the physicochemical structure of the biomass.^605^ Specifically, hemicellulose is hydrolyzed due to its amorphous and branched structure, while cellulose undergoes partial depolymerization under certain conditions, such as elevated temperatures or low pH, leading to the dissolution of amorphous regions.^603–605,607,615,621–623^ Among the above-mentioned inorganic acids, H_2_SO_4_ and HCl have the greatest potential to damage biomass components. Also, S may bond to the biomass structural components and poison catalysts in downstream processes.^501^ In contrast, HF has the least potential to damage biomass structure, suggesting that it can be a suitable alternative to other inorganic acids, particularly for silica removal. However, HF is more hazardous than other mineral acids due to its potential for damage to the skin, eyes, tissues, or respiratory tract when exposed to vapors or through direct contact. In addition, it is more volatile than other acids and possesses a unique ability to cause systemic toxicity.^626^

##### 7.3.4 Organic Acid Leaching

Organic acid leaching has been widely explored as it has been proposed to overcome some of the issues associated with the structural alteration of biomass structural components by mineral acids.^588,599,603,607,617,621,622,631–634^ Table 18 lists quantitative inorganic removal efficiencies for organic acid leaching applied at different conditions and to different feedstocks. Organic acid leaching using acetic and citric acids has shown promising results in demineralizing various biomass types, achieving high mineral removal efficiencies without perturbing the structure of lignocellulose as much as mineral acids.^588,599,603,617^ For example, leaching with acetic acid (10 g/L or 1 wt %) achieved high mineral removal across multiple feedstocks, with K removal consistently above 98% and Mg removal reaching above 84% in nearly all cases.^603,621,631^ Citric acid also demonstrated high effectiveness (removing over 97% of K and comparable amount of Mg) at a level similar to that of strong mineral acids along with a degree of biomass loss similar to that achieved by water washing.^603,617^

Despite these high removal efficiencies, organic acids caused only modest biomass loss (average 8.3%),^123,139,143,299,609,610,635^ approximately three times lower than the 14.6% reported for mineral acid leaching.^113,120,139,143,299,539,609–612,625,636–639^ However, while organic acids offer advantages in preserving biomass structure, some organic acids (e.g., citric acid,^640^ oxalic acid^641^) have higher costs compared to mineral acids, which may limit their use in large-scale applications. Further studies are needed to assess their economic feasibility and practical use in scaled-up processes.

##### 7.3.5 Treatment with Chelating Agents

Chelating agents can form coordination complexes with inorganic compounds, such as metals, facilitating their removal from biomass. A chelating agent contains multiple electron donor atoms that form coordination complexes with a single metal atom, creating a structure known as a chelate. This process is used to control metal ion concentrations because chelates often have distinct solubilities compared to the free metal ion or chelating agent.^588^ For example, citrate ions have shown potential as chelating agents by forming binary, ternary, and multivalent complexes, depending on the type of metal ion. Additionally, a tetra-dentate compound can form through the coordination of silicate anions with the four carboxylic acid groups, two from each of two citric acid molecules.^588^ These chelating agents thus operate by strongly binding to metal ions to disrupt their interactions with the biomass matrix, thereby effectively extracting minerals while minimizing damage to cellulose and hemicellulose.^588^

Studies have demonstrated the effectiveness of chelating agents like ethylenediaminetetraacetic acid (EDTA) in biomass demineralization. Table 19 lists quantitative inorganic removal efficiencies for chelating agent treatments applied under different conditions and to different feedstocks. In Table 19, Si removal averaged 42.7%, reaching up to 75% with EDTA leaching at around 130 °C, which is higher than the Si removal typically observed with water washing or organic acid leaching (average mineral removal efficiencies for Si are 20.1% and 26.2% for water washing and organic acid leaching, respectively) in Table 16 and Table 18, and with mineral acid (Table 17) which has an even lower Si removal efficiency. Chelating agent treatments also consistently achieved high removal efficiencies for K, Ca, and Mg (≥95%).^132,299^ In one study, the use of EDTA was investigated at temperatures above 65 °C and found to be highly effective for switchgrass, achieving an ash reduction of approximately 87% after 20 min.^599^ EDTA also outperformed other tested chemicals, such as citric acid, acetic acid, and sulfuric acid, in removing silica, all while preserving the structural integrity of the biomass.^599^ Despite their effectiveness, chelating agents are generally more expensive than mineral acids,^643^ which may limit their practical application at larger scales. The cost and availability of these compounds should be considered when evaluating their feasibility, and further studies are needed to assess their scalability and long-term utility in biomass pretreatment.

**Table 19:** Effective Removal Efficiencies of Chelating Agent Treatment on Biomass Feedstocks. Tables 3–5 Show the Ash and Inorganic Content of Raw Biomass.

	Leaching condition						Effective removal efficiency (%)										
Biomass	T (°C)	Time (h)	Acid type	Acid conc.	N	S	P	K	Ca	Mg	Na	Al	Fe	Si	Ash	Biomass loss (wt %)	Ref.
Switchgrass	65	0.08	EDTA^a^	10 g/L	(50.0)	43.6	75.6	95.5	97.4	97.9	n.a.	n.a.	59.5	20.2	56.5	6.9	^599^
	90	0.17	EDTA	10 g/L	(50.0)	40.9	89.9	97.1	98.7	98.7	n.a.	n.a.	65.9	21.6	65.3	6.5	^599^
	112	0.25	EDTA	10 g/L	(50.0)	51.0	76.1	97.0	99.3	98.7	n.a.	n.a.	72.4	53.7	84.3	7.5	^599^
	128	0.33	EDTA	10 g/L	(50.0)	53.9	92.3	97.1	99.5	98.9	n.a.	n.a.	77.3	75.2	87.3	8.0	^599^
Corn stover^c^	130	2	Sodium citrate	0.05 wt %	25.0	84.5^b^	95.5^b^	98.0^b^	82.0^b^	92.0^b^	45.5^b^	89.0^b^	90.0^b^	68.5^b^	67.5	12.0	^588^
	130	2	Sodium citrate	0.1 wt %	12.5	87.0^b^	97.0^b^	99.0^b^	87.0^b^	95.0^b^	59.0^b^	93.0^b^	95.0^b^	69.5^b^	74.0	13.2	^588^
	130	2	Sodium citrate	0.25 wt %	n.a.	95.0^b^	99.5^b^	99.5^b^	93.5^b^	98.0^b^	52.0^b^	96.5^b^	99.2^b^	75.0^b^	n.a.	n.a.	^588^

a EDTA: ethylenediaminetetraacetic acid.

b The data were not provided as a table in the literature; it was estimated from the graph.

c Removal efficiencies were taken directly from literature or calculated according to eq 21, based only on the initial raw biomass weight (*m*_*w*_ *=m*_*u*_). n.a. = not applicable (not reported). n.d. = not detectable. Negative removal efficiencies are represented in parentheses and were calculated based on the mineral concentrations reported in the literature.

##### 7.3.6 Other Treatments

There have also been efforts to pretreat biomass using solvents other than conventional organic solvents or water, such as ionic liquids (ILs) and deep eutectic solvents (DES). ILs are generally defined as solvents with a melting point below 100 °C (often below room temperature)^644^ that are composed solely of large organic cations such as imidazolium, pyrrolidinium, piperidinium, tetraalkylphosphonium, or tetraalkylammonium, and inorganic or organic anions.^645^ ILs can dissolve minerals from biomass, enhancing thermal stability and facilitating lignocellulose processing. However, their potential roles in mineral recovery and recycling remain underexplored, highlighting the need for further research to optimize their application in sustainable biomass demineralization.^646^ DES are mixtures of Lewis (or Brønsted) acids and bases in which proton transfer is incomplete,^647,648^ resulting in liquids with low melting points and unique solvation properties.^646^ DES, especially DES containing acidic species, have demonstrated significant efficiency in removing minerals such as silica and hydroxyapatite, achieving high demineralization rates. However, research on mineral recovery and its impact on DES performance and reuse is limited, requiring further investigation to optimize their application for sustainable biomass processing.^646^

The factors discussed in Table 15 affect conventional leaching methods, but leaching can also be combined with alternative methods. For example, leaching can be integrated with thermochemical and mechanochemical technologies like microwave irradiation or ultrasound to increase mineral removal efficiency and modify intermolecular interactions in the lignocellulosic matrix.^571^ Future studies are needed in such hybrid technologies to understand how the potential synergies afforded by various demineralization methods can be rationally harnessed to enhance mineral removal efficiencies.

##### 7.3.7 MinFree

Most of the studies in the preceding sections were conducted at a lab scale. Recently, an industrial chemical leaching method, MinFree,^34,47,589^ was developed to demineralize biomass by specifically targeting the removal of AAEMs such as K, Ca, Mg, and Mn. The process optimizes both acid consumption and extraction kinetics by controlling parameters such as particle size, acid concentration, temperature, and wash cycles to maximize demineralization efficiency. Each of the process parameters above is based on tests that report trends in mineral removal efficiency similar to those discussed for different lab-scale experiments in Sections 7.3.2–7.3.5. For example, temperature impacts extraction kinetics. Water washing at 80 °C removes K more efficiently than at 24 °C. An optimal molar ratio of hydrogen ion (H^+^) to divalent cation (e.g., Ca^2+^, Mg^2+^) has been reported to lie between 2.0:1 and 2.2:1, ensuring effective ion exchange. Particle size and the number of wash steps also have a substantial impact on mineral removal. The smaller particle size of microchips promotes the diffusion of acid and water into the material, allowing for better extraction of AAEMs due to reduced diffusion path lengths. MinFree thus illustrates how the factors shown to affect biomass demineralization at a lab scale can be carefully considered to guide the design of industrial biomass demineralization processes.

The MinFree process has been scaled to 20 t per day and incorporates multiple acid washing, rinsing through water washing, and dewatering cycles to achieve mineral removal efficiencies of >98.7% for K, >94.5% for Ca, and >97.0% of Mg.^589^ MinFree is thus a robust solution for the pretreatment of biomass for catalytic fast pyrolysis and other upgrading processes. We detail this process here as an example of the industrial-scale implementation of demineralization by combined water washing and acid leaching. The process begins with feedstock preparation to reduce the size of biomass particles to between 1 mm and 32 mm. The prepared biomass is then subjected to acid washing using HNO_3_ solutions, with acid-to-biomass (liquid-to-solid) ratios ranging from 6:1 to 16:1 (wt:wt), acid concentrations between 0.04 and 0.84 wt %, pH values around 1.5–2.5, and washing temperatures varying between 10 to 80 °C.^34,47,589^

Figure 25 illustrates a multistage countercurrent flow washing configuration designed to enhance extraction efficiency by maintaining a larger mineral concentration gradient, represented by the logarithmic mean concentration difference. This gradient facilitates a higher overall mass transfer rate. In this process, biomass (indicated by the continuous brown arrow in Figure 25) moves from left to right. Initially, mineral-rich biomass is washed with an acid stream that has already been used in previous washing steps and is therefore rich in solubilized minerals leached from the earlier wash step. This facilitates preliminary ion exchange between the biomass and the leaching solution. In the next stage, partially demineralized biomass is treated with a fresher acid stream containing fewer dissolved minerals, typically a mixture of acid and rinsewater, allowing for further mineral extraction. Finally, the biomass undergoes a water wash step with fresh water to remove any residual acid and remaining solubilized ions. Compared to cocurrent designs, this counter-current configuration enables greater mineral removal due to the larger concentration gradient between the solid and liquid phases. However, implementing a continuous counter-current system in a laboratory batch setup presents practical challenges, complicating both modeling and scale-up. The process may employ up to three wash cycles to achieve near-complete (>98%) K removal. After washing, the biomass undergoes mechanical dewatering using side-hill screens, screw presses, or pneumatic pumps. This step effectively separates liquid and solid phases, minimizing residual wash solution in the biomass. The demineralized biomass is then dried at temperatures between 50 and 150 °C, depending on material type and moisture removal targets. MinFree has been effectively combined with the BioTCat process to produce aromatics from loblolly pine in the Anellotech plant in Silsbee, TX with over 5,000 h of operation.^649,650^

**Figure 25:**
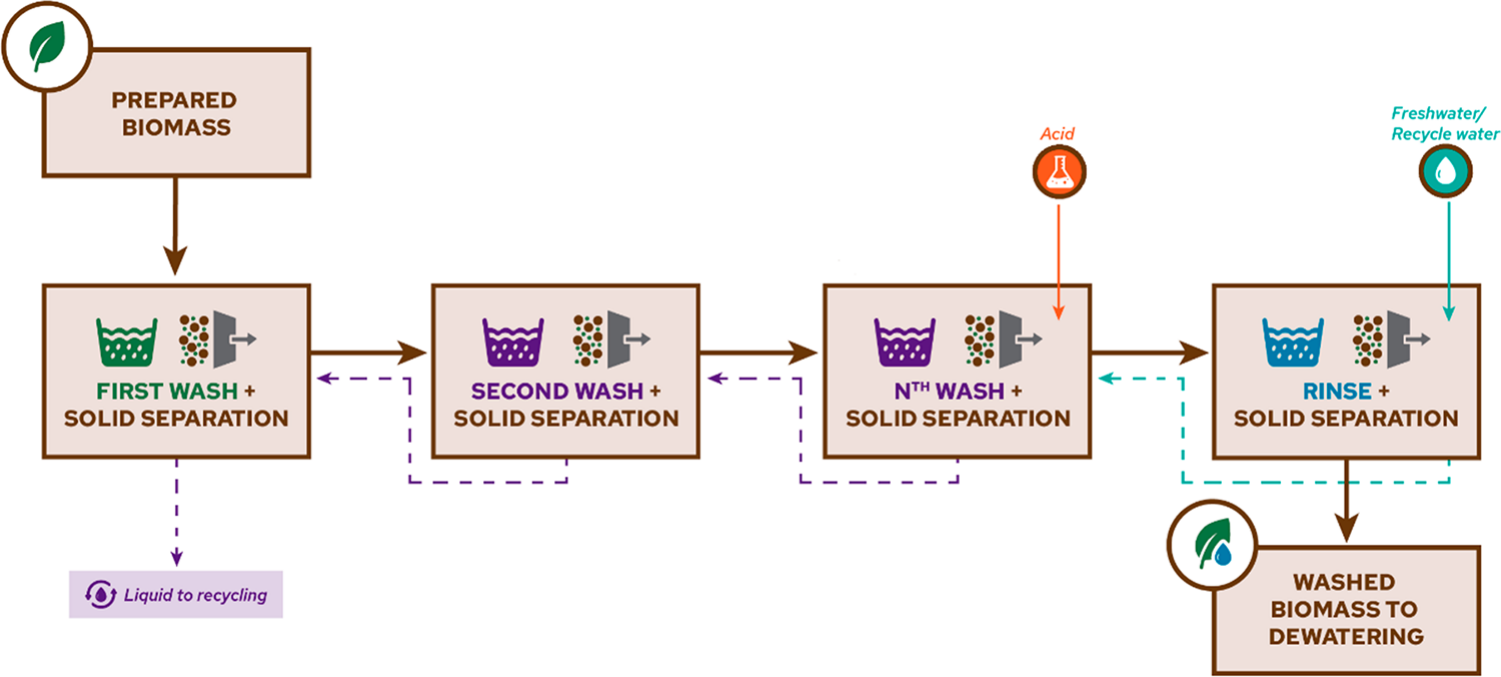
Multistage countercurrent flow washing configuration in MinFree technology.

##### 7.3.8 Summary of Trade-Offs during Inorganics Removal from Biomass and Method Selection

Across leaching-based demineralization strategies, inorganic removal efficiency is inherently coupled to biomass mass loss and preservation of structural components. Water washing primarily removes soluble alkali metals, typically achieving ∼70–85% removal of K and Na, but is considerably less effective for multivalent ions such as Ca and Mg (∼30–45%). Water washing incurs relatively low biomass mass loss, generally around ∼8% under mild conditions. In contrast, mineral acid leaching enables substantially higher removal efficiencies for AAEMs, typically exceeding ∼80–90% for K, Ca, and Mg, but this enhanced demineralization is commonly accompanied by greater biomass mass loss (∼15%), due to dissolution of extractives and partial degradation of hemicellulose and, under severe conditions, cellulose. Organic acid leaching offers a more favorable balance between demineralization and biomass preservation, often achieving very high removal of K (>95–99%) and moderate to high removal of Mg and Na (∼80–95%) with biomass mass loss comparable to water washing (∼8%) and significantly lower than that observed for mineral acid leaching, albeit at higher reagent cost. Finally, treatment with chelating agents enables selective removal of divalent metals and silica, achieving >90–95% removal of Ca, Mg, and K and up to ∼50–75% removal of Si, while largely preserving biomass mass (reported biomass mass loss ∼9%); however, their practical deployment is often constrained by higher reagent costs. Together, these trends highlight that the preferred demineralization strategy depends on balancing required mineral removal levels against acceptable biomass loss and economic and operational constraints.

To guide practical method selection, the decision tree in Figure 26 follows a hierarchical screening logic. The process begins by identifying the biomass feedstock type and initial ash profile and establishing target residual inorganic levels. The minimum required removal efficiency is then used to evaluate whether extrinsic inorganic matter can be sufficiently reduced by a mechanical route (e.g., air classification or sieving). If a mechanical route is not available, a chemical pretreatment pathway is pursued and preservation targets for carbohydrate integrity are defined to constrain treatment severity. Only after these technical targets are established are operational constraints (e.g., corrosion risk, HF avoidance, wastewater burden, reagent cost, and system scale) applied to eliminate infeasible options. The final outcome is a recommended demineralization route or sequence of routes that satisfies both performance and operational requirements for the targeted catalytic or thermochemical process. Table 20 summarizes specific guidelines with references to sections above to further complement Figure 26.

**Figure 26:**
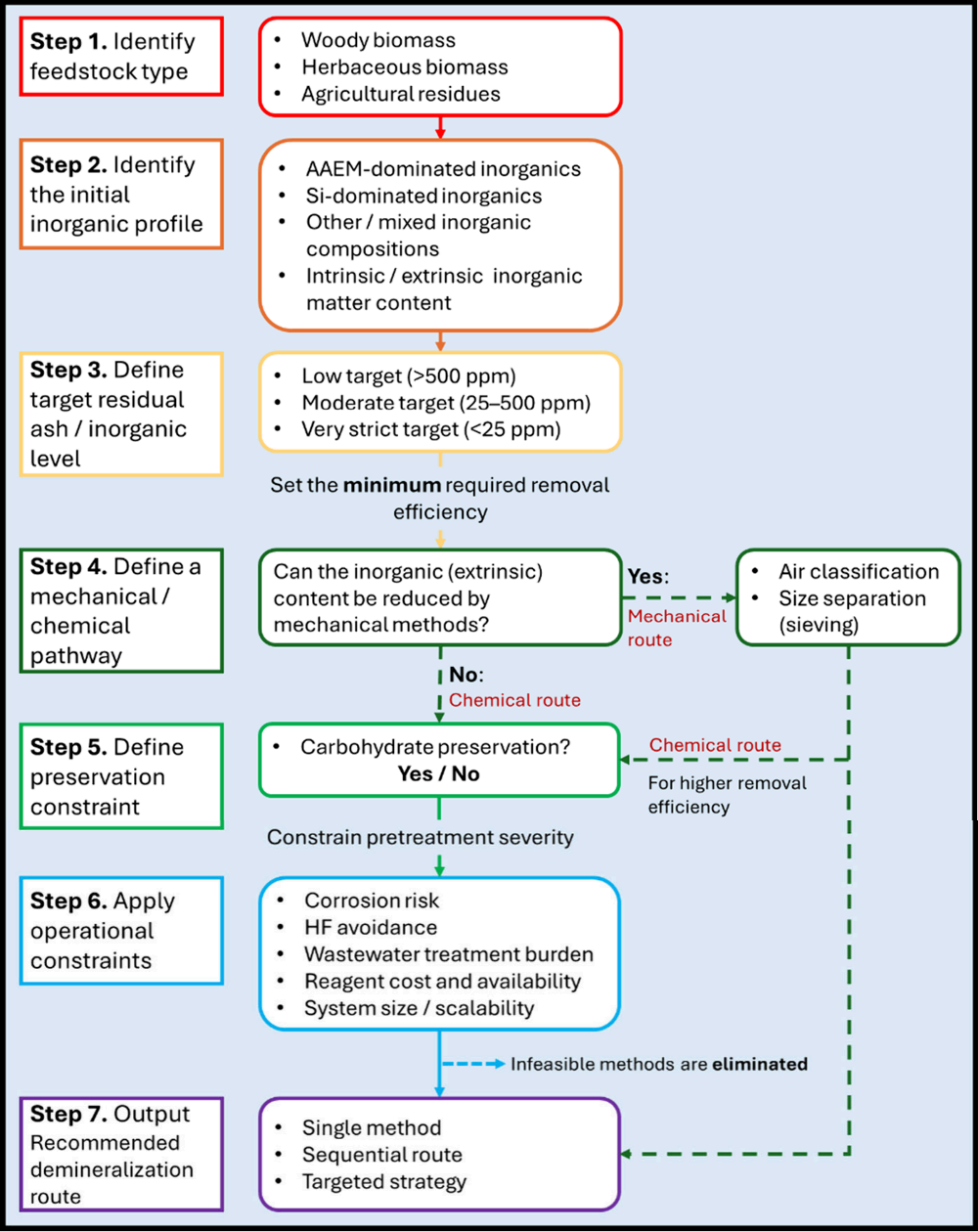
Decision tree for selecting demineralization methods. Method selection proceeds by first identifying feedstock type and initial ash profile, then defining target residual inorganic levels and carbohydrate preservation requirements. Operational constraints are subsequently applied to eliminate infeasible options, yielding a recommended demineralization route or sequence aligned with the targeted catalytic or thermochemical process.

**Table 20:** Practical Guide for Selecting Biomass Demineralization Routes.^a^

Feedstock	Typical ash (wt %) and major inorganics (ppm) (See Section 3)	Recommended route or sequence	Recommended method, solvent, or reagent	Expected removal efficiency	Biomass mass preservation^b^	Anchor (Section)
Woody biomass	Ash: up to 5.1	• Chemical leaching	• Mineral acids: HCl, HNO_3_, H_2_SO_4_	K: >98%	∼90%.	[Sec sec7.1.3.2]–[Sec sec7.1.3.5]
	K: up to 24,000		• Organic acids: Acetic acid, citric acid	Ca: >90% with mineral acids		
	Ca: up to 14,250			<70% with organic acids		
	Mg: up to 11,140			Mg: >96%		
	Si: up to 36,000			Si: <30%		
Herbaceous grasses	Ash: up to 6.8	• Water washing	• Water	K: >98%	∼90% at *T* <85 °C, and ∼67% at higher temperatures and acid concentrations	[Sec sec7.1.3.2]–[Sec sec7.1.3.5]
	Ca: up to 14,000	• Chemical leaching	• Mineral acids: HCl, HNO_3_, H_2_SO_4_	Ca: >90% with mineral acids		
	K: up to 28,826		>56% with organic acids	• Organic acids: Acetic acid, citric acid		
	Mg: up to 1,900		>97% with EDTA			
	Si: up to 7,700		Mg: >90%			
			Si: <30%			
Agricultural residues	Ash: up to 23.5	• Mechanical separation	• Air classification and/or size separation	K: >98%	>80%	[Sec sec7.1.1]
	Ca: up to 15,590	• Water washing	• Water	Ca: >92% with mineral acids		[Sec sec7.1.3.2]–[Sec sec7.1.3.5]
	K: up to 82,930	• Chemical leaching	• Mineral acids: HCl, HNO_3_, H_2_SO_4_	>60% with organic acids		
	Mg: up to 5,467		• Organic acids: Acetic acid, citric acid	>80% with sodium citrate		
	Si: up to 51,862			Mg: >85%		
	Si: <30%					

a Ranges are representative and depend strongly on feedstock, particle size, operating conditions, and demineralization sequence; use subsections cited in the last column for detailed information.

b Biomass loss due to extractives and/or degradation will highly depend on the biomass nature and washing conditions, such as acid type, acid concentration, contact time, and temperature.

##### 7.3.9 Future Directions

Considering the advantages and limitations described above, we highlight several opportunities for future directions of study for effective inorganics removal. Based on the data in Tables 16–19, no single leaching treatment is likely to achieve 100% inorganic removal efficiency for all minerals of interest and for arbitrary feedstocks, particularly when considering potential carbon loss due to the degradation of the biomass. Moreover, pretreatment strategies may also be dictated by specific downstream biomass processing requirements. Hence, stepwise removal strategies, possibly combining multiple leaching treatments, should be further investigated to enhance inorganics removal efficiency while minimizing adverse effects on biomass composition and downstream processing. Such processes should also consider the removal of other biomass components, such as proteins, that may have value if recovered. For example, a potential stepwise process could begin with an alkaline treatment to solubilize proteins by altering their charge states at alkaline pH.^651^ Subsequently, an acidic treatment can be applied to target and dissolve mineral components. The acid type and concentration should be selected carefully to avoid excessive degradation of the biomass matrix and operational challenges associated with the use of strong acids.^603–605,607,621–623^ A thorough water rinse is essential as the final step to remove residual leaching agents and dissolved impurities,^589^ ensuring the treated biomass is compatible with downstream applications (e.g., return to the soil).

To systematically evaluate and optimize the performance of various leaching treatments and aid in process design, quantitative metrics of inorganic removal efficiency should be consistently calculated and reported, as should additional metrics quantifying biomass degradation to move beyond existing metrics like severity factors.^596,599^ Such metrics also permit evaluation of different demineralization processes by using tools from process systems design that address both economic and environmental considerations^652,653^ by combining techno-economic analysis (TEA) with analysis of the environmental impacts of a process. Integrating quantitative metrics for inorganic removal efficiency and severity with process design tools could facilitate the development of selective leaching strategies tailored to various biomass types, improving process specificity while maintaining sustainability or assessing related trade-offs. This integration is expected to improve the overall performance of the biomass pretreatment process by ensuring a balance between effective demineralization and operational feasibility.

##### 7.3.10 Kinetic and Thermodynamic Modeling of Biomass Demineralization

The demineralization processes described in Section 7.3 have largely been developed empirically. Given the large number of process parameters (Table 15), however, there is a need for further computational studies to guide process design to reduce laborious experimentation by quantifying the thermodynamics and kinetic factors affecting demineralization, as discussed in Section 7.3.1. While we are unaware of comprehensive computational studies of the thermodynamics or kinetics of inorganic removal that have been reported to date, we summarize here potential methods that could be valuable for informing demineralization processes.

Thermodynamic modeling of mineral solubility can be valuable for understanding demineralization approaches. The aqueous solubility of a variety of minerals can be predicted using thermodynamic methods that have been developed to model mineral dissolution or speciation in aqueous environments.^578,654,655^ These processes are typically modeled using methods to predict chemical reaction equilibrium based on tabulated free energies for the formation of different mineral phases, with equation of state models used to account for variations in the thermodynamic activity of dissolved species. Such approaches have the potential to predict mineral solubility and undersaturation as a function of temperature and pH^656,657^ to identify conditions that enhance dissolution, potentially promoting more efficient mineral removal. A key challenge lies in optimizing the simultaneous dissolution of multiple minerals that are typically present in biomass.

To complement macroscopic thermodynamic modeling, molecular-scale modeling methods, such as molecular dynamics (MD) simulation, can provide more detailed insight into specific interactions between leaching agents (e.g., chelating agents or unique solvents like ILs or DES) and biomass components. MD simulations offer the potential to resolve complex interactions between leaching agents, solvents, other dissolved species and biomass constituents (e.g., hemicellulose, cellulose, or lignin) that may influence demineralization thermodynamics. For example, MD simulations can be used to compute solvation free energies of dissolved ions to account for their thermodynamic activities in these complex environments to complement macroscopic equation-of-state models. Similarly, MD simulation methodologies may be used to quantify free energy barriers associated with disrupting mineral interactions within the biomass matrix. In addition, some metals form complexes with proteins that can be studied using MD simulations to provide molecular-scale insight into processes for removing structural minerals using leaching strategies.

From a kinetic modeling perspective, macroscopic models can be used to capture the effect of various factors influencing the rate of demineralization. Per the discussion in Section 7.3.1, both rates of diffusion (transport) and dissolution are expected to impact the overall kinetics of demineralization and are affected by factors including particle size, temperature, intrinsic reaction kinetics, and pH, as described in the preceding sections. Similar factors are also known to affect mineral dissolution in porous environments like soil, for which simultaneous reaction-transport models have been developed to model mineral dissolution and precipitation.^658^ These methods merge continuum mass transfer models and reaction kinetics models using experimentally determined parameters. We envision similar modeling strategies could be adapted to optimize biomass demineralization process parameters and evaluate whether demineralization is limited by transport or dissolution rates. At a process level, batch and semicontinuous leaching processes also exhibit distinct diffusion behaviors. Batch leaching allows prolonged interaction, allowing the diffusion of both water-soluble and water-insoluble species at progressively lower rates. In contrast, semicontinuous leaching can maintain higher concentration gradients by removing the leached species, enhancing overall mineral removal rates. Batch leaching often follows a two-step kinetic profile, while semicontinuous processes typically exhibit a single-step mechanism dominated by rapid diffusion.^659^ Accounting for differences in the overall kinetics associated with these processes can similarly inform process design, particularly when combined with the assessment of overall process economics.

Based on these considerations, there is a need to establish a comprehensive framework to optimize mineral removal strategies in biomass processing by integrating thermodynamic modeling, molecular-scale modeling, kinetic modeling, and process modeling, building upon the conceptual framework provided in Section 7.3.1. As a future direction, these models can be developed and refined to account for diverse biomass feedstock compositions/structures, leaching agents, and operating conditions, allowing more targeted and efficient demineralization processes. Additionally, modeling efforts must account for various species and interactions that inorganic elements can exhibit within the biomass structure. These include water-soluble salts, acid-soluble minerals, and organically associated elements, such as ionically bonded metal ions and covalently bonded nonmetals. To better understand and quantify these forms and their interactions, chemical fractionation analysis^9,160,558,660–663^ (i.e., sequential leaching in H_2_O, NH_4_Ac­(aq), and HCl­(aq)) plays a crucial role. This method enables a more detailed characterization of inorganic compound in lignocellulosic biomass, thereby enhancing the accuracy and predictive power of biomass demineralization models.

### Section Summary

In this section, we summarized different approaches for removing minerals from lignocellulosic biomass to mitigate the detrimental effects of inorganics in biomass conversion technologies. These approaches include mechanical separations (air classification and sieving), torrefaction as a pretreatment method that can enhance mineral removal by opening the biomass structure, and chemical leaching using various leaching agents, such as mineral acids, organic acids, and chelating agents.

## 8 Conclusions

Lignocellulosic biomass represents an important source of feedstock for chemicals, fuels, and biobased materials, and will play a critical role in transitioning to a low-carbon economy. Lignocellulosic biomasses have a variable content of inorganic elements typically characterized in the form of ash, and their concentration highly depends on environmental and growing conditions, harvesting and handling, and the plant fraction. Among terrestrial biomasses, agricultural residues have the highest (8.4 wt %) average ash content, followed by herbaceous biomass (4.3 wt %), and woody biomass (2 wt %). On average, K, Ca, and Si are the majority (>10,000 ppm) contributors to the biomass ashes, followed by Mg, Na, and Al (<10,000 ppm), Fe (<5,000 ppm), and Mn and Zn (<100 ppm). Nonmetal elements such as N (<15,000 ppm), Cl (<10,000 ppm), and S and P (<5,000 ppm) are also important components in lignocellulosic biomasses. All these elements enter biomass via root absorption in the form of soluble minerals (inherent minerals) or are incorporated into the biomass during harvesting and handling (extrinsic inorganics), and they pose different challenges in biomass processing technologies.

The presence of silica can cause wear in biomass preprocessing equipment during milling, sieving, handling, and feeding. The combination of silica, aluminosilicates, alkali and alkaline earth metals, sulfur, chlorine, and carbonates causes agglomeration, slagging, fouling, plugging, and corrosion of process equipment during thermochemical biomass conversion, especially during combustion, gasification, and pyrolysis. The formation of deposits along the process equipment can cause reduced heat transference, higher resistance to fluid flow, and a higher pressure drop, with detrimental effects on heat exchanger efficiencies, maintenance costs, and useful lifetime.

The inorganics in biomass also affect product yields and selectivity during gasification, pyrolysis, and hydrothermal processing of biomass. Inorganics can play a catalytic role in the formation, decomposition, and transformation of tars during gasification. Depending on the biomass, the inorganics concentration, the chemical form of minerals, and the reaction conditions, these catalytic effects can be highly variable. Alkali and alkaline earth metals can significantly catalyze the release and conversion of nitrogen-containing species to form NO_*x*_, whose emissions contribute to environmental pollution and can further react to form nitric acid, leading to severe corrosion of downstream equipment. Inorganics can have a similar effect during pyrolysis and hydrothermal processing, producing variations in product selectivities and yields. Elements like Mg, Ca, K, and Fe can catalyze the breakdown of polysaccharides and lignin, and lower the decomposition temperatures of these biomass components.

In catalytic processes such as catalytic pyrolysis, bio-oil hydrodeoxygenation, biogas reforming, and reductive catalytic fractionation, these inorganic elements, in addition to N, Cl, S, and P, react with the catalyst structure, modify the active sites, and cause permanent deactivation of catalysts through ion exchange, which decreases the catalyst life. In biochemical biomass conversion and fractionation, minerals reduce the effectiveness of dilute acid pretreatments, reduce the accessibility of enzymes due to the formation of complexes with hemicellulose, cellulose, or lignin, and create unfavorable reaction conditions during enzymatic hydrolysis, fermentation, and anaerobic digestions.

Our recommendations for future research are

1. Understand how biomass harvesting methods can impact the inorganics/mineral content. The inorganic content in the biomass depends on how the biomass is harvested and the time of year that it is harvested. Implementing new harvesting techniques can reduce the ash contamination to intrinsic structural ash of the plant and avoid soil contamination.^369^
2. Understand how inorganics are distributed throughout various types of biomasses. Minerals can be in different phases within the biomass. It is critical to understand where they are located and how they change during the biomass conversion process. Imaging studies can be helpful with understanding the location of the minerals.
3. Understand the coordination between inorganics/minerals and biomass components (cellulose, hemicellulose, and lignin), as it influences their thermal stability and accessibility to enzymes and microorganisms. Such interactions directly impact the efficiency of both thermal and biochemical conversion processes of lignocellulosic biomass.
4. Understand how inorganics can be removed from biomass by chemical treatment, thermal treatments and mechanical separation. As summarized in Section 7, chemical treatments can include washing with different leaching agents such as mineral and organic acids, chelating agents, deep eutectic solvents, and ionic liquids; thermal treatments can include HTL, HTC, and torrefaction; and mechanical separation such as air classification, grinding, and sifting.
5. Develop strategies to return minerals to the soil in the form of soil amendments or to recover minerals with economic importance by precipitation, adsorption, or concentration. Liquid soil amendment formulations should consider the effects in the soil such as pH changes, leaching effects, and contamination by undesired elements.^664–670^

6. Develop a detailed understanding of the thermodynamics of inorganics during thermochemical and aqueous conversion of biomass. This includes identifying the composition and phases of different minerals.
7. Develop a fundamental understanding on the relationship between inorganic content in biomass and the inorganic impact on the thermochemical conversion of biomass. Inorganics have been reported to both promote and hinder gasification^199,227–230,233,238,239^ and pyrolysis^286–288,303,671,672^ reactions as described in Section 4.2.2. The inorganics can foul process equipment. A better understanding of the role of the inorganics is needed.
8. Develop a detailed model for the impact of inorganics on catalysts during biomass conversion. A better understanding on how inorganics can poison different types of catalysts is needed. This includes developing a relationship between the inorganics and the catalyst stability by testing them for larger times on stream and regeneration cycles.
9. Develop a fundamental understanding of the impact of inorganics on biological conversion of biomass. As described in Section 5, inorganics can influence biomass pretreatment, reduce enzymes accessibility, and limit other biological reactions.
10. Integrate demineralization of biomass with downstream biomass conversion technologies. Advanced technologies for demineralization of biomass are needed that can economically remove inorganics. These technologies need to be combined with different biomass downstream technologies. Develop process modeling that integrates supply chain models to determine the economic and environmental impacts of biomass demineralization. Currently, only a few studies^564,673–676^ have reported a cost analysis or inorganics removal from biomass.

In pursuing future research directions, state-of-the-art measurement, computational and automation tools should be harnessed effectively for elemental characterization and kinetic studies to gain a reliable fundamental understanding of the impacts of inorganics and demineralization on downstream biomass conversion processes, thereby expediting discovery and innovation.

Below, we list a brief playbook to guide biomass demineralization strategies based on the present review:

1. Characterize biomass inorganic content using a well-standardized analytical methodology, such as inductively coupled plasma (ICP) spectroscopy and ash determination.
2. Define a target of inorganic content in biomass according to the downstream process requirements or application and set the minimum required removal efficiency for each inorganic element of interest.
3. Identify whether extrinsic inorganics can be removed by means of mechanical methods such as size separation or air classification, especially for herbaceous biomass and agricultural residues.
4. Define constraints on pretreatment severity, based on equipment corrosion risk, safety concerns on the use of hazardous chemicals (i.e., HF), wastewater treatment, economic viability, and system size or scalability.
5. Define a recommended demineralization route, including mechanical and/or chemical methods, in a single or sequential demineralization strategy to achieve the targeted demineralization efficiency.
6. Apply the defined demineralization strategy and verify if the target was achieved using a validated method, as mentioned in point number 1.
7. Find the best approach to use the inorganics removed from biomass for soil amendments, either as a concentrated liquid solution or as a formulation using a solid adsorbent.

## Abbreviations

AAEMs: Alkali and alkaline earth metals
AD: Anaerobic digestion
ATP: Adenosine triphosphate
BS: Below screen
BTX: Benzene, toluene, and xylenes
CEC: Cation exchange capacity
CFP: Catalytic fast pyrolysis
cp: centipoise
CRISPR: Clustered regularly interspaced short palindromic repeats
CV: Number of catalyst bed volumes of bio-oil processed
DES: Deep eutectic solvents
DIET: Direct interspecies electron transfer
DLCA: Densifying lignocellulosic biomass with chemicals followed by autoclave
DNA: Deoxyribonucleic acid
DOE: US Department of Energy
ECN: Energy Research Center of The Netherlands
EDS: Energy-dispersive X-ray spectroscopy
EDTA: Ethylenediaminetetraacetic acid
EFB: Empty fruit bunches
FCC: Fluid catalytic cracking
FID: Flame ionization detector
FMEA: Failure mode and effects analysis
FTS: Fischer–Trøpsch synthesis
GC: Gas chromatography
HDAL: Hydrothermal dealumination
HDO: Hydrodeoxygenation
HHV: Higher heating value
HTC: Hydrothermal carbonization
HTL: Hydrothermal liquefaction
HTP: Hydrothermal processing
ILs: Ionic liquids
LHV: Lower heating value
LHWP: Liquid hot water pretreatment
MCR: Methyl-coenzyme M reductase
MD: Molecular dynamic simulation
MFSP: Minimum fuel sale price
MS: Mass spectrometry
MSW: Municipal solid waste
MW: Megawatt
NADPH: Nicotinamide adenine dinucleotide phosphate
PAHs: Polycyclic aromatic hydrocarbons
PID: Photoionization detector
PRB: Powder river basin
RNA: Ribonucleic acid
RZT: Root zone temperature
SCWG: Supercritical water gasification
SEM: Scanning electron microscopy
SPA: Solid-phase adsorption
SPME: Solid-phase microextraction
TEA: Techno-economic analysis
TEM: Transmission electron microscopy
TGA: Thermogravimetric analysis
TOS: Time on stream
UV-LED: Ultraviolet light-emitting diodes
VFD: Variable frequency drive
VGO: Vacuum gas oils
VGR: Vacuum gas residues
wt %: Weight percentage
WWS: Waste wheat straw
XOS: Xylooligosaccharides
XPS: X-ray photoelectron spectroscopy
XRD: X-ray diffraction
